# Immune-Related Adverse Events Associated with Immune Checkpoint Inhibitors: A Scoping Review

**DOI:** 10.3390/ph19020276

**Published:** 2026-02-06

**Authors:** Costanza Tacchi, Irma Convertino, Guido Bocci

**Affiliations:** 1 Department of Translational Research and New Technologies in Medicine and Surgery, University of Pisa, 56126 Pisa, Italy; costanza.tacchi@phd.unipi.it (C.T.); guido.bocci@unipi.it (G.B.); 2 Unit of Pharmacology and Pharmacovigilance, Department of Clinical and Experimental Medicine, University of Pisa, 56126 Pisa, Italy; 3 Regional Pharmacovigilance Center of Tuscany, 50139 Florence, Italy

**Keywords:** immune-related adverse events, immune checkpoint inhibitors, administrative healthcare databases, spontaneous reporting systems, Food and Drug Administration, European Medicines Agency

## Abstract

**Background**: The heterogeneity of immune-related adverse events (irAEs) in real-world evidence highlights the need to identify patterns, knowledge gaps, and priorities for future research. **Objectives**: To assess in labels the expected irAEs associated with immune checkpoint inhibitors (ICIs) in lung cancer, melanoma, breast cancer, and colon cancer and evaluate their incidence, clinical characteristics, management, and outcomes in real-world studies. **Methods**: Medicine Agency data sources (Food and Drug Administration and European Medicines Agency) were assessed for labeled irAEs associated with ICIs, and a comprehensive literature review according to Preferred Reporting Items for Systematic reviews and Meta-Analyses (PRISMA) guidelines for scoping review was performed by retrieving observational and target trial emulation studies conducted using data collected in administrative healthcare databases (AHDs) and in spontaneous reporting systems (SRSs) concerning the drugs and tumors of interest from PubMed. irAEs’ incidence, onset, management, and outcomes were retrieved. **Results**: ICI combination therapy increases irAE occurrence, and inter-agency differences emerged. From PubMed, 49 observational studies were included, 22 on SRSs and 27 on AHDs. The ICIs most frequently evaluated were pembrolizumab and nivolumab, and the irAEs most reported were “lower respiratory tract disorders (excluding obstruction and infection)” (SRSs) and “epidermal and dermal conditions” (AHDs) for both drugs. Missing information on survival analysis, therapy dechallenge and rechallenge, concomitant therapies, comorbidities, time to onset, and duration of irAEs were highlighted. **Conclusions**: This scoping review highlights the complex, multi-organ irAEs from ICIs, underlining the need for tailored monitoring and management based on both regulatory and real-world evidence.

## 1. Introduction

Immune checkpoint inhibitors (ICIs) have revolutionized the treatment of patients with different types of tumors, like melanoma, lung cancer, breast cancer, and colon cancer, by enhancing the immune system’s ability to recognize and eliminate tumor cells [1,2]. ICIs include monoclonal antibodies targeting cytotoxic T-lymphocyte antigen 4 (CTLA-4), like ipilimumab, and programmed cell death protein-1 (PD-1), like pembrolizumab, nivolumab, and cemiplimab, or its ligand-1 (PD-L1), such as atezolizumab and durvalumab, which have significantly improved survival rates in various malignancies, including melanoma and non-small cell lung cancer [1,2]. Immunotherapies are designed to engage the immune system to generate antitumoral activity, and due to this mechanism, they can lead to the activation of T cells [3]. However, the use of ICIs is often associated with immune-related adverse events (irAEs), which can negatively affect the treatment outcome and impact the patient’s quality of life. IrAEs are a spectrum of adverse drug events (ADEs) resulting from the activation of the immune system against normal tissues. Common irAEs include endocrine, cutaneous, gastrointestinal (GI), hepatic, neurological, and pulmonary impairments, which may be life-threatening or fatal [1,4,5,6]. The incidence and severity of irAEs vary depending on the specific ICI and the cancer type being treated [6]. The pathological mechanisms underlying these ADEs are not fully understood but the involvement of an imbalance in immune regulation leading to autoimmunity cannot be excluded [1,4]. Evidence in medical literature have highlighted the chronic nature of some irAEs, with reports indicating that certain damage can persist for months or even years after discontinuation of ICI therapy, as for instance occurred for chronic non-endocrine irAEs in patients who had received ICIs, revealing that a significant proportion experienced prolonged symptoms requiring ongoing clinical management [7]. This underlines the need for continuous monitoring and developing predictors to better identify and manage these ADEs since the clinical implications of irAEs are treatment discontinuation, a heavy burden of the disease, and increased healthcare costs [2,7,8].

Some gaps in knowledge are about their long-term effects, risk factors for development, and effective management protocols. The heterogeneity of reported irAEs across different studies complicates efforts to establish standardized definitions and treatment guidelines. As such, there is an urgent need to identify trends, gaps in knowledge, and areas for future medical research.

This scoping review aims to assess in labels the current expected irAEs associated with ICIs in lung cancer, melanoma, breast cancer, and colon cancer and to evaluate their incidence, clinical characteristics, management strategies, and long-term outcomes in real-world studies.

## 2. Results

### 2.1. Landscape of the Regulatory Authorities: EMA and FDA

In Table 1 and Supplementary Materials Table S1, we display the summary and the detailed information reported in the specific product characteristic (SPC) of each ICI common to both the European Medicines Agency (EMA) and Food and Drug Administration (FDA), while in Table 2 and Table S2 the respective information specific to each agency, both using the Medical Dictionary for Regulatory Activities (MedDRA) high-level group terms (HLGTs) for reporting the irAEs. In the Tables S3 and S4, we provide all the above-mentioned detailed information using High Level Terms (HLTs) for the irAEs.

“Adrenal gland disorders”, “central nervous system infections and inflammations”, “epidermal and dermal conditions”, “exocrine pancreas conditions”, “gastrointestinal inflammatory conditions”, “gastrointestinal motility and defecation conditions”, “gastrointestinal signs and symptoms”, “glucose metabolism disorders (including diabetes mellitus)”, “hepatic and hepatobiliary disorders”, “hypothalamus and pituitary gland disorders”, “immune disorders NEC (not elsewhere classified)”, “joint symptoms”, “lower respiratory tract disorders (excluding obstructions and infection)”, “muscle disorders”, “myocardial disorders”, “nephropathies”, “neuromuscular disorders”, “ocular infections, irritations and inflammations”, “peripheral neuropathies”, “procedural related injuries and complications NEC”, “thyroid gland disorders”, and “vascular infections and inflammations” are the irAEs reported by both agencies and common for all the drugs of interest (Table 1 and Table S1).

Here, below, for each drug, we report the irAEs identified by both EMA and FDA other than the ones common to all ICIs of interest.

Ipilimumab, a CTLA-4 (cytotoxic T-lymphocyte associated protein 4) inhibitor, was the first ICI approved in 2011, and it can be used in monotherapy for the treatment of melanoma or in combination with nivolumab for melanoma and for other types of tumors not of our interest, such as non-small cell lung cancer (NSCLC) and colon cancer. “Endocrine disorders of gonad function”, “pericardial disorders”, “demyelinating disorders”, “gastrointestinal investigation”, “ocular structural change, deposit and degeneration NEC”, “renal disorders (excluding nephropathies)”, “connective tissue disorders (excluding nephropathies)”, “general system disorders NEC”, “joint disorders”, “infections-pathogen unspecified”, “diabetic complications”, “gastrointestinal hemorrhages NEC”, “vascular disorders NEC”, “ocular infections, irritations and inflammations”, “hearing disorders”, and “white blood cell disorders” are among the irAEs reported by both the agencies for ipilimumab monotherapy (Table 1 and Table S1).

Nivolumab is a PD-1 (programmed cell death-1) inhibitor, authorized by FDA in 2014 and by EMA in 2015 for several treatments in monotherapy, including melanoma and NSCLC. IrAEs expected for nivolumab by both EMA and FDA are “connective tissue disorders (excluding nephropathies)”, “demyelinating disorders”, “diabetic complications”, “gastrointestinal hemorrhages NEC”, “gastrointestinal investigation”, “general system disorders NEC”, “hemolysis and related conditions”, “hepatobiliary investigations”, “joint disorder”, “oral soft tissue conditions”, “parathyroid gland disorders”, “pericardial disorders”, “renal disorders (excluding nephropathies)”, and “white blood cell disorders” (Table 1 and Table S1).

The combination of ipilimumab and nivolumab is associated with a combination of irAEs accepted by the two agencies for the two drugs in monotherapy (Table 1 and Table S1).

Pembrolizumab, another PD-1 inhibitor, was authorized by FDA in 2014 and by EMA in 2015 for several different cancer types, including melanoma (in monotherapy), NSCLC (in monotherapy and in combination with other therapies), colorectal cancer (in monotherapy), and triple-negative breast cancer (in combination with other therapies). “Bile duct disorders”, “connective tissue disorders (excluding nephropathies)”, “diabetic complications”, “gastrointestinal hemorrhages NEC”, “gastrointestinal investigation”, “gastrointestinal ulcer and perforation”, “hemolysis and related conditions”, “hepatobiliary investigations”, “infections-pathogen unspecified”, “joint disorders”, “oral soft tissue conditions”, “parathyroid gland disorders”, “pericardial disorders”, and “renal disorders (excluding nephropathies)” were reported by EMA and FDA alike as irAEs for pembrolizumab (Table 1 and Table S1).

Cemiplimab, a PD-1 inhibitor, approved in 2018 by FDA and in 2019 by EMA for tumors like NSCLC, has “connective tissue disorders (excluding nephropathies)”, “diabetic complications”, “hepatobiliary investigations”, “joint disorders”, “oral soft tissue conditions”, “pericardial disorders”, and “renal disorders (excluding nephropathies)” as irAEs expected by the two agencies (Table 1 and Table S1).

Atezolizumab is a PD-L1 (programmed cell death-1) inhibitor authorized by FDA for NSCLC, SCLC, and melanoma, among other tumors not of our interest, in 2016 and by EMA for NSCLC, small-cell lung cancer (SCLC), and triple-negative breast cancer (TNBC) and other tumors in 2017. “Connective tissue disorders (excluding nephropathies)”, “diabetic complications”, “gastrointestinal hemorrhages NEC”, “gastrointestinal investigation”, “hepatobiliary investigations”, “oral soft tissue conditions”, and “pericardial disorders” are irAEs reported in this case (Table 1 and Table S1).

Durvalumab, a PD-L1 inhibitor, was approved in 2017 by FDA and in 2018 by EMA for the treatment of NSCLC, SCLC, and other types of tumors not of our interest. “Anemias nonhemolytic and marrow depression”, “bile duct disorders”, “diabetic complications”, “gastrointestinal hemorrhages NEC”, “gastrointestinal investigation”, “gastrointestinal ulcer and perforation”, “hemolysis and related conditions”, “hepatobiliary investigations”, “injuries by physical agents”, “joint disorders”, and “oral soft tissue conditions” are irAEs reported by the agencies for this drug [9,10] (Table 1 and Table S1).

Regarding differences between the two agencies, for example, EMA reported “bladder infections and inflammations” for all the drugs, whereas the FDA reported “marrow depression and hypoplastic anemias” for all the drugs that EMA reported only for durvalumab. All the differences are summarized in Table 2 and in detail in Table S2 [9,10].

### 2.2. Real World Evidence

**Figure 1:**
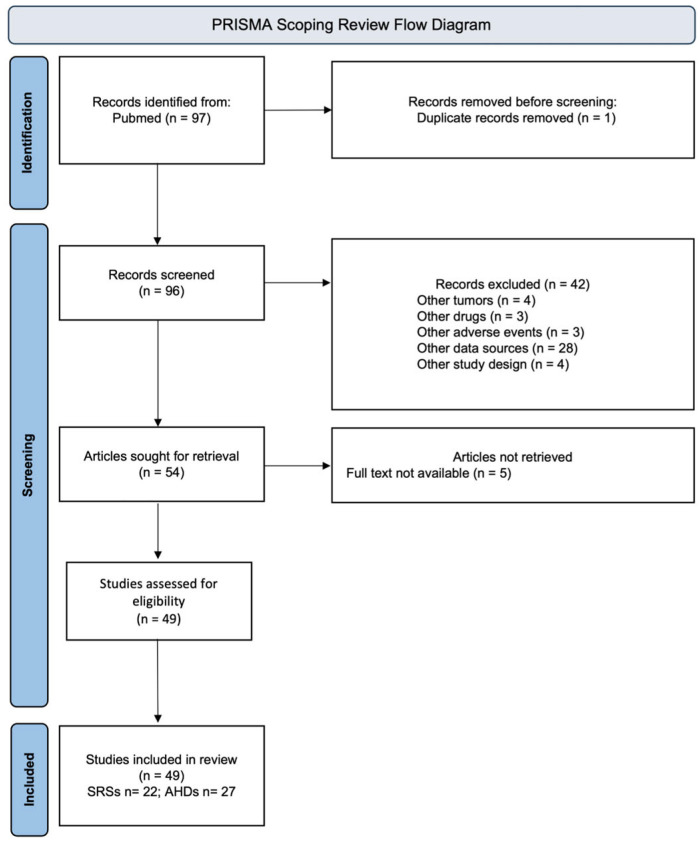
Flowchart of the included studies. SRSs spontaneous reporting systems; AHDs administrative healthcare databases.

A total of 97 articles were found from the research conducted on PubMed, and 49 observational studies [11,12,13,14,15,16,17,18,19,20,21,22,23,24,25,26,27,28,29,30,31,32,33,34,35,36,37,38,39,40,41,42,43,44,45,46,47,48,49,50,51,52,53,54,55,56,57,58] were included in this review (see Figure 1). No target trial emulation studies were selected.

In our research, 22 studies [32,33,34,35,36,37,38,39,40,41,42,43,49,50,51,52,53,54,55,56,57,58] were retrieved from spontaneous reporting systems (SRSs) and the remaining 27 studies [11,12,13,14,15,16,17,18,19,20,21,22,23,24,25,26,27,28,29,30,31,33,44,45,46,47,48] from administrative healthcare databases (AHDs).

#### 2.2.1. Evidence from Spontaneous Reporting Systems

**Figure 2:**
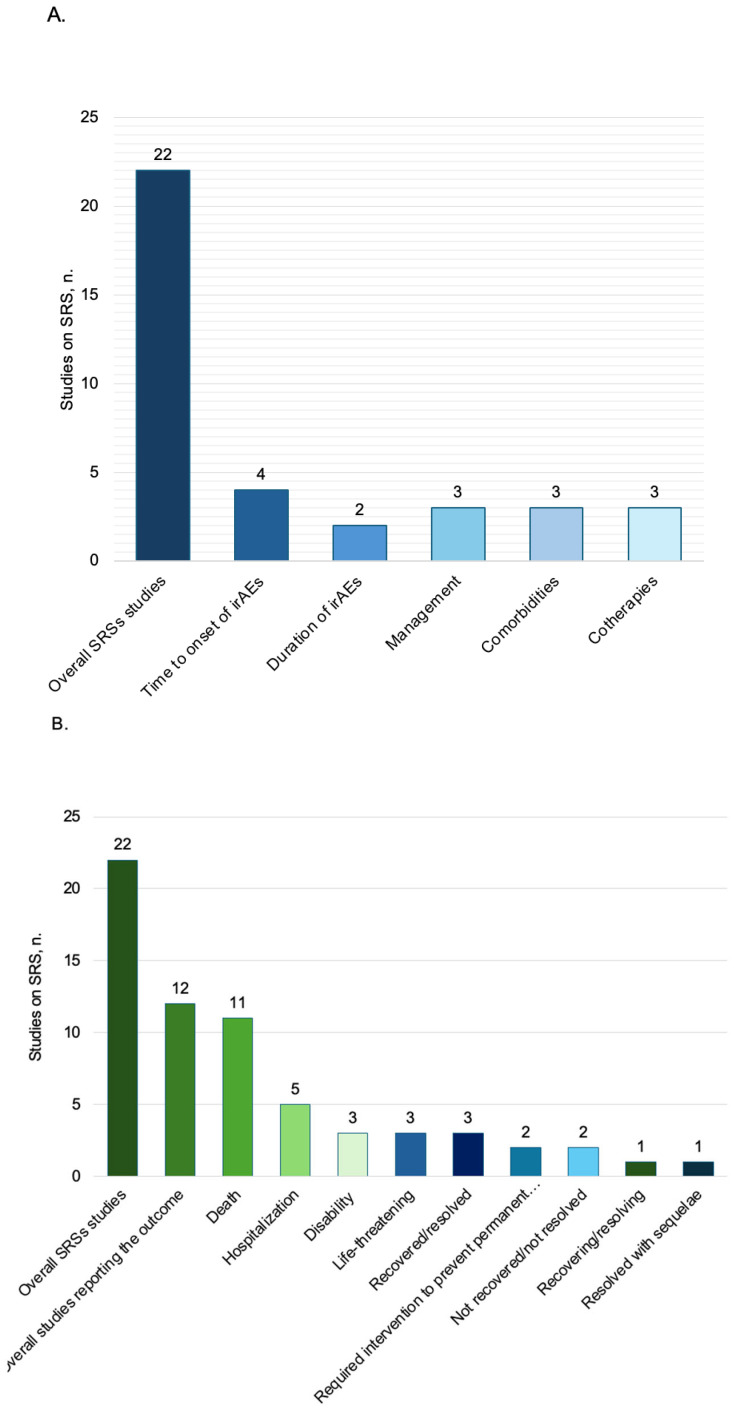
Distribution of studies conducted on SRSs. (**A**) Assessing the time to onset of irAEs, duration of irAEs, comorbidities, and concomitant therapies. (**B**) Assessing the outcome of irAEs. SRSs spontaneous reporting systems; irAEs immune-related adverse events; AHDs administrative healthcare databases.

**Figure 3:**
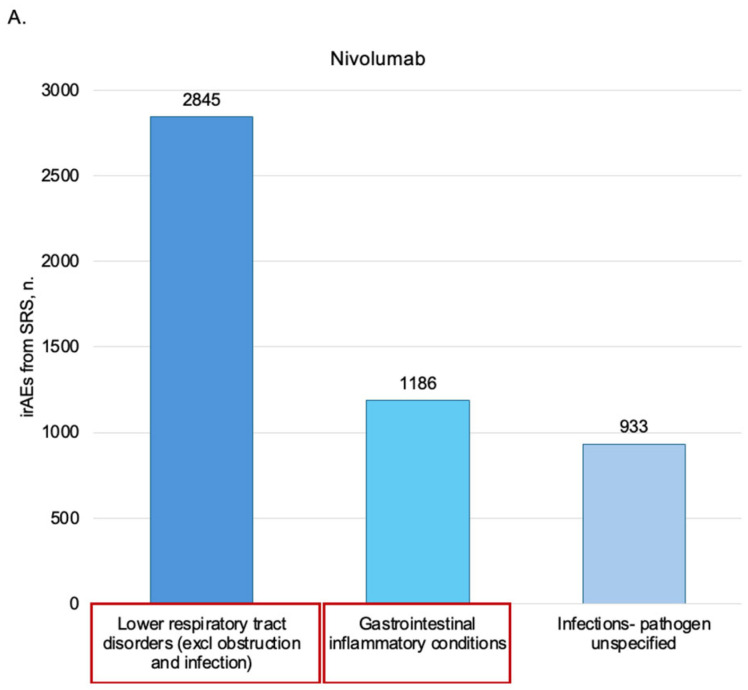

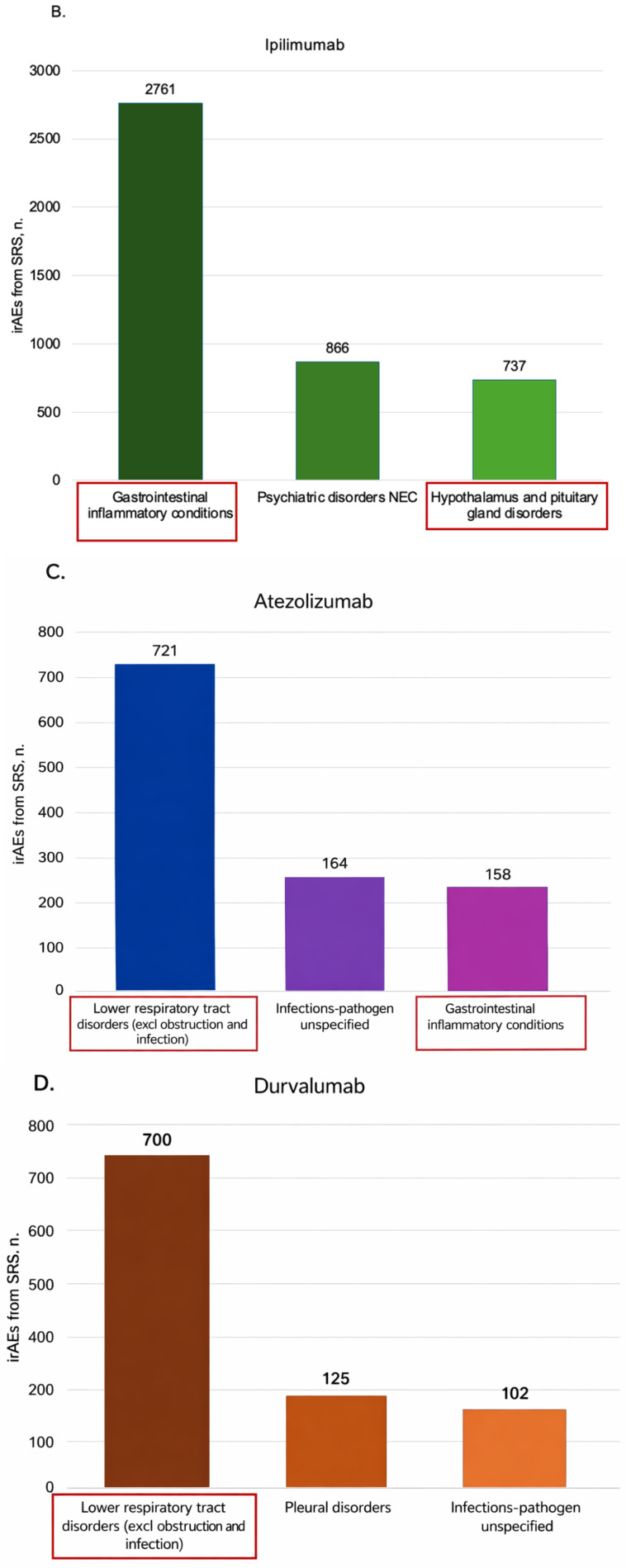

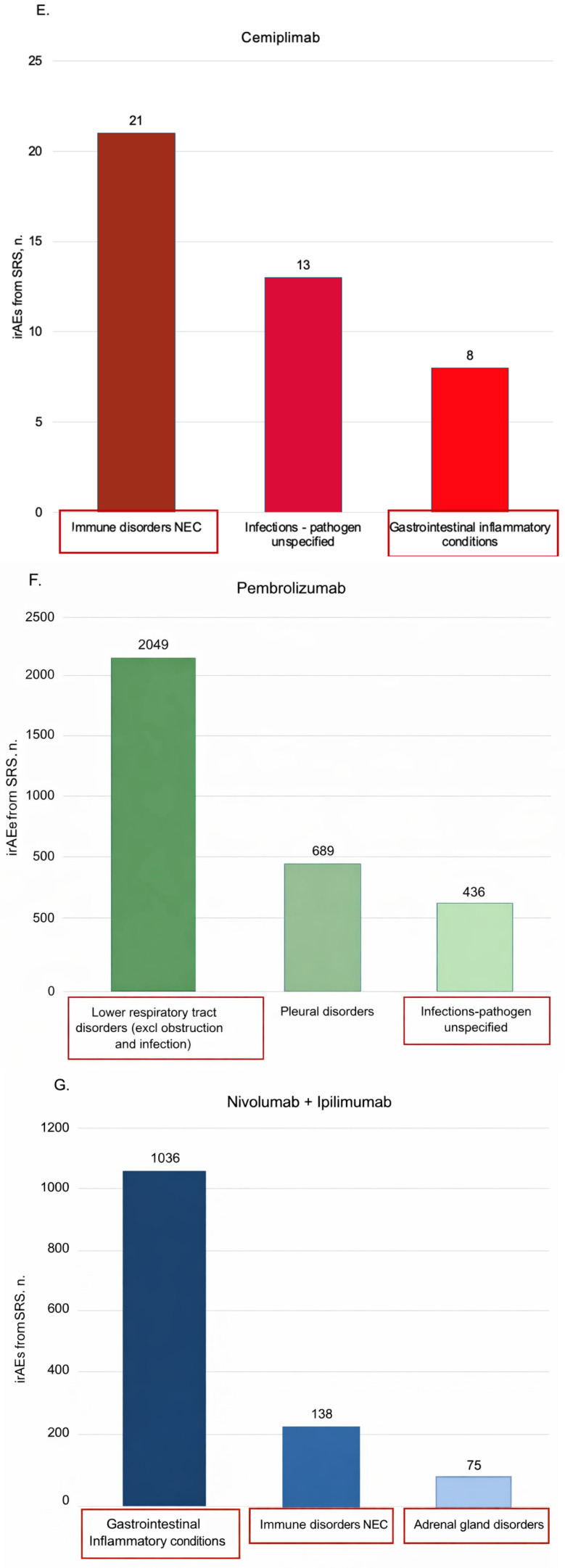

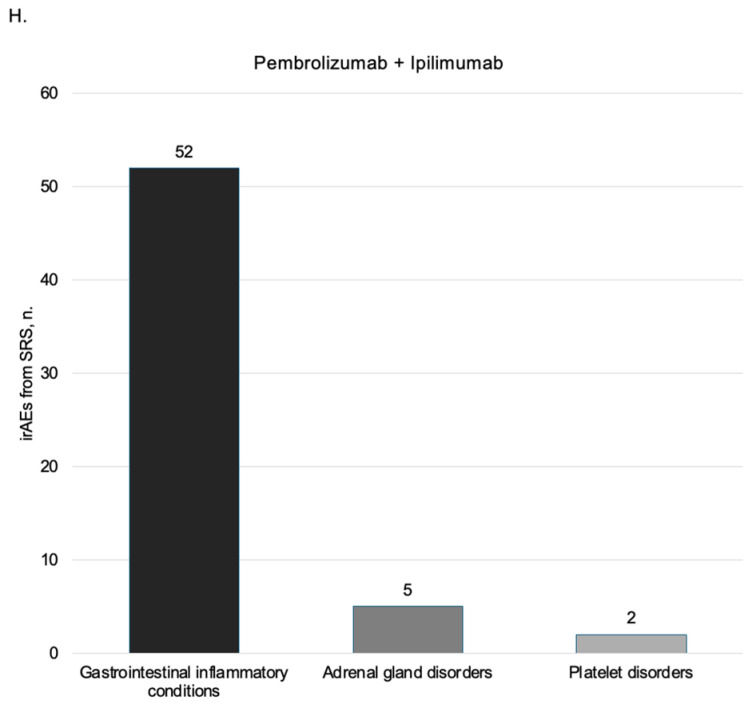
Distribution of the three irAEs most reported in the included studies conducted on SRSs for the drugs of interest (HLGT classification). Highlighted in red are the irAEs reported by both MAs for the drugs of interest. (**A**) Nivolumab. (**B**) Ipilimumab. (**C**) Atezolizumab. (**D**) Durvalumab. (**E**) Cemiplimab. (**F**) Pembrolizumab. (**G**) Nivolumab + Ipilimumab. (**H**) Ipilimumab + Pembrolizumab. SRSs spontaneous reporting systems; HLGT high-level group term; irAEs immune-related adverse events; NEC not elsewhere specified.

Observational studies included in this review concerning SRSs (Table 3 and File Text S1) are conducted principally on FAERS (number = 14 studies, 64%) [34,36,39,42,43,49,50,51,52,53,54,55,56,58] and VigiBase (n = 6, 27%) [32,33,35,38,40,41], and most reports were from America (n = 5, 23%) [32,34,36,38,58] and Japan (n = 3, 14%) [34,56,57]. Disproportionality analysis was performed in 18 (82%) studies [32,33,34,36,38,39,40,41,42,43,50,51,52,53,54,55,57,58]. Among the tumors of interest, lung cancer (subtype unspecified) was the most assessed (n = 11, 50%) [32,35,38,39,40,41,42,50,51,54,56], and pembrolizumab [32,34,35,36,37,38,39,40,41,42,43,49,50,51,52,53,54,56,57,58] and nivolumab [32,34,35,36,37,38,39,41,42,43,49,50,51,52,53,54,55,56,57,58] were the drugs most studied (n = 20, 91%). Patients are mostly male (10,280; 63%), with a mean age of 66 (62–74) years recorded in 7 (32%) studies [32,33,34,36,40,56,58]. The distribution of the studies assessing the time to onset, the duration of irAEs, comorbidities, and concomitant therapies and the distribution of studies assessing the outcome are available in Figure 2, Table 3, and File Text S1. Survival analysis, as well as data on dechallenge and rechallenge, were not reported in any of the studies. Additional information of demographical and clinical characteristics of included studies conducted on SRSs can be found in Table 3 and File Text S1. The irAEs most frequently reported (Table 4) were “Lower respiratory tract disorders (excluding obstruction and infection) for nivolumab, pembrolizumab, atezolizumab, and durvalumab overall, “Gastrointestinal inflammatory conditions” for ipilimumab, nivolumab + ipilimumab and pembrolizumab + ipilimumab, and “Immune disorders NEC” for cemiplimab. Only one study highlighted “Immune disorders NEC” for patients treated with cemiplimab + ipilimumab. It was not possible to retrieve HLGT for pembrolizumab + nivolumab and ipilimumab + pembrolizumab + nivolumab. Additional information on irAEs reported for each drug is provided in Table 4, Figure 3, and File Text S1. It was not possible to retrieve HLGT for pembrolizumab + nivolumab and ipilimumab + pembrolizumab + nivolumab. Table S5 displays HLT of the reported irAEs. Two studies [42,54] reported only System Organ Class (SOC) terms (Table S6), and it was not possible to retrieve HLT/HLGT for these irAEs.

#### 2.2.2. Evidence from Administrative Healthcare Databases

**Figure 4:**
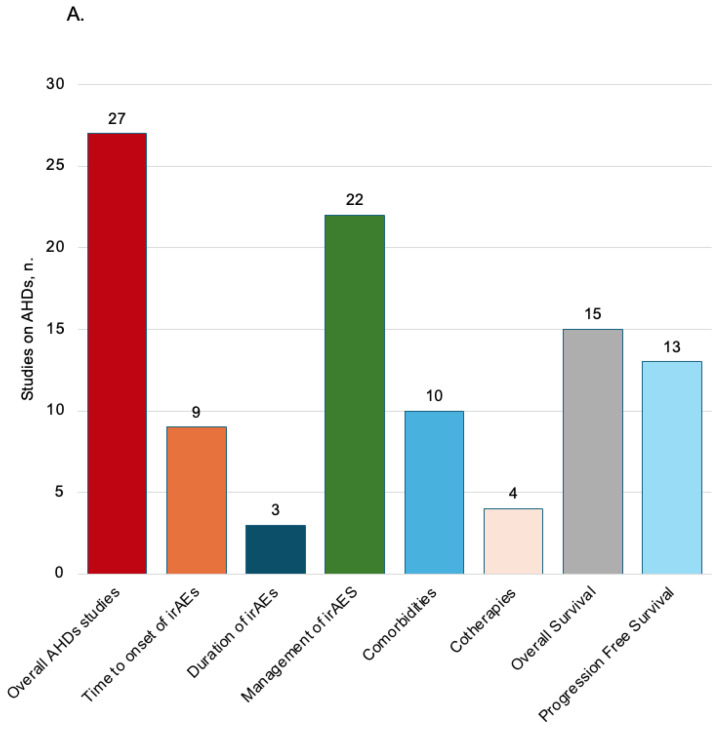

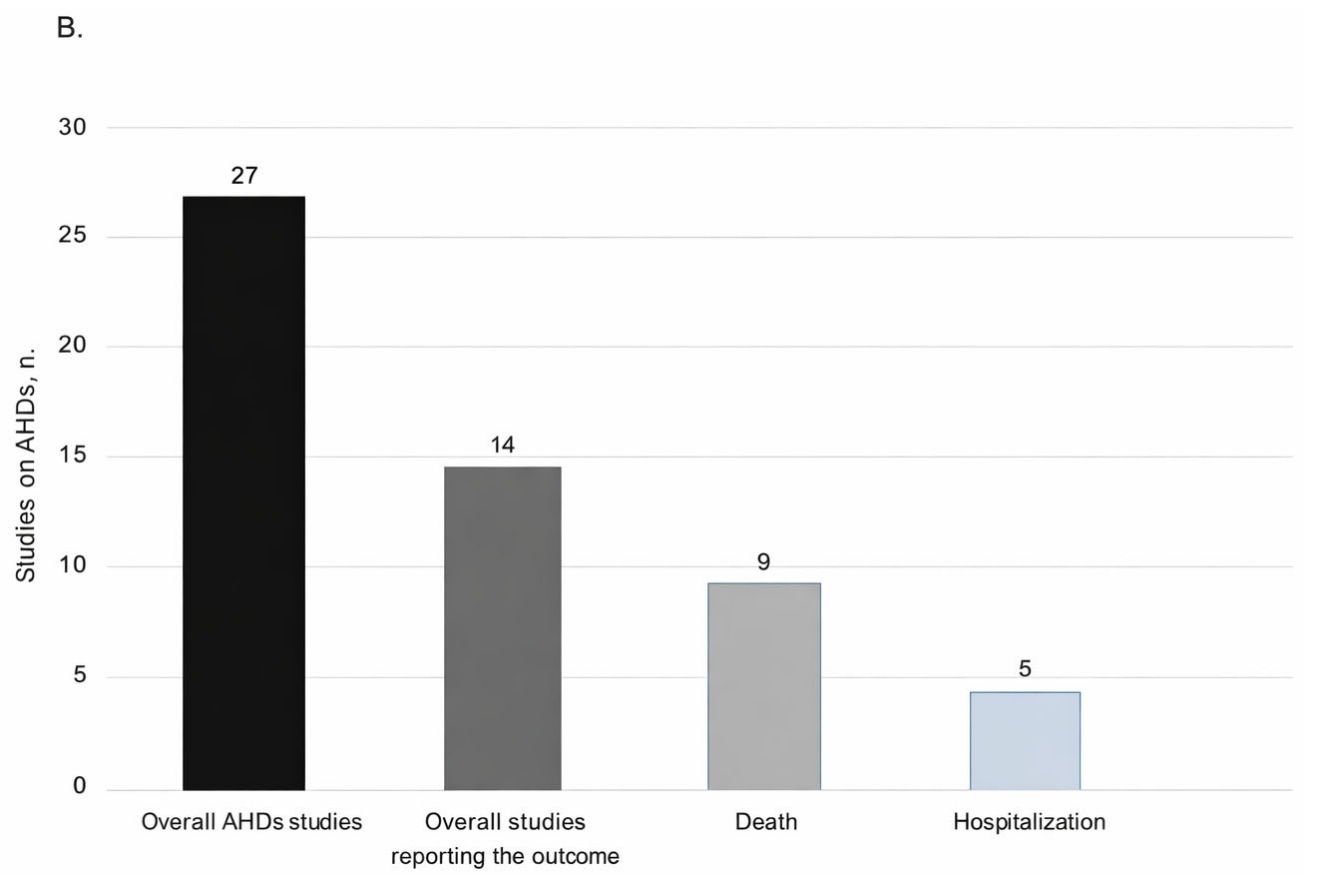
(**A**) Distribution of studies conducted on AHDs assessing the time to onset of irAEs, duration of irAEs, comorbidities, concomitant therapies, and survival analysis. (**B**) Distribution of studies conducted on AHDs assessing the outcome of irAEs. AHDs administrative healthcare databases; irAEs immune-related adverse events.

The observational studies included on AHDs (Table 5 and File Text S2) were conducted mostly in the USA (n = 9; 33%) [11,18,23,25,30,33,44,47,48]. These studies mainly had a retrospective cohort study design (n = 25, 93%) [11,12,13,14,15,16,17,18,19,20,21,22,23,24,25,26,28,29,30,31,44,45,46,47]. The assessment of irAEs was the primary objective (n = 24, 89%) [12,13,14,15,16,17,18,19,20,21,22,23,24,25,26,27,28,29,30,31,33,45,46,47,48]. Electronic medical records are the principal data source utilized (n = 21, 78%) [11,13,15,16,17,18,19,20,21,23,24,25,26,28,30,31,33,44,45,46,48] and the statistical analyses most represented was “descriptive and analytical” (n = 14, 52%) [12,13,15,16,18,20,22,23,25,28,31,44,46,47]. Melanoma (n = 18, 67%) [11,12,14,17,19,20,21,24,25,27,28,29,30,33,45,46,47,48], NSCLC (n = 11, 41%) [13,15,16,17,18,22,24,26,27,28,31], and pembrolizumab (n = 18, 67%) [11,12,13,14,15,16,17,19,20,22,23,27,28,31,44,45,46,47] were the tumors and the drug most assessed. The majority of patients were females 3221 (52%). The mean age was documented in 7 (26%) studies [13,16,20,23,25,28,47], and the corresponding value was 63 (50–70) years old. The distribution of the studies assessing the time to onset, the duration of irAEs, concomitant therapies, comorbidities, and survival analysis and the distribution of studies describing the outcome of irAEs are presented in Figure 4, Table 5, and File Text S2.

**Figure 5:**
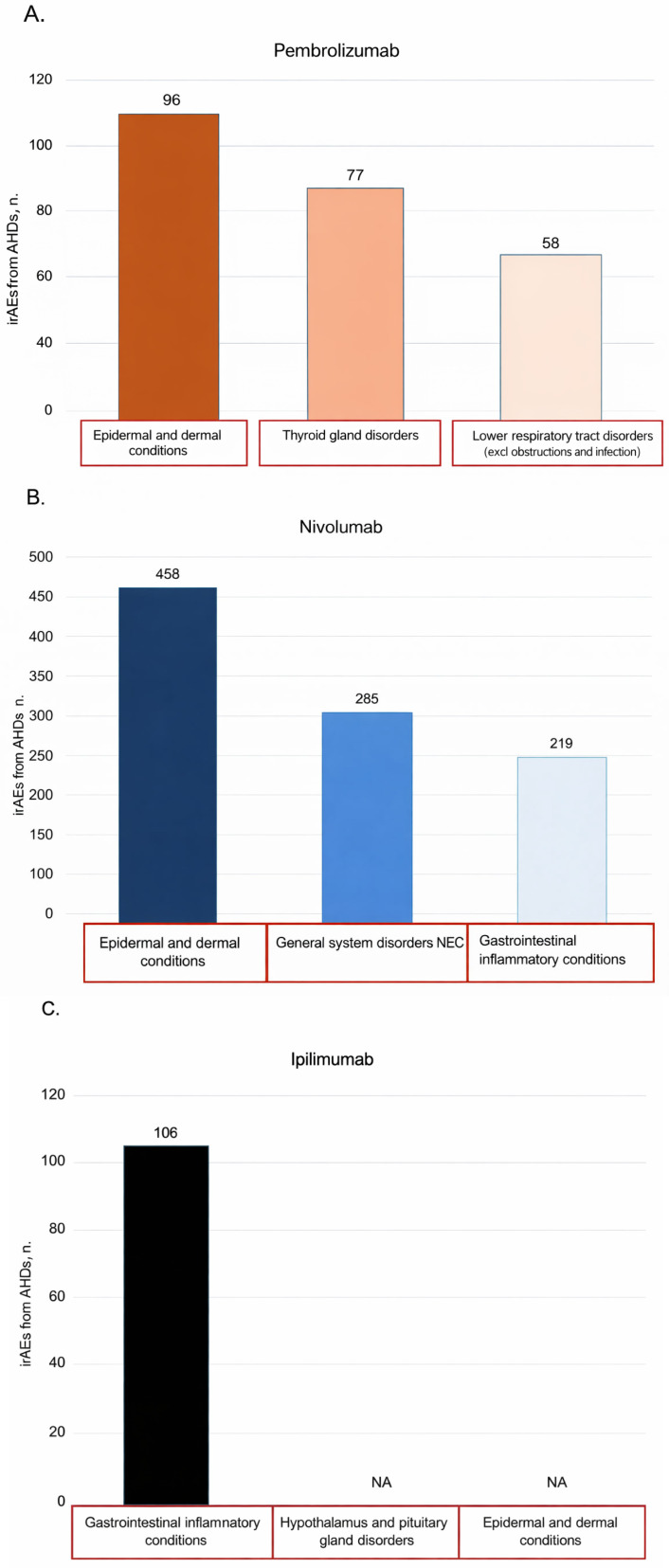

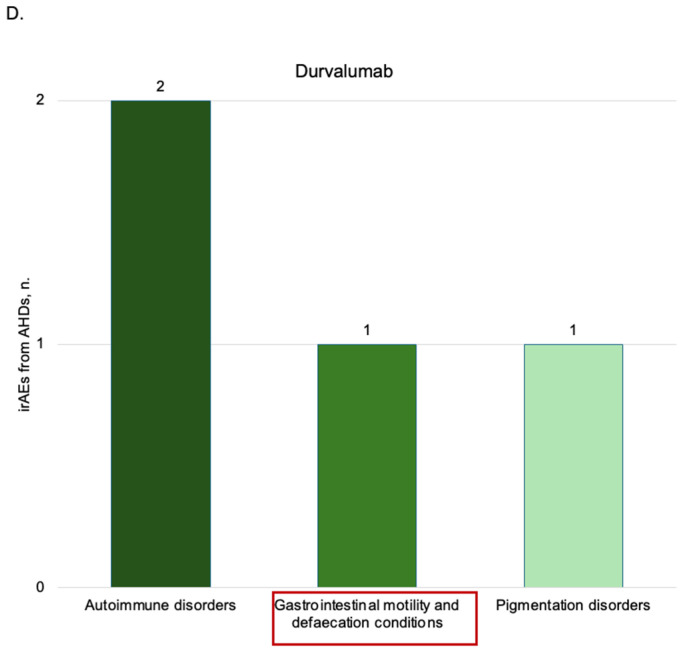
Distribution of the three irAEs most reported in the included studies conducted on AHDs for the drugs of interest (HLGT classification). Highlighted in red are the irAEs reported by both MAs for the drugs of interest. (**A**) Pembrolizumab. (**B**) Nivolumab. (**C**) Ipilimumab. (**D**) Durvalumab. AHDs administrative healthcare databases; HLGT high-level group term; irAEs immune-related adverse events; NEC not elsewhere specified.

The most frequently reported HLGT irAEs of any grade were “epidermal and dermal conditions” for pembrolizumab and nivolumab, “gastrointestinal inflammatory conditions” for ipilimumab, and “autoimmune disorders” for durvalumab. Data on irAEs of any grade for atezolizumab, cemiplimab, and ICI combination therapies were not available. Regarding irAE grade 3 or more, “lower respiratory tract disorders (excluding obstructions and infection)” was the irAE most reported for pembrolizumab and “hepatic and hepatobiliary disorders” for nivolumab. Data on irAEs of any grade for atezolizumab, cemiplimab, and ICI combination therapies and for irAE grade 3 or more associated with atezolizumab, ipilimumab, cemiplimab, durvalumab, and ICI combination therapies were unavailable. Additional information on HLGT irAEs of any grade reported for each drug is displayed in Table 6, Figure 5, and File Text S2 and of irAEs of grade 3 or more in Table S7. HLT irAEs of any grade and grade 3 or more are also reported for each drug in Table S8 and Table S9, respectively.

## 3. Discussion

This scoping review comprehensively explores the landscape of irAEs associated with ICIs as reported by both regulatory authorities, the FDA and EMA, and real-world evidence drawn from AHDs and SRSs. By systematically comparing the labeled irAEs and reviewing observational data, this study provides an integrated understanding of the incidence, clinical features, management, and outcomes of irAEs across key cancer types treated with ICIs, namely lung cancer, melanoma, breast cancer, and colon cancer.

Our analyses of the SPCs from the FDA and EMA reveal substantial concordance in the spectrum of irAEs attributed to ICIs. Commonly reported irAEs encompass a broad range of organ systems including endocrine (thyroid, adrenal, pituitary), cutaneous, GI, hepatic, neurological, and pulmonary adverse drug reactions. This reflects the broad immunomodulatory mechanism common to all ICIs that disrupt immune self-tolerance, precipitating autoimmunity against multiple tissues. Notably, differences between agencies emerge with respect to certain specific irAEs, potentially attributable to variations in post-marketing surveillance, reporting practices, and labeling updates. For instance, FDA annotations include hematopoietic disorders for all ICIs, while the EMA emphasizes “bladder and bladder neck disorders” for all ICIs. These discrepancies underline the importance of continuous pharmacovigilance and the need for harmonization of reporting strategies internationally to enhance risk identification and mitigation [59,60,61].

IrAEs appear to be influenced by the biological target of the ICI (CTLA-4 versus PD-1/PD-L1) as well as by mono versus combination immunotherapy [1]. Ipilimumab (anti-CTLA-4) distinctly exhibits a higher incidence of GI toxicity, consistent with its known mechanism of broader immune activation within the gut-associated lymphoid tissue. Conversely, PD-1/PD-L1 inhibitors such as nivolumab and pembrolizumab demonstrate a relatively wider distribution of irAEs affecting endocrine, pulmonary, and dermatological systems [1,3,6]. Combination therapies (e.g., ipilimumab plus nivolumab) typically yield a compounded safety profile reflecting an additive enhancement of immune activation, resulting in increased incidence and heterogeneity of irAEs [62]. This may influence therapeutic decision-making, particularly in frail populations with pre-existing comorbidities and/or co-therapies or the elderly for which gaps in knowledge still exist.

The inclusion of 49 real-world studies utilizing SRSs (FAERS, VigiBase, EudraVigilance) and AHDs provided complementary perspectives and confirmed the heterogeneity of irAEs affecting multiple organ systems, with respiratory, GI, and dermatological being highly frequent. The incidence and severity of irAEs vary across ICI types, dosing regimens, and tumor indications.

Melanoma and NSCLC remain the most frequently studied indications for ICIs and irAEs.

Endocrine (thyroid, adrenal, pituitary), GI, hepatic, pulmonary, and cutaneous events are most frequently reported for PD-1 (nivolumab, pembrolizumab, cemiplimab) and PD-L1 (atezolizumab, durvalumab) inhibitors. CTLA-4 blockade (ipilimumab) is particularly associated with higher GI adverse events. IrAEs in breast and colon cancer are less commonly reported, highlighting either a lower ICI utilization in this setting or an underreporting phenomenon [63,64].

Ipilimumab has a high risk of “gastrointestinal inflammatory conditions” (reported by both SRSs and AHDs), a medium risk of “psychiatric disorders NEC” (SRSs) and “hypothalamus and pituitary gland disorders” (AHDs), and a low risk of “hypothalamus and pituitary gland disorders” (SRSs) and “epidermal and dermal conditions” (AHDs).

Pembrolizumab has a high risk of “lower respiratory tract disorders (excluding obstruction and infection)” (SRSs) and “epidermal and dermal conditions” (AHDs), a medium risk of “pleural disorders” (SRSs) and “thyroid gland disorders” (AHDs), and a low risk of “infections-pathogen unspecified” (SRSs) and “lower respiratory tract disorders (excluding obstruction and infection)” (AHDs).

Nivolumab has a high risk of “lower respiratory tract disorders (excluding obstruction and infection)” (SRSs) and “epidermal and dermal conditions” (AHDs), a medium risk of “gastrointestinal inflammatory conditions” (SRSs) and “general system disorders NEC” (AHDs), and a low risk of “infections-pathogen unspecified” (SRSs) and “gastrointestinal inflammatory conditions” (AHDs).

Durvalumab has a high risk of “lower respiratory tract disorders (excluding obstruction and infection)” (SRSs) and “autoimmune disorders” (AHDs), a medium risk of “pleural disorders” (SRSs) and “gastrointestinal motility and defecation conditions” (AHDs), and a low risk of “infections-pathogen unspecified” (SRSs) and “pigmentation disorders” (AHDs).

Atezolizumab has a high risk of “lower respiratory tract disorders (excluding obstruction and infection)”, medium risk of “infections-pathogen unspecified”, and low risk of “gastrointestinal inflammatory conditions” (SRSs).

Cemiplimab has a high risk of “immune disorders NEC”, medium risk of “infections-pathogen unspecified”, and low risk of “gastrointestinal inflammatory conditions” (SRSs).

The combination of nivolumab and ipilimumab has a high risk of “gastrointestinal inflammatory conditions”, medium risk of “immune disorders NEC”, and low risk of “adrenal gland disorders” (SRSs), while pembrolizumab combined with ipilimumab has a high risk of “gastrointestinal inflammatory conditions”, medium risk of “adrenal gland disorders”, and low risk of “platelet disorders” (SRSs).

The observed discrepancies in the classification of high-, medium-, and low-risk profiles across individual immune checkpoint inhibitors are largely attributable to differences in data sources and study designs, which inherently introduce heterogeneity and underpin the methodological limitations discussed below.

The mean time to onset reported (from 2 to 420 days) highlights the need for prolonged monitoring. In addition, though the information about dechallenge, rechallenge, duration of irAEs, and survival analyses in SRS studies and about comorbidities and co-therapies in both SRS and AHD studies are lacking, some adverse events have been documented to last for months or years after ICI discontinuation. These types of persistent or chronic irAEs requires ongoing management beyond ICI discontinuation [6,7]. Corticosteroids, therapy discontinuation, and, less commonly, immunosuppressive, or targeted biological agents remain central to irAE management [15,27,37,40]. The balance between managing irAEs and preserving anti-tumor immunity remains a clinical challenge. Notably, mortality [12,15,20,21,24,27,28,32,35,36,39,40,45,47,49,50,51,52,56,58] or permanent sequelae [36,58], while infrequent, were documented, highlighting the need to perform safety monitoring studies.

Only a minority of the included studies reported irAEs using SOC terminology, with 2 of 22 studies conducted on SRSs providing SOC-level data (Table S6). This heterogeneity in reporting terms limits cross-study comparability. Inconsistent use of terminology and variability in the level of classification adopted to describe irAEs impede meaningful synthesis of safety data across studies and restrict the ability to compare organ-specific toxicity profiles. These findings highlight the need for greater harmonization in irAE reporting frameworks in future research.

Higher gastrointestinal inflammatory conditions with anti-CTLA-4 (ipilimumab), align with gut-associated lymphoid tissue activation, while anti-PD-1/PD-L1 agents (e.g., pembrolizumab, nivolumab) show more endocrine, pulmonary (lower respiratory tract disorders), and dermatological events. To enhance actionability, clinicians could prioritize baseline pulmonary function tests and serial imaging for PD-1 users (pneumonitis risk) and frequent stool assessments/early endoscopy for CTLA-4 recipients (colitis risk), extending monitoring 420+ days post-onset based on reported timelines.

SRSs in the review highlight thyroid gland disorders and other endocrine irAEs as frequent, with mean onset up to 180 days, but lack duration data. Prolonged irAEs, persisting months to years post-discontinuation, necessitate lifelong endocrine surveillance like thyroid-stimulating hormone monitoring every 3–6 months, as they demand chronic hormone replacement and increase the healthcare burden. Clinicians should integrate this into follow-up, weighing against ICI rechallenge risks in high-burden patients.

Corticosteroids (1–2 mg/kg/day prednisone equivalent), ICI discontinuation (temporary for grade 2–3, permanent for grade 4), and second-line immunosuppressants (e.g., infliximab for refractory colitis, mycophenolate for hepatitis) form the cornerstone of irAE management, aligning with ASCO (2021 update) and ESMO (2022) guidelines [65,66]. For common irAEs, grade 1 events warrant monitoring and symptomatic care (e.g., topical steroids for rash); grade 2 requires ICI hold and oral corticosteroids; grade 3–4 mandate hospitalization, high-dose IV corticosteroids, and specialist consult (e.g., endocrinology for hypophysitis with MRI/MR pituitary protocol, gastroenterology for colitis with endoscopy). Endocrine irAEs often persist lifelong (e.g., hormone replacement for thyroiditis/hypophysitis, level of evidence: high from prospective cohorts), while dermatologic events resolve with topical agents in most cases (grade 1–2). Pulmonary (pneumonitis) and neurologic irAEs demand urgent bronchoscopy/MRI and avoidance of rechallenge due to fatality risk (ESMO grade of recommendation: I, B).

However, no included studies provided hazard ratios (HRs), confidence intervals, or *p*-values for these associations; analyses relied solely on descriptive medians without multivariable adjustment for confounders like immortal time bias or severity. This gap is explicitly noted as a limitation: among 49 real-world studies, zero reported survival analyses with inferential statistics (e.g., HR < 1 favoring irAEs, *p* < 0.05), precluding robust risk–benefit quantification and highlighting the need for prospective, adjusted analyses [28].

Metanalyses of cohort studies showed that patients who developed irAEs often had better survival and response. A pooled 30-study metanalysis, including 4971 individuals, by Zhou and colleagues demonstrated that endocrine/dermatologic/low grade irAEs are associated with improved overall survival (OS) and progression-free survival (PFS), especially with PD 1/PD L1 monotherapy [67]. Moreover, another 40-study metanalysis, including 8641 participants, found a higher overall response rate (ORR), OS, and PFS in irAE versus non-irAE groups in particular related to skin, endocrine, and gastrointestinal irAEs but not for the liver/lung ones [68]. Some systematic reviews across solid tumors (melanoma, non-small cell lung cancer, renal cell carcinoma gastrointestinal carcinoma, hepatocellular carcinoma) similarly reported a better ORR, PFS, and OS when irAEs occurred, although grade 3–4 irAEs may worsen OS despite a higher ORR [69,70,71,72]. However, the association of irAEs and survival is still controversial and a matter of debate as stated by Amoroso and coworkers who found that the increase in irAE rates between arms correlates poorly with the OS benefit, suggesting that irAE incidence is not a valid surrogate endpoint for efficacy across trials [73]. Finally, little or no clear association for CTLA 4 monotherapy [8,67] has been described and also time-dependent analyses seemed to attenuate or negate the apparent survival advantage, especially for late irAEs [74].

SRSs and AHDs contributed complementary but present intrinsically heterogeneous evidence on irAEs, and their respective strengths and limitations help explain some of the observed pattern differences. In SRSs, lower respiratory tract disorders excluding obstruction and infection emerged as the leading HLGT for nivolumab, pembrolizumab, atezolizumab, and durvalumab, with more than 1100 reports for nivolumab alone (Table 4). This predominance of severe pulmonary events likely reflects a combination of reporting incentives and active surveillance bias: clinicians are encouraged to report serious, life-threatening events, such as pneumonitis, which often prompt hospitalization or treatment discontinuation, and these are frequently targeted by risk-minimization measures and safety communications. In contrast, mild or ambiguous respiratory symptoms (e.g., low-grade dyspnea in patients with underlying lung disease) are less likely to be captured systematically in AHDs if they do not result in coded hospital admissions or specific diagnostic procedures, which may further accentuate the apparent excess of pulmonary irAEs in SRS compared with AHD. Conversely, epidermal and dermal conditions ranked among the three most frequent HLGTs in AHD studies for pembrolizumab, nivolumab, ipilimumab, and durvalumab, whereas SRS contributed relatively fewer skin irAEs overall (Figure 5, Tables S5–S7 versus Table 4). A plausible explanation is that many cutaneous irAEs are low grade, managed in the outpatient setting, and rarely reported spontaneously unless they are severe, atypical, or refractory, leading to under-representation in SRS and relatively higher visibility in AHD, where any coded dermatologic visit, biopsy, or dermatology-related hospital admission can be captured. Under-reporting of mild skin disorders in SRS, together with the focus of AHD on events resulting in health-care utilization, may help explain why cutaneous irAEs appear proportionally more frequent in AHD-based cohorts than in SRS disproportionality analyses.

Finally, the heterogeneity in data sources also affects the overall conclusions regarding the safety profile of ICIs. The predominance of severe pulmonary irAEs in SRS might overestimate their relative weight in the global irAE pattern if interpreted in isolation, whereas AHD findings highlighting frequent endocrine and cutaneous events could under-represent rare but clinically critical toxicities such as myocarditis or neurological irAEs. Integrating both sources and explicitly distinguishing SRS-driven signals from AHD-derived incidence strengthens the interpretation that ICIs induce a multi-organ irAE spectrum.

Several limitations inherent to the datasets analyzed merit discussion.

In this paper, formal survival analyses correlating immune-related adverse events with clinical outcomes (e.g., OS or PFS) could not be quantitatively synthesized, as hazard ratios, confidence intervals, and *p*-values were not consistently reported in the included studies, which often provided only descriptive or median survival estimates.

A further limitation concerns the definition of chronic immune-related adverse events, which was not uniform across the included studies; thresholds for chronicity varied (e.g., persistence beyond 3 or 6 months), preventing the adoption of a single consistent definition and potentially contributing to variability in reported incidence and outcomes.

No reviewed SRS studies validated disproportionality signals against positive controls, such as established ICI-irAE pairs from labels (e.g., pneumonitis with nivolumab). Standard practice requires this to confirm method sensitivity/specificity. Future SRS analyses should incorporate controls like hypothyroidism for PD-1 agents to benchmark signals.

Concomitant therapy and comorbidity data are absent in most SRS studies (only 0–15% report them), precluding adjustments via stratified/multivariable ROR. Cancer progression confounds via competing symptoms (e.g., dyspnea mimicking pneumonitis), unaddressed due to SRS design limits. Clinicians interpreting these signals must overlay AHD data for context.

The analyses based on spontaneous reporting systems (SRSs) should be interpreted as exploratory in nature. Such data sources are inherently affected by underreporting, reporting bias, and unmeasured or unadjusted confounding, and the resulting safety signals cannot be considered causal or formally validated. Consequently, signals identified through SRS analyses should be viewed as hypothesis-generating rather than confirmatory. Validation of clinically relevant signals using complementary data sources, such as administrative healthcare databases (AHD), would be essential to assess their robustness, quantify associated risks, and strengthen the overall interpretability of the findings.

SRSs are subject to underreporting and reporting bias, which may skew incidence estimations and causal assessment [75]. SRSs often contain detailed clinical information reported in a narrative manner, useful for studying the causality assessment, and having the advantage of being available in a short period of time. SRSs are the result of voluntary reporting; therefore, they are influenced by reporting bias and under-reporting. Furthermore, the quality of these reports could also be affected by over-reporting (probably due to the Webber effect or intensive post-marketing surveillance programs), the presence of confounding factors and duplicates, and incompleteness of information reported. Finally, the lack of the denominator does not allow the study of measures of association [76,77].

AHDs often lack clinical details, such as irAE duration, limiting the clinical evaluation. Unlike SRSs, in this case, there is no risk of reporting bias and under-reporting. The limitations of this type of database mainly concern the impossibility of identifying information regarding non-serious AEs due to the tertiary care data collection approach (which generally focuses on serious or complex cases), drugs used for mild conditions or chronic stable states, not covered by the NHS that may still require a doctor’s prescription, paid entirely by patients, drugs purchased privately, use of drugs stored in the family first aid and drugs box, therapies administered during hospitalization that are not recorded at the individual level, general practitioner diagnoses that do not lead to exemptions or hospitalizations, and finally, specialist visits and diagnostic tests performed privately. Clinical information is less detailed in AHDs because data are organized in a structured way for purposes other than the case of interest; therefore, it is difficult to estimate causality; furthermore, information are limited to healthcare services relating to certain geographical areas [78].

Moreover, heterogeneity in study designs, cancer types, and the lacking information about co-therapies and comorbidities, discourage the comparison and meta-analytic synthesis. Only a limited number of studies reported heterogeneous information on dechallenge and rechallenge, highlighting a relevant gap in the available evidence. The absence of standardized and comprehensive reporting limits the ability to draw robust conclusions on both the causal relationship between immune checkpoint inhibitors and immune-related adverse events and the safety of treatment interruption and subsequent re-exposure. 

A further limitation of this review is that formal deduplication across studies was not feasible due to heterogeneity in reported outcomes, immune checkpoint inhibitors and cancer types investigated, and study periods; moreover, potential population overlap could not be assessed, as all included studies reported data exclusively in aggregated form, precluding identification of individual cases.

There is a need for future studies matching multiple data sources to tailor the clinical management of ICI use on the basis not only of ICI and tumor type but also on irAE risk driven by the clinical burden of patients (as comorbidities and co-therapies).

## 4. Methods

We performed this scoping review according to the Preferred Reporting Items for Systematic reviews and Meta-Analyses (PRISMA) guidelines [79] for scoping reviews. The protocol was available online [80], and the checklist was provided in Table S10.

First, we extracted the information contained in the SPC of each drug approved by FDA and EMA, and then, we performed a comprehensive literature review in PubMed to better understand the safety profile in the real world. In this paper, we used the term irAEs to refer both to the expected immune-related adverse drug reactions extracted by the Medicine Agencies and to the immune-related adverse events from AHD studies or the immune-related ADEs from the SRS studies.

EMA and FDA databases were searched for the drugs of interest (ipilimumab, nivolumab, pembrolizumab, cemiplimab, atezolizumab, and durvalumab). Information regarding the date of the first authorization, mechanism of action (target of the drug), therapeutic indication, and irAEs expected were retrieved from EMA and FDA labels [9,10].

Medline and EMBASE databases were searched for relevant articles using a combination of keywords referring to the data sources, tumors, and drugs of interest (see File Text S3). We retrieved and reported information from May 2014 to December 2024. Duplicate records were removed before the screening through title and author screening. Observational studies and target trial emulation studies performed using data collected in AHDs and SRSs and focusing on irAEs associated with the ICIs of interest in NSCLC, melanoma, breast cancer, and colon cancer were included in this review. Full texts were retrieved for inclusion, and if these were not publicly available, the authors were contacted. We excluded articles from authors who did not return any feedback. Studies concerning other drugs, other tumors, other study designs, and other and undefined data sources were excluded. No country restrictions were applied. We reviewed the reference lists of included studies to identify additional relevant studies. We selected studies published on humans and in the English language.

We described irAEs reported by EMA, FDA, and the real-world studies by classifying and reporting these according to the MedDRA. When multiple MedDRA levels were reported across studies, a predefined hierarchical approach was applied to ensure consistency. Specifically, HLGTs were prioritized when available. If HLGTs were not reported, HLTs were used; when neither HLGTs nor HLTs were available, events were classified at the SOC level. This hierarchical selection was applied uniformly across all included studies to maximize comparability while preserving the highest possible level of clinical detail. All the information extracted regarding irAEs and survival analysis concern only the data available related to the drugs of interest. We described the baseline and clinical characteristics and the findings of real-world studies by retrieving the information about distribution n (%) or time value, and we calculated the mean value whenever it was possible. The mean value (min-max) was calculated for age, time to onset, and duration of irAE and OS/PFS when possible, otherwise the value was reported according to the original study.

This review included only retrospective studies based on previously collected data. Ethical approval was obtained, when required, at the time of conduct of the individual primary studies. For data sources based on spontaneous reporting systems (e.g., VigiBase), analyses were performed on de-identified, aggregated data made publicly available for research purposes; therefore, no additional ethical approval or informed consent was required for the present review.

Although the study period spans from 2014 to 2024, some included publications report data that extend into subsequent years or derive from prospectively registered sources. However, only results based on retrospectively collected data were extracted and analyzed in the present review.

## 5. Conclusions

This scoping review provides a detailed synthesis of the irAEs associated with ICIs, integrating regulatory and real-world evidence. The findings emphasize the complexity of irAEs, their multi-organ involvement, and the critical importance of comprehensive monitoring and individualized clinical management to maximize therapeutic benefit while mitigating harm.

## Figures and Tables

**Table 1:** Therapeutic indications and safety profile of ICIs of interest approved by EMA and FDA: information common to both agencies.

Immune-Related Adverse Events Expected (HLGT)	Drugs Involved	Therapeutic Indications
Adrenal gland disorders	Ipilimumab, ipilimumab + nivolumab, nivolumab, pembrolizumab, cemiplimab, atezolizumab, durvalumab	Melanoma, renal cell carcinoma, non-small cell lung cancer, malignant pleural mesothelioma, mismatch repair deficient or microsatellite instability-high colorectal cancer, esophageal squamous cell carcinoma, classical Hodgkin lymphoma, squamous cell cancer of the head and neck, urothelial carcinoma, gastric, gastro-esophageal junction or esophageal adenocarcinoma, microsatellite instability-high or mismatch repair deficient cancers, triple-negative breast cancer, endometrial carcinoma, cervical cancer, biliary tract carcinoma, small cell lung cancer, hepatocellular carcinoma, cutaneous squamous cell carcinoma, basal cell carcinoma
Central nervous system infections and inflammations		
Epidermal and dermal conditions		
Exocrine pancreas conditions		
Gastrointestinal inflammatory conditions		
Gastrointestinal motility and defecation conditions		
Gastrointestinal signs and symptoms		
Glucose metabolism disorders (including diabetes mellitus)		
Hepatic and hepatobiliary disorders		
Hypothalamus and pituitary gland disorders		
Immune disorders NEC		
Joint symptoms		
Lower respiratory tract disorders (excluding obstructions and infection)		
Muscle disorders		
Myocardial disorders		
Nephropathies		
Neuromuscular disorders		
Ocular infections, irritations and inflammations		
Peripheral neuropathies		
Procedural related injuries and complications NEC		
Thyroid gland disorders		
Vascular infections and inflammations		
Endocrine disorders of gonad function	Ipilimumab	Melanoma
Pericardial disorders	Ipilimumab, ipilimumab + nivolumab, nivolumab, pembrolizumab, cemiplimab, atezolizumab	Melanoma, renal cell carcinoma, NSCLC, malignant pleural mesothelioma, mismatch repair deficient or microsatellite instability-high colorectal cancer, esophageal squamous cell carcinoma, classical Hodgkin lymphoma, squamous cell cancer of the head and neck, urothelial carcinoma, gastric, gastro-esophageal junction or esophageal adenocarcinoma, head and neck squamous cell carcinoma, microsatellite instability-high or mismatch repair deficient cancers, triple-negative breast cancer, endometrial carcinoma, cervical cancer, biliary tract carcinoma, cutaneous squamous cell carcinoma, basal cell carcinoma, SCLC, hepatocellular carcinoma
Demyelinating disorders	Ipilimumab, ipilimumab + nivolumab, nivolumab,	Melanoma, renal cell carcinoma, NSCLC, malignant pleural mesothelioma, mismatch repair deficient or microsatellite instability-high colorectal cancer, esophageal squamous cell carcinoma, classical Hodgkin lymphoma, squamous cell cancer of the head and neck, urothelial carcinoma, gastric, gastro-esophageal junction or esophageal adenocarcinoma
Gastrointestinal investigation	Ipilimumab, ipilimumab + nivolumab, nivolumab, pembrolizumab, atezolizumab	Melanoma, renal cell carcinoma, NSCLC, malignant pleural mesothelioma, mismatch repair deficient or microsatellite instability-high colorectal cancer, esophageal squamous cell carcinoma, classical Hodgkin lymphoma, squamous cell cancer of the head and neck, urothelial carcinoma, gastric, gastro-esophageal junction or esophageal adenocarcinoma, head and neck squamous cell carcinoma, microsatellite instability-high or mismatch repair deficient cancers, triple-negative breast cancer, endometrial carcinoma, cervical cancer, biliary tract carcinoma, SCLC, hepatocellular carcinoma
Ocular structural change, deposit and degeneration NEC	Ipilimumab	Melanoma
Renal disorders (excluding nephropathies)	Ipilimumab, ipilimumab + nivolumab, nivolumab, pembrolizumab, cemiplimab	Melanoma, renal cell carcinoma, NSCLC, malignant pleural mesothelioma, mismatch repair deficient or microsatellite instability-high colorectal cancer, esophageal squamous cell carcinoma, classical Hodgkin lymphoma, squamous cell cancer of the head and neck, urothelial carcinoma, gastric, gastro-esophageal junction or esophageal adenocarcinoma, head and neck squamous cell carcinoma, microsatellite instability-high or mismatch repair deficient cancers, triple-negative breast cancer, endometrial carcinoma, cervical cancer, biliary tract carcinoma, cutaneous squamous cell carcinoma, basal cell carcinoma
Connective tissue disorders (excluding nephropathies)	Ipilimumab, ipilimumab + nivolumab, nivolumab, pembrolizumab, cemiplimab, atezolizumab	Melanoma, renal cell carcinoma, NSCLC, malignant pleural mesothelioma, mismatch repair deficient or microsatellite instability-high colorectal cancer, esophageal squamous cell carcinoma, classical Hodgkin lymphoma, squamous cell cancer of the head and neck, urothelial carcinoma, gastric, gastro-esophageal junction or esophageal adenocarcinoma, head and neck squamous cell carcinoma, microsatellite instability-high or mismatch repair deficient cancers, triple-negative breast cancer, endometrial carcinoma, cervical cancer, biliary tract carcinoma, cutaneous squamous cell carcinoma, basal cell carcinoma, SCLC, hepatocellular carcinoma
General system disorders NEC	Ipilimumab, nivolumab	Melanoma, renal cell carcinoma, NSCLC, classical Hodgkin lymphoma, squamous cell cancer of the head and neck, urothelial carcinoma, esophageal squamous cell carcinoma, gastric, gastro-esophageal junction or esophageal adenocarcinoma
Joint disorders	Ipilimumab, ipilimumab + nivolumab, nivolumab, pembrolizumab, cemiplimab, durvalumab	Melanoma, renal cell carcinoma, NSCLC, malignant pleural mesothelioma, mismatch repair deficient or microsatellite instability-high colorectal cancer, esophageal squamous cell carcinoma, classical Hodgkin lymphoma, squamous cell cancer of the head and neck, urothelial carcinoma, gastric, gastro-esophageal junction or esophageal adenocarcinoma, head and neck squamous cell carcinoma, microsatellite instability-high or mismatch repair deficient cancers, triple-negative breast cancer, endometrial carcinoma, cervical cancer, biliary tract carcinoma, cutaneous squamous cell carcinoma, basal cell carcinoma, SCLC, hepatocellular carcinoma
Infections-pathogen unspecified	Ipilimumab, pembrolizumab	Melanoma, NSCLC, classical Hodgkin lymphoma, urothelial carcinoma, head and neck squamous cell carcinoma, renal cell carcinoma, microsatellite instability-high or mismatch repair deficient cancers, microsatellite instability high or mismatch repair deficient colorectal, esophageal carcinoma, triple-negative breast cancer, endometrial carcinoma, cervical cancer, gastric or gastro-esophageal junction adenocarcinoma, biliary tract carcinoma
Diabetic complications	Ipilimumab, ipilimumab + nivolumab, nivolumab, pembrolizumab, cemiplimab, atezolizumab, durvalumab	Melanoma, renal cell carcinoma, NSCLC, malignant pleural mesothelioma, mismatch repair deficient or microsatellite instability-high colorectal cancer, esophageal squamous cell carcinoma, classical Hodgkin lymphoma, squamous cell cancer of the head and neck, urothelial carcinoma, gastric, gastro-esophageal junction or esophageal adenocarcinoma, head and neck squamous cell carcinoma, microsatellite instability-high or mismatch repair deficient cancers, triple-negative breast cancer, endometrial carcinoma, cervical cancer, biliary tract carcinoma, cutaneous squamous cell carcinoma, basal cell carcinoma, SCLC, hepatocellular carcinoma
Gastrointestinal hemorrhages NEC	Ipilimumab, nivolumab, atezolizumab, durvalumab	Melanoma, renal cell carcinoma, NSCLC, classical Hodgkin lymphoma, squamous cell cancer of the head and neck, urothelial carcinoma, esophageal squamous cell carcinoma, gastric, gastro-esophageal junction or esophageal adenocarcinoma, SCLC, hepatocellular carcinoma, biliary tract cancer, endometrial cancer
Vascular disorders NEC	Ipilimumab	Melanoma
Ocular infections, irritations and inflammations	Ipilimumab	Melanoma
Hearing disorders	Ipilimumab	Melanoma
White blood cell disorders	Ipilimumab, ipilimumab + nivolumab, nivolumab	Melanoma, renal cell carcinoma, NSCLC, malignant pleural mesothelioma, mismatch repair deficient or microsatellite instability-high colorectal cancer, esophageal squamous cell carcinoma, classical Hodgkin lymphoma, squamous cell cancer of the head and neck, urothelial carcinoma, gastric, gastro-esophageal junction or esophageal adenocarcinoma
Gastrointestinal ulcer and perforation	Ipilimumab + nivolumab, pembrolizumab, durvalumab	Melanoma, renal cell carcinoma, NSCLC, malignant pleural mesothelioma, mismatch repair deficient or microsatellite instability-high colorectal cancer, esophageal squamous cell carcinoma, classical Hodgkin lymphoma, urothelial carcinoma, head and neck squamous cell carcinoma, microsatellite instability-high or mismatch repair deficient cancers, microsatellite instability high or mismatch repair deficient colorectal, triple-negative breast cancer, endometrial carcinoma, cervical cancer, gastric or gastro-esophageal junction adenocarcinoma, biliary tract carcinoma, SCLC, hepatocellular carcinoma
Hemolysis and related conditions	Ipilimumab + nivolumab, nivolumab, durvalumab	Melanoma, renal cell carcinoma, NSCLC, malignant pleural mesothelioma, mismatch repair deficient or microsatellite instability-high colorectal cancer, classical Hodgkin lymphoma, squamous cell cancer of the head and neck, urothelial carcinoma, esophageal squamous cell carcinoma, gastric, gastro-esophageal junction or esophageal adenocarcinoma, biliary tract cancer, SCLC, hepatocellular carcinoma, endometrial cancer
Hepatobiliary investigations	Ipilimumab + nivolumab, nivolumab, pembrolizumab, cemiplimab, atezolizumab, durvalumab	Melanoma, renal cell carcinoma, NSCLC, malignant pleural mesothelioma, mismatch repair deficient or microsatellite instability-high colorectal cancer, esophageal squamous cell carcinoma, classical Hodgkin lymphoma, squamous cell cancer of the head and neck, urothelial carcinoma, gastric, gastro-esophageal junction or esophageal adenocarcinoma, head and neck squamous cell carcinoma, microsatellite instability-high or mismatch repair deficient cancers, triple-negative breast cancer, endometrial carcinoma, cervical cancer, biliary tract carcinoma, cutaneous squamous cell carcinoma, basal cell carcinoma, SCLC, hepatocellular carcinoma
Oral soft tissue conditions	Ipilimumab + nivolumab, nivolumab, pembrolizumab, cemiplimab, atezolizumab, durvalumab	Melanoma, renal cell carcinoma, NSCLC, malignant pleural mesothelioma, mismatch repair deficient or microsatellite instability-high colorectal cancer, esophageal squamous cell carcinoma, classical Hodgkin lymphoma, squamous cell cancer of the head and neck, urothelial carcinoma, gastric, gastro-esophageal junction or esophageal adenocarcinoma, head and neck squamous cell carcinoma, microsatellite instability-high or mismatch repair deficient cancers, triple-negative breast cancer, endometrial carcinoma, cervical cancer, biliary tract carcinoma, cutaneous squamous cell carcinoma, basal cell carcinoma, SCLC, hepatocellular carcinoma
Parathyroid gland disorders	Ipilimumab + nivolumab, nivolumab, pembrolizumab	Melanoma, renal cell carcinoma, NSCLC, malignant pleural mesothelioma, mismatch repair deficient or microsatellite instability-high colorectal cancer, esophageal squamous cell carcinoma, classical Hodgkin lymphoma, squamous cell cancer of the head and neck, urothelial carcinoma, gastric, gastro-esophageal junction or esophageal adenocarcinoma, head and neck squamous cell carcinoma, microsatellite instability-high or mismatch repair deficient cancers, triple-negative breast cancer, endometrial carcinoma, cervical cancer, biliary tract carcinoma
Vision disorders	Ipilimumab + nivolumab	Melanoma, renal cell carcinoma, NSCLC, malignant pleural mesothelioma, mismatch repair deficient or microsatellite instability-high colorectal cancer, esophageal squamous cell carcinoma
Bile duct disorders	Pembrolizumab, durvalumab	Melanoma, NSCLC, classical Hodgkin lymphoma, urothelial carcinoma, head and neck squamous cell carcinoma, renal cell carcinoma, microsatellite instability-high or mismatch repair deficient cancers, microsatellite instability high or mismatch repair deficient colorectal, esophageal carcinoma, triple-negative breast cancer, endometrial carcinoma, cervical cancer, gastric or gastro-esophageal junction adenocarcinoma, biliary tract carcinoma, SCLC, hepatocellular carcinoma
Anemias nonhemolytic and marrow depression	Durvalumab	NSCLC, biliary tract cancer, SCLC, hepatocellular carcinoma, endometrial cancer
Injuries by physical agents	Durvalumab	NSCLC, biliary tract cancer, SCLC, hepatocellular carcinoma, endometrial cancer

ICIs immune checkpoint inhibitors, HLGT high-level group terms, EMA European Medicines Agency, FDA Food and Drug Administration, NSCLC non-small cell lung cancer, SCLC small cell lung cancer, NEC not elsewhere classified.

**Table 2:** Safety profile of ICIs of interest approved by EMA and FDA: information specific to each agency.

Immune-Related Adverse Events Expected (HLGT)	Drugs Involved	
	EMA	FDA
Bladder and bladder neck disorders (excluding calculi)	Ipilimumab, ipilimumab + nivolumab, nivolumab, pembrolizumab, atezolizumab, durvalumab	-
Diabetic complications	Ipilimumab	-
Gastrointestinal ulcer and perforation	Ipilimumab, atezolizumab	-
Hemolysis and related conditions	Ipilimumab	Atezolizumab
Hepatobiliary investigations	Ipilimumab	-
Malabsorption conditions	Ipilimumab, ipilimumab + nivolumab, nivolumab, pembrolizumab, atezolizumab, durvalumab	-
Ocular injuries	Ipilimumab	Ipilimumab + nivolumab, nivolumab, pembrolizumab, atezolizumab
Oral soft tissue conditions	Ipilimumab	
Parathyroid gland disorders	Ipilimumab	Atezolizumab, durvalumab
Anemias nonhemolytic and marrow depression	-	Ipilimumab, ipilimumab + nivolumab, nivolumab
Hematopoietic neoplasms (excluding leukemias and lymphomas)	-	Ipilimumab, nivolumab, pembrolizumab, atezolizumab, durvalumab
Movement disorders (including parkinsonism)	-	Ipilimumab
Pericardial disorders	-	Ipilimumab
Skin vascular abnormalities	-	Ipilimumab
Vision disorders	Nivolumab, pembrolizumab, atezolizumab, durvalumab	Ipilimumab
Cranial nerve disorders (excluding neoplasms)	Ipilimumab + nivolumab, nivolumab, atezolizumab	-
Infections-pathogen unspecified	Ipilimumab + nivolumab, nivolumab, atezolizumab	Durvalumab
Ocular infections, irritations and inflammations	Ipilimumab + nivolumab	-
Anemias nonhemolytic and marrow depression	-	Ipilimumab, ipilimumab + nivolumab, nivolumab, pembrolizumab, atezolizumab
General system disorders NEC	-	Ipilimumab + nivolumab, pembrolizumab, atezolizumab, durvalumab
Ocular structural change, deposit and degeneration NEC	-	Ipilimumab + nivolumab, nivolumab, pembrolizumab, atezolizumab, durvalumab
Bile duct disorders	-	Nivolumab
Gastrointestinal ulcer and perforation	-	Nivolumab
White blood cell disorders	Pembrolizumab	Pembrolizumab
Demyelinating disorders	-	Pembrolizumab, atezolizumab, durvalumab
Injuries by physical agents	Atezolizumab	
Joint disorders	-	Atezolizumab
Renal disorders (excluding nephropathies)	-	Atezolizumab, durvalumab
Connective tissue disorders (excluding nephropathies)	-	Durvalumab

ICIs immune checkpoint inhibitors, EMA European Medicines Agency, FDA Food and Drug Administration, HLGT high-level group terms, NEC not elsewhere classified.

**Table 3:** Characteristics of included studies conducted on SRSs ^ç^.

Characteristics	N (%)
*Studies included*	22 (100)
*Data source*	
FAERS	14 (64)
VigiBase	6 (27)
EudraVigilance	1 (5)
JADER	1 (5)
*Continent*	
Europe	10 (45)
Asia	7 (32)
America	6 (27)
Africa	2 (9)
Oceania	2 (9)
Other not specified	1 (5)
*Type of the analysis used in the studies*	
Disproportionality	18 (82)
Descriptive	4 (18)
*Type of tumors reported in the studies*	
Lung cancer (subtype unspecified)	11 (50)
Melanoma	10 (46)
GI (included duodenal, colon, gastric)	10 (46)
Kidney cancer	10 (46)
Genitourinary cancer	9 (41)
Breast cancer	7 (32)
Liver cancer	7 (32)
Prostate cancer	6 (27)
Nervous system cancer	5 (23)
Head and neck cancer	5 (23)
Skin cancer	5 (23)
NSCLC	4 (18)
Pleural cancer	3 (14)
Hematopoietic and lymphoid tissues cancer	3 (14)
Pancreatic cancer	2 (9)
Cholangiocarcinoma	2 (9)
Myeloma	2 (9)
Leukemia	2 (9)
Hodgkin	2 (9)
Non-Hodgkin	2 (9)
Endocrine cancer	2 (9)
Mesothelioma	1 (5)
Neuroendocrine cancer	1 (5)
Thyroid cancer	1 (5)
SCLC	1 (5)
Thymus, pleural and heart cancer	1 (5)
Heart cancer	1 (5)
Corneal cancer	1 (5)
Urothelial carcinoma	1 (5)
Other cancers not specified	6 (27)
*Drugs of our interest reported in the studies*	
Pembrolizumab (overall)	20 (91)
Pembrolizumab + T	2
Nivolumab (overall)	20 (91)
Nivolumab + T	2
Ipilimumab (overall)	15 (68)
Ipilimumab + T	0
Atezolizumab (overall)	16 (73)
Atezolizumab + T	1
Durvalumab (overall)	14 (64)
Durvalumab + T °	1
Cemiplimab (overall)	9 (41)
Cemiplimab + T	0
Combination ICI therapies specified	15 (68)
Nivolumab + ipilimumab	7 (32)
Pembrolizumab + ipilimumab	5 (23)
Pembrolizumab + nivolumab	1 (5)
Cemiplimab + ipilimumab	1 (5)
Ipilimumab + pembrolizumab + nivolumab	1 (5)
Other therapies not of our interest ^§^	14 (64)
Other therapies not specified *	4 (18)
*Reports analyzed in the studies concerning the drugs of interest (overall)*	321.610
*Gender*	
Overall reporting gender	16.406 (100)
Male	10.280 (63)
Female	6.126 (37)
*Age*	
Studies reporting the mean value	7 (32)
Mean value (min-max)	66 (62–74)
Studies reporting the median value	1 (5)
*Studies reporting the time to onset of irAEs*	4 (18)
Mean value (min-max), days	92 (45–180)
Studies reporting correspondence overall time to onset-specific irAE **	2
Mean value (min-max), days	62 (45–92)
Studies reporting correspondence time to onset-drug-specific irAE ***	1
Mean value (min-max), days	101 (68–134)
Studies reporting correspondence time to onset-drug ****	1
Mean value (min-max), days	113 (69–180)
*Studies reporting the duration of irAEs*	2 (9)
Mean value reported overall (SD), days	26 (46)
Median value reported overall (range), days	56 (21–112)
Studies reporting correspondence duration of irAE-drug *****	1 (50)
Studies reporting correspondence duration of irAE-irAE ******	1 (50)
*Studies reporting the management of irAEs*	3 (14)
Discontinuation	2 (67)
Systemic corticosteroids	1 (33)
Topical steroids	1 (33)
Temporarily interrupted	1 (33)
Phototherapy	1 (33)
Systemic treatment	1 (33)
Acitretin	1 (33)
Etanercept	1 (33)
Apremilast	1 (33)
Traditional and newer systemic antipsoriatic agents	1 (33)
*Studies reporting comorbidities*	3 (14)
Patients reported with comorbidities, n	164 (0)
Studies reporting the specific comorbidity	3 (100)
Diabetes mellitus	1 (33)
Pre-existing psoriasis	1 (33)
Multiple sclerosis	1 (33)
*Studies reporting concomitant therapies*	3 (14)
Patients reported with at least one co-therapy, n	554 (0)
Studies reporting the specific therapy	2 (67)
Multiple sclerosis treatment	1 (33)
Atorvastatin	1 (33)
*Studies reporting the outcome of irAE (overall)*	12 (55)
Death	11 (92)
HO	5 (42)
Disability	3 (25)
Life-threatening	3 (25)
Recovered/resolved	3 (25)
Required intervention to prevent permanent impairment/damage	2 (17)
Not recovered/not resolved	2 (17)
Recovering/resolving	1 (8)
Resolved with sequelae	1 (8)
Other not specified	5 (42)
*Studies reporting the survival analysis*	0
*Studies reporting dechallenge and rechallenge*	0

^Ç^ All the information reported in this table concerns only the data available and undoubtedly related to the drugs of interest. ° Durvalumab + T: Durvalumab + tremelimumab (reported in 2 studies). ^§^ Other drugs not of our interest: avelumab (reported in 12 studies), tremelimumab (reported in 1 study), dostarlimab (reported in 1 study). * Other drugs not specified: “PD-1 inhibitors”, “anti-PD-(L)1”, “anti-PD-1+ anti-CTLA4”. ** Garcia et al. 2019 [49] reported the time to symptom onset of multiple sclerosis relapse. Huang et al. 2020 [32] reported the time to onset for each of the following irAE: enterocolitis, gastrointestinal perforation and intestinal obstruction. *** Hu et al. (2020) [36] reported the mean value of the time to onset for colitis for nivolumab, pembrolizumab, atezolizumab, durvalumab, and ipilimumab + nivolumab each. **** Ali et al. (2017) [58] reported the time to onset for ipilimumab, pembrolizumab and nivolumab each. ***** Ali et al. (2017) [58] reported the duration of irAE for ipilimumab, pembrolizumab, nivolumab, and overall. ****** Garcia et al. (2019) [49] reported the duration of irAE for multiple sclerosis relapse. SRSs Spontaneous Reporting Systems; FAERS Food and Drug Administration Reporting System; JADER Japanese Adverse Drug Event Report; GI gastrointestinal; NSCLC non-small cell lung cancer; SCLC small cell lung cancer; SD standard deviation; OS overall survival; PFS progression free survival; ICI immune checkpoint inhibitors; irAE immune-related adverse events; T therapy; HO hospitalization.

**Table 4:** irAEs reported for each drug in the included studies conducted on SRSs (HLGT classification).

Drug	Tumor	Number of Studies Reporting the Drug for the Tumor and irAEs	Number of irAEs	HLGT
Nivolumab	Overall	14	2845	Lower respiratory tract disorders (excluding obstruction and infection)
			1186	Gastrointestinal inflammatory conditions
			933	Infections-pathogen unspecified
			807	Peripheral neuropathies
			802	Pleural disorders
			507	Hypothalamus and pituitary gland disorders
			324	Muscle disorders
			256	Appetite and general nutritional disorders
			220	Glucose metabolism disorders (including diabetes mellitus)
			200	Adrenal gland disorders
			191	Neuromuscular disorders
			186	Peritoneal and retroperitoneal conditions
			167	Immune disorders NEC
			156	Central nervous system vascular disorders
			122	Respiratory disorders NEC
			115	Platelet disorders
			109	Bile duct disorders
			93	Encephalopathies
			83	Gastrointestinal stenosis and obstruction
			67	Leukemias
			64	Ocular neuromuscular disorders
			58	Hepatobiliary investigations
			49	Vascular infections and inflammations
			48	Demyelinating disorders
			46	Epidermal and dermal conditions
			42	Hepatic and hepatobiliary disorders
			42	Thyroid gland disorders
			38	Gastrointestinal motility and defecation conditions
			29	Neoplastic and ectopic endocrinopathies
			17	Procedural related injuries and complications NEC
			14	Enzyme investigations NEC
			10	Renal disorders (excluding nephropathies)
			10	Ocular infections, irritations and inflammations
			9	Nephropathies
			9	Adrenal gland disorders/hepatic and hepatobiliary disorders/diabetic complications
	Lung cancer (subtype unspecified)	1	312	Lower respiratory tract disorders (excluding obstruction and infection)
	Melanoma	1	58	Hepatobiliary investigations
			42	Thyroid gland disorders
			38	Gastrointestinal motility and defecation conditions
			31	Lower respiratory tract disorders (excluding obstruction and infection)
			29	Epidermal and dermal conditions
			21	Appetite and general nutritional disorders
			18	Gastrointestinal inflammatory conditions
			15	Hepatic and hepatobiliary disorders
			14	Enzyme investigations NEC
			11	Leukemias
			11	Respiratory disorders NEC
			11	Infections-pathogen unspecified
			10	Procedural related injuries and complications NEC
			10	Renal disorders (excluding nephropathies)
			10	Ocular infections, irritations and inflammations
			9	Adrenal gland disorders/Hepatic and hepatobiliary disorders/Diabetic complications
	Lung, pleura, thymus, and heart	1	386	Gastrointestinal inflammatory conditions
	Digestive system	1	50	Gastrointestinal inflammatory conditions
	Skin	1	154	Gastrointestinal inflammatory conditions
Ipilimumab	Overall	13	2761	Gastrointestinal inflammatory conditions
			866	Psychiatric disorders NEC
			737	Hypothalamus and pituitary gland disorders
			388	Gastrointestinal motility and defecation conditions
			380	Peripheral neuropathies
			220	Hepatic and hepatobiliary disorders
			267	Epidermal and dermal conditions
			259	Infections-pathogen unspecified
			254	Adrenal gland disorders
			166	Gastrointestinal signs and symptoms
			157	Lower respiratory tract disorders (excluding obstruction and infection)
			139	General system disorders NEC
			117	Muscle disorders
			113	Electrolyte and fluid balance conditions
			107	Body temperature conditions
			78	Coagulopathies and bleeding diatheses (excluding thrombocytopenic)
			74	Appetite and general nutritional disorders
			63	Neuromuscular disorders
			59	Peritoneal and retroperitoneal conditions
			56	Gastrointestinal inflammatory conditions
			55	Pericardial disorders
			53	Spleen, lymphatic and reticuloendothelial system disorders
			53	Physical examination and organ system status topics
			47	Encephalopathies
			46	Anemias nonhemolytic and marrow depression
			38	Cranial nerve disorders (excluding neoplasms)
			36	Administration site reactions
			35	Gastrointestinal ulceration and perforation
			34	Thyroid gland disorders
			34	Embolism and thrombosis
			33	Gallbladder disorders
			30	Bile duct disorders
			29	Demyelinating disorders
			27	Nephropathies
			25	Immune disorders NEC
			21	Vascular infections and inflammations
			19	Platelet disorders
			10	Myocardial disorders
	Melanoma	2	1087	Gastrointestinal inflammatory conditions
			388	Gastrointestinal motility and defecation conditions
			255	Epidermal and dermal conditions
			166	Gastrointestinal signs and symptoms
			139	General system disorders NEC
			125	Hypothalamus and pituitary gland disorders
			107	Body temperature conditions
			113	Electrolyte and fluid balance conditions
			84	Hepatic and hepatobiliary disorders
			74	Appetite and general nutritional disorders
			71	Lower respiratory tract disorders (excluding obstruction and infection)
			53	Physical examination and organ system status topics
			46	Anemias nonhemolytic and marrow depression
			42	Adrenal gland disorders
			38	Infections-pathogen unspecified
			35	Gastrointestinal ulceration and perforation
			34	Thyroid gland disorders
			10	Myocardial disorders
	Lung, pleura, thymus, and heart	1	386	Gastrointestinal inflammatory conditions
	Digestive system	1	50	Gastrointestinal inflammatory conditions
	Skin	1	154	Gastrointestinal inflammatory conditions
Atezolizumab	Overall	12	721	Lower respiratory tract disorders (excluding obstruction and infection)
			164	Infections-pathogen unspecified
			158	Gastrointestinal inflammatory conditions
			156	Pleural disorders
			126	Immune disorders NEC
			117	Peripheral neuropathies
			96	Anemias nonhemolytic and marrow depression
			93	Central nervous system disorders
			57	Neoplasm related morbidities
			52	Bile duct disorders
			39	Encephalopathies
			37	Muscle disorders
			27	Gastrointestinal ulceration and perforation
			26	Gastrointestinal hemorrhages NEC
			23	Platelet disorders
			23	Hypothalamus and pituitary gland disorders
			15	Epidermal and dermal conditions
			15	Neuromuscular disorders
			11	Vascular infections and inflammations
			10	Demyelinating disorders
			7	Adrenal gland disorders
			3	Glucose metabolism disorders (including diabetes mellitus)
	NSCLC	1	20	Lower respiratory tract disorders (excluding obstruction and infection)
	Lung, pleura, thymus, and heart	1	67	Gastrointestinal inflammatory conditions
	Digestive system	1	11	Gastrointestinal inflammatory conditions
	Skin	1	4	Gastrointestinal inflammatory conditions
Durvalumab	Overall	9	700	Lower respiratory tract disorders (excluding obstruction and infection)
			125	Pleural disorders
			102	Infections-pathogen unspecified
			47	Gastrointestinal inflammatory conditions
			40	Anemias nonhemolytic and marrow depression
			38	Peripheral neuropathies
			17	Joint disorders
			13	Immune disorders NEC
			12	Platelet disorders
			10	Nervous system disorders
			9	Muscle disorders
			8	Coagulopathies and bleeding diatheses (excluding thrombocytopenic)
			7	Vision disorders
			6	Respiratory disorders NEC
			6	Mycobacterial infectious disorders
			6	Hypothalamus and pituitary gland disorders
			5	Respiratory and mediastinal neoplasms malignant and unspecified
			4	Neuromuscular disorders
			3	Adrenal gland disorders
			1	Vascular infections and inflammations
			1	Glucose metabolism disorders (including diabetes mellitus)
	Lung, pleura, thymus, and heart	1	24	Gastrointestinal inflammatory conditions
	Digestive system	1	2	Gastrointestinal inflammatory conditions
Cemiplimab	Overall	7	21	Immune disorders NEC
			13	Infections-pathogen unspecified
			8	Gastrointestinal inflammatory conditions
			7	Neurological disorders NEC
			5	Aural disorders NEC
			4	Bone and joint injuries
			4	Respiratory disorders NEC
			4	Renal disorders (excluding nephropathies)
			4	Joint disorders
			4	Spleen, lymphatic and reticuloendothelial system disorders
			4	Nephropathies
			3	Platelet disorders
			3	Epidermal and dermal conditions
			3	Bacterial infectious disorders
			3	Deliria (including confusion)
			1	Vision disorders
	Lung, pleura, thymus and heart	1	1	Gastrointestinal inflammatory conditions
	Skin	1	3	Gastrointestinal inflammatory conditions
Pembrolizumab	Overall	15	2049	Lower respiratory tract disorders (excluding obstruction and infection)
			689	Pleural disorders
			436	Infections-pathogen unspecified
			382	Peripheral neuropathies
			283	Anemias nonhemolytic and marrow depression
			159	Muscle disorders
			149	Hypothalamus and pituitary gland disorders
			116	Immune disorders NEC
			115	Neuromuscular disorders
			112	Platelet disorders
			104	Epidermal and dermal conditions
			100	Respiratory disorders NEC
			93	Central nervous system vascular disorders
			93	General system disorders NEC
			81	Glucose metabolism disorders (including diabetes mellitus)
			79	Peritoneal and retroperitoneal conditions
			66	Spleen, lymphatic and reticuloendothelial system disorders
			58	Gallbladder disorders
			53	Adrenal gland disorders
			52	Renal disorders (excluding nephropathies)
			52	Neurological disorders NEC
			31	Vascular infections and inflammations
			29	Hepatic and hepatobiliary disorders
			25	Endocrine and glandular disorders NEC
			22	Demyelinating disorders
			19	Gastrointestinal inflammatory conditions
	Melanoma	1	6	Muscle disorders
	Lung carcinoma	1	6	Muscle disorders
	Lung, pleura, thymus, and heart	1	267	Gastrointestinal inflammatory conditions
	Digestive system	1	14	Gastrointestinal inflammatory conditions
	Skin	1	108	Gastrointestinal inflammatory conditions
	NSCLC	1	47	Lower respiratory tract disorders (excluding obstruction and infection)
Nivolumab + Ipilimumab	Overall	6	1036	Gastrointestinal inflammatory conditions
			138	Immune disorders NEC
			75	Adrenal gland disorders
			67	Gastrointestinal motility and defecation conditions
			64	Epidermal and dermal conditions
			53	Platelet disorders
			48	Body temperature conditions
			40	Thyroid gland disorders
			29	Hepatic and hepatobiliary disorders
			29	Infections-pathogen unspecified
			25	Hepatobiliary investigations
			24	Hypothalamus and pituitary gland disorders
			21	Lower respiratory tract disorders (excluding obstruction and infection)
			17	General system disorders NEC
			16	Gastrointestinal signs and symptoms
			14	Electrolyte and fluid balance conditions
			11	Myocardial disorders
			11	Glucose metabolism disorders (including diabetes mellitus)
	Digestive system cancer	1	27	Gastrointestinal inflammatory conditions
	Skin cancer	1	644	Gastrointestinal inflammatory conditions
	Melanoma	1	67	Gastrointestinal motility and defecation conditions
			65	Gastrointestinal inflammatory conditions
			57	Epidermal and dermal conditions
			48	Body temperature conditions
			40	Thyroid gland disorders
			29	Hepatic and hepatobiliary disorders
			29	Infections-pathogen unspecified
			25	Hepatobiliary investigations
			24	Hypothalamus and pituitary gland disorders
			21	Lower respiratory tract disorders (excluding obstruction and infection)
			17	General system disorders NEC
			16	Gastrointestinal signs and symptoms
			14	Electrolyte and fluid balance conditions
			11	Glucose metabolism disorders (including diabetes mellitus)
			11	Myocardial disorders
Pembrolizumab + Ipilimumab	Overall	4	52	Gastrointestinal inflammatory conditions
			5	Adrenal gland disorders
			2	Platelet disorders
			2	Immune disorders NEC
Cemiplimab + ipilimumab	Overall	1	2	Immune disorders NEC
Pembrolizumab + nivolumab	Overall	0	-	-
Ipilimumab + pembrolizumab + nivolumab	Overall	0	-	-

irAE immune-related adverse event; HLGT high-level group terms; NEC not elsewhere classified; NSCLC non-small cell lung cancer.

**Table 5:** Characteristics of included studies conducted on AHDs.

Characteristics	N (%)
*Studies included*	27 (100)
*Continent*	
Europe	12 (44)
America	11 (41)
Asia	4 (15)
*Study objectives*	
irAE assessment	24 (89)
Other purposes	3 (11)
*Studies reporting the study design*	
Retrospective cohort (overall)	25 (93)
Single-center	2
Multi-center	2
Prospective cohort (overall)	2 (7)
*Data sources used in the studies*	
Electronic medical records	21 (78)
Pharmacy databases and electronic medical records	3 (11)
Disease/drug registries	3 (11)
*Type of the analysis used in the studies*	
Descriptive	10 (37)
Descriptive and analytical	14 (52)
Analytical	3 (11)
*Type of tumors investigated in the studies*	
Melanoma	18 (67)
NSCLC	11 (41)
GI (duodenal, colon, gastric)	4 (15)
Renal	3 (11)
Lung cancer (subtype unspecified)	3 (11)
Head and neck cancer	3 (11)
Genitourinary cancer	3 (11)
Lymphoma	2 (7)
Triple-negative breast cancer	2 (7)
Merkel cell carcinoma	1 (4)
Other cancers not specified	2 (7)
*Drugs investigated in the studies*	
Pembrolizumab (overall)	18 (67)
Pembrolizumab + CT	4
Nivolumab (overall)	14 (52)
Nivolumab + CT	-
Ipilimumab (overall)	6 (22)
Ipilimumab + CT	1
Atezolizumab (overall)	4 (15)
Atezolizumab + CT	-
Durvalumab (overall)	3 (11)
Durvalumab + CT	1
Combination ICI therapies specified	
Nivolumab + ipilimumab	6 (22)
Nivolumab + ipilimumab + T	1 (4)
Other therapies not specified *	4 (15)
*Patients analyzed in the studies (overall)*	7.762
*Gender*	
Overall reported	6.249 (100)
Male reported	3.028 (78)
Female reported	3.221 (52)
*Age*	
Studies reporting the mean value	7 (26)
Mean value (min-max)	63 (50–70)
Studies reporting the median value	18 (67)
*Studies reporting the time to onset of irAEs*	9 (33)
Mean value (min-max), days	130 (2–420)
Studies reporting correspondence overall time to onset-drug-tumor-irAE **	3 (33)
Studies reporting correspondence time to onset-drug-tumor-specific irAE (ipilimumab-melanoma-hypophysitis) ***	1 (11)
Mean value (min-max), days	70 (49–91)
Studies reporting correspondence time to onset-irAE ****	3 (33)
Studies reporting correspondence time to onset-tumor	0
Studies reporting correspondence time to onset-drug *****	2 (22)
*Studies reporting the duration of irAE*	3 (11)
Mean value overall (min-max), days	34 (4–183)
Studies reporting correspondence duration of irAE-drug-tumor-irAE (ipilimumab-melanoma-hypophysitis) ***	1 (33)
Median value to headache resolution (IR) days	7 (4–13)
Studies reporting correspondence duration of irAE-irAE °	2 (67)
*Studies reporting the management of irAEs*	22 (81)
Therapy discontinuation	11 (50)
Corticosteroid therapy	9 (41)
Permanent therapy interruption	3 (14)
Biologic agents	2 (9)
Immunosuppressive drugs	2 (9)
Surgery	1 (5)
Analgesic drugs	1 (5)
Ointment	1 (5)
Glucocorticoids	1 (5)
Levothyroxine	1 (5)
Emollient	1 (5)
DMARD	1 (5)
Calcipotriol	1 (5)
Phototherapy	1 (5)
Hormonal therapy	1 (5)
Immunoglobulins	1 (5)
Transfusion	1 (5)
Granulocyte-colony stimulating factors	1 (5)
Erythropoiesis-stimulating drug	1 (5)
Thrombopoietin agonist	1 (5)
*Studies reporting comorbidities*	10 (37)
Patients with comorbidities, mean value	108 (1)
Studies reporting the specific comorbidity	8 (30)
Autoimmune disease	3 (38)
Pre-existing liver disease	2 (25)
Hypopituitarism	1 (13)
Pre-existing type II diabetes	1 (13)
Pre-existing pulmonary comorbidities	1 (13)
Pre-existing cardiac disease	1 (13)
Infections	1 (13)
Systemic sclerosis	1 (13)
*Studies reporting concomitant therapies*	4 (15)
Patients with concomitant therapies, mean value	62 (1)
Studies reporting the specific comorbidity	4 (15)
Second-line immunosuppressive therapy in addition to corticosteroids	1 (25)
Systemic immunosuppression	2 (50)
PPI	1 (25)
Antibiotic	1 (25)
Corticosteroids	1 (25)
*Studies reporting the outcome of irAE (overall)*	14 (52)
Death	9 (64)
HO	5 (36)
*Studies reporting the survival analysis °°*	
OS	15 (56)
PFS	13 (48)
OS and PFS	12 (44)
Studies reporting correspondence between OS/PFS-tumor-irAE	1 (4)
Median OS value reported for melanoma and hepatitis, months	17 (no range)
Median PFS value reported for melanoma and hepatitis, months	6 (no range)
Studies reporting correspondence between OS/PFS-drug-tumor	8 (30)
Studies reporting correspondence between OS-pembrolizumab-NSCLC	4 (15)
Mean OS value reported for NSCLC treated with pembrolizumab, months (range)	16 (9–22)
Studies reporting correspondence between PFS-pembrolizumab-NSCLC	3 (11)
Mean PFS value reported for NSCL treated with pembrolizumab, months (range)	9 (4–14)
Studies reporting correspondence between OS/PFS-pembrolizumab-melanoma	2 (7)
Mean OS value reported for melanoma treated with pembrolizumab, months (range)	83 (15–212)
Mean PFS value reported for melanoma treated with pembrolizumab, months (range)	36 (5–118)
Studies reporting correspondence between OS/PFS-pembrolizumab-TNBC	1 (4)
OS value reported for TNBC treated with pembrolizumab, years	2.4
PFS value reported for TNBC treated with pembrolizumab, years	2.4
Studies reporting correspondence between OS/PFS-ipilimumab-melanoma	1 (4)
Median OS value reported for melanoma treated with ipilimumab, months (range)	23 (16–36)
Median PFS value reported for melanoma treated with ipilimumab, months (range)	20 (3–78)
Studies reporting correspondence between OS/PFS-nivolumab-melanoma	1 (4)
Median OS value reported for melanoma treated with nivolumab, months (range)	20 (4–35)
Median PFS value reported for melanoma treated with nivolumab, months (range)	10 (6–15)
Studies reporting correspondence between OS-melanoma	3 (11)
Mean OS value reported for melanoma in patients with irAE, months (range)	22 (4–NR)
Studies reporting correspondence between PFS-melanoma	3 (11)
Mean PFS value reported for melanoma in patients with irAE, months (range)	3 (2–4)
Studies reporting correspondence between OS/PFS-NSCLC	2 (7)
Mean OS value reported for NSCLC in patients with irAE, months (range)	42 (6–44)
Mean PFS value reported for NSCLC in patients with irAE, months (range)	9 (4–17)
Studies reporting correspondence between OS/PFS-GC	1 (4)
Median OS value reported for GC in patients with irAE, months (range)	5 (2–7)
Median PFS value reported for GC in patients with irAE, months (range)	2 (2–3)
*Studies reporting dechallenge and rechallenge*	0

* Other drugs not specified: “other PD-1 inhibitors”, “anti-PD-(L)1”, “anti-PD-(L)1 + investigational drug”, “anti-PD-1+ anti-CTLA4”. ** Ansel et al. (2023) [31] reported the overall time to onset for irAEs of NSCLC patients treated with pembrolizumab. Byrne et al. 2021 [47] reported the overall time to onset for the reported irAE. Egami et al. (2021) [26] reported the overall time to onset for irAEs of NSCLC patients treated with nivolumab. *** Faje et al. (2018) [25] reported the correspondence time to onset-ipilimumab-melanoma-hypophysitis and the correspondence duration of irAE-ipilimumab-hypophysitis. **** Conde-Estévez et al. (2020) [13] reported the time to onset in NSCLC patients for each of the following irAE: dermatological, endocrinological, gastrointestinal, hepatological, arthralgia/myalgia, and pulmonary. Delanoy et al. (2018) [27] reported the time to onset for each of the following irAE: hematological-irAE, neutropenia, autoimmune hemolytic anemia, immune thrombocytopenia, pancytopenia or aplastic anemia, bicytopenia, and pure red cell aplasia. Hata et al. (2022) [28] reported the time to onset in melanoma patients for each of the following irAE: dermatitis, hepatitis, fever, pneumonitis, fatigue, thyroiditis, diarrhea/colitis, hypoadenocorticism, oral mucosal toxicities, nervous system disorder, musculoskeletal disorders, gastrointestinal disorders (except diarrhea/hepatitis), renal toxicities, electrolyte abnormality, eye disorder, type I diabetes mellitus, and hypopituitarism. ***** Biewenga et al. (2021) [20] reported the time to onset for PD-1 inhibitors, ipilimumab, and ipilimumab + nivolumab used in melanoma patients. Jessel et al. (2022) [48] reported the time to onset for ipilimumab + nivolumab, anti-PD-(L)1, and ipilimumab used for treating several tumors. ° Byrne et al. (2021) [47] reported the duration of irAE for all the irAEs reported. Delanoy et al. (2018) [27] reported the duration of irAE for each of the following irAE: hematological-irAE, neutropenia, autoimmune hemolytic anemia, immune thrombocytopenia, pancytopenia or aplastic anemia, bicytopenia, and pure red cell aplasia. °° No study has a specific correspondence between OS/PFS tumor, drug and irAE. AHDs Administrative Healthcare Databases; NSCLC non-small cell lung cancer; GI gastrointestinal; OS overall survival; PFS progression free survival; TNBC triple negative breast cancer; ICI immune checkpoint inhibitors; irAE immune-related adverse events; DMARD disease-modifying antirheumatic drugs; IR interquartile range; CT chemotherapy; T therapy; PPI proton pump inhibitor.

**Table 6:** irAEs of any grade reported for each drug in the included studies conducted on AHDs (HLGT classification).

	Tumor	Number of Studies Reporting the Drug for the Tumor and irAE	Number of irAE Any Grade	HLGT
Pembrolizumab	Overall	7	96	Epidermal and dermal conditions
			77	Thyroid gland disorders
			58	Lower respiratory tract disorders (excluding obstructions and infection)
			52	Hepatobiliary investigations
			43	Gastrointestinal motility and defecation conditions
			41	Gastrointestinal inflammatory conditions
			30	Hepatic and hepatobiliary disorders
			27	Anemias nonhemolytic and marrow depression
			26	General systemic disorders NEC
			26	Hypothalamus and pituitary gland disorders/adrenal gland disorders
			14	Joint symptoms
			14	Joint disorders
			8	Platelet disorders
			8	White blood cell disorders
			7	Gastrointestinal signs and symptoms
			7	Pigmentation disorders
			5	Body temperature conditions
			4	Oral soft tissue conditions
			4	Muscle disorders
			3	Endocrine and glandular disorders NEC
			2	Embolism and thrombosis
			2	Central nervous system infections and inflammations
			2	Bile duct disorders
			2	Glucose metabolism disorders (including diabetes mellitus)
			1	Peripheral neuropathies
			1	Vascular infections and inflammations
			1	Procedural related injuries and complications NEC
			1	White blood cell disorders
			16	Other
	TNBC	1	11	Thyroid gland disorders
			9	Adrenal gland disorders
			6	Joint disorders
			4	Gastrointestinal inflammatory conditions
			3	Epidermal and dermal conditions
			3	Lower respiratory tract disorders (excluding obstructions and infection)
			3	Hepatic and hepatobiliary disorders
			1	Muscle disorders
			1	Central nervous system infections and inflammations
			1	White blood cell disorders
	Melanoma	1	53	Epidermal and dermal conditions
			35	Hepatobiliary investigations
			33	Thyroid gland disorders
			14	Joint symptoms
			13	Gastrointestinal motility and defecation conditions
			8	General systemic disorders NEC
			7	Lower respiratory tract disorders (excluding obstructions and infection)
			7	Pigmentation disorders
			12	Other
	NSCLC	4	68	Epidermal and dermal conditions
			48	Lower respiratory tract disorders (excluding obstructions and infection)
			40	Gastrointestinal motility and defecation conditions
			37	Gastrointestinal inflammatory conditions
			33	Thyroid gland disorders
			27	Anemias nonhemolytic and marrow depression
			27	Hepatic and hepatobiliary disorders
			17	Hepatobiliary investigations
			17	Hypothalamus and pituitary gland disorders/adrenal gland disorders
			15	General systemic disorders NEC
			10	Renal and urinary tract investigations and urinalyses
			9	Nephropathies
			8	Platelet disorders
			8	White blood cell disorders
			8	Joint disorders
			7	Gastrointestinal signs and symptoms
			5	Body temperature conditions
			4	Oral soft tissue conditions
			3	General systema disorders NEC
			3	Endocrine and glandular disorders NEC
			3	Muscle disorders
			2	Embolism and thrombosis
			2	Bile duct disorders
			2	Glucose metabolism disorders (including diabetes mellitus)
			1	Peripheral neuropathies
			1	Vascular infections and inflammations
			1	Central nervous system infections and inflammations
			1	Procedural related injuries and complications NEC
			4	Other
Nivolumab	Overall	3	458	Epidermal and dermal conditions
			285	General system disorders NEC
			219	Gastrointestinal conditions NEC
			200	Musculoskeletal and connective tissue disorders *
			197	Endocrine and glandular disorders NEC
			138	Neurological disorders NEC
			113	Procedural related injuries and complications NEC
			99	Respiratory disorders NEC
			89	Eye disorders NEC
			80	Hepatic and hepatobiliary disorders
			35	Nephropathies
			34	Thyroid gland disorders
			31	Gastrointestinal motility and defecation conditions
			15	Hepatobiliary investigations
			11	Lower respiratory tract disorders (excluding obstructions and infection)
			6	Gastrointestinal inflammatory conditions
			6	Gastrointestinal signs and symptoms
			5	Adrenal gland disorders
			4	Hematology investigations (including blood groups)
			3	Renal disorders (excluding nephropathies)
			2	Exocrine pancreas conditions
			1	Headaches
			1	Allergic conditions
			1	Central nervous system infections and inflammations
			1	Neuromuscular disorders
			1	Embolism and thrombosis
			20	Other
	NSCLC	1	44	Epidermal and dermal conditions
			20	Gastrointestinal motility and defecation conditions
			15	Thyroid gland disorders
			3	Hepatic and hepatobiliary disorders
			2	Lower respiratory tract disorders (excluding obstructions and infection)
			1	Central nervous system infections and inflammations
			1	Neuromuscular disorders
			1	Embolism and thrombosis
	Melanoma	1	387	Epidermal and dermal conditions
			267	General system disorders NEC
			219	Gastrointestinal conditions NEC
			200	Musculoskeletal and connective tissue disorders *
			197	Endocrine and glandular disorders NEC
			138	Neurological disorders NEC
			113	Procedural related injuries and complications NEC
			99	Respiratory disorders NEC
			89	Eye disorders NEC
			77	Hepatic and hepatobiliary disorders
			35	Nephropathies
			4	Hematology investigations (including blood groups)
Ipilimumab	Overall = melanoma	1	106	Gastrointestinal inflammatory conditions
			19% **	Hypothalamus and pituitary gland disorders
			8% **	Epidermal and dermal conditions
			8% **	Adrenal gland disorders
			8% **	Thyroid gland disorders
			6% **	Hepatobiliary investigations
			3% **	Neurological disorders NEC
			3% **	Anemias nonhemolytic and marrow depression
			17% **	Other
Durvalumab	Overall = NSCLC	1	2	Autoimmune disorders
			1	Gastrointestinal motility and defecation conditions
			1	Pigmentation disorders
			1	Gastrointestinal ulceration and perforation
			1	Hepatic and hepatobiliary disorders
Atezolizumab	Overall	0	-	-
Nivolumab + ipilimumab	Overall	0	-	-

* IrAE reported as SOC. ** Percentage reported in the study. irAE immune-related adverse event; HLGT high-level group terms; NEC not elsewhere classified; NSCLC non-small cell lung cancer.

## Data Availability

The original contributions presented in this study are included in the article/Supplementary Materials. Further inquiries can be directed to the corresponding author.

## Supplementary Materials

The following supporting information can be downloaded at: https://www.mdpi.com/article/10.3390/ph19020276/s1, Table S1: Biologic target, therapeutic indications, date of approval, and safety profile of ICIs of interest approved by EMA and FDA: information common to both agencies (HLGT classification); Table S2: Date of approval, biologic target, therapeutic indications, and safety profile of ICIs of interest approved by EMA and FDA: information specific to each agency (HLGT); Table S3: Biologic target, therapeutic indications, date of approval, and safety profile of ICIs of interest approved by EMA and FDA: information common to both agencies (HLT classification); Table S4: Therapeutic indications, date of approval, and safety profile of ICIs of interest approved by EMA and FDA: information specific to each agency (HLT classification); File Text S1; Table S5: irAEs reported for each drug in the included studies conducted on SRSs (HLT classification); Table S6: irAEs reported only as SOC in the included studies conducted on SRSs; File Text S2; Table S7: irAEs of grade 3 or more for each drug in the included studies conducted on AHDs (HLGT classification); Table S8: irAEs of any grade reported for each drug in the included studies conducted on AHDs (HLT classification); Table S9: irAEs of grade 3 or more reported for each drug in the included studies conducted on AHDs (HLT classification); Table S10: Preferred Reporting Items for Systematic Reviews and Meta-Analyses Extension for Scoping Reviews (PRISMA-ScR) Checklist [81]; File Text S3.

## Abbreviations

The following abbreviations are used in this manuscript:

ADEs	Adverse drug events
AHDs	Administrative Healthcare Databases
CTLA 4	Cytotoxic T-lymphocyte antigen 4
EMA	European Medicines Agency
FDA	Food and Drug Administration
GI	Gastrointestinal
HLGTs	High-level group terms
HLTs	High-level terms
ICIs	Immune checkpoint inhibitors
IrAEs	Immune-related adverse events
MA	Medicines Agency
MedDRA	Medical Dictionary for Regulatory Activities
NEC	Not elsewhere classified
NSCLC	Non-small cell lung cancer
OS	Overall survival
PD-1	Programmed cell death protein 1
PD-L1	Programmed cell death protein ligand 1
PFS	Progression free survival
PRISMA	Preferred Reporting Items for Systematic reviews and Meta-Analyses
SCLC	Small cell lung cancer
SOC	System organ class
SPC	Specific product characteristic
SRSs	Spontaneous reporting systems
TNBC	Triple negative breast cancer

## References

[1] Yin Q., Wu L., Han L., Zheng X., Tong R., Li L., Bai L., Bian Y. (2023). Immune-related adverse events of immune checkpoint inhibitors: A review. Front. Immunol..

[2] Chennamadhavuni A., Abushahin L., Jin N., Presley C.J., Manne A. (2022). Risk Factors and Biomarkers for Immune-Related Adverse Events: A Practical Guide to Identifying High-Risk Patients and Rechallenging Immune Checkpoint Inhibitors. Front. Immunol..

[3] Sandigursky S., Mor A. (2018). Immune-Related Adverse Events in Cancer Patients Treated with Immune Checkpoint Inhibitors. Curr. Rheumatol. Rep..

[4] Dang Q.M., Watanabe R., Shiomi M., Fukumoto K., Nobashi T.W., Okano T., Yamada S., Hashimoto M. (2023). Rheumatic Immune-Related Adverse Events due to Immune Checkpoint Inhibitors—A 2023 Update. Int. J. Mol. Sci..

[5] Guidon A.C., Burton L.B., Chwalisz B.K., Hillis J., Schaller T.H., Amato A.A., Warner A.B., Brastianos P.K., Cho T.A., Clardy S.L. (2021). Consensus disease definitions for neurologic immune-related adverse events of immune checkpoint inhibitors. J. Immunother. Cancer.

[6] Khoja L., Day D., Wei-Wu Chen T., Siu L.L., Hansen A.R. (2017). Tumour- and class-specific patterns of immune-related adverse events of immune checkpoint inhibitors: A systematic review. Ann. Oncol..

[7] Barron C.C., Stefanova I., Cha Y., Elsolh K., Zereshkian A., Gaafour N., McWhirter E. (2023). Chronic immune-related adverse events in patients with cancer receiving immune checkpoint inhibitors: A systematic review. J. Immunother. Cancer.

[8] Das S., Johnson D.B. (2019). Immune-related adverse events and anti-tumor efficacy of immune checkpoint inhibitors. J. Immunother. Cancer.

[9] European Medicine Agency EMA Webpage. https://www.ema.europa.eu/en/medicines.

[10] Food and Drug Administration FDA Webpage. https://www.accessdata.fda.gov/scripts/cder/daf/index.cfm?event=BasicSearch.process.

[11] Pokorny R., McPherson J.P., Haaland B., Grossmann K.F., Luckett C., Voorhies B.N., Sageser D.S., Wallentine J., Tolman Z., Hu-Lieskovan S. (2021). Real-world experience with elective discontinuation of PD-1 inhibitors at 1 year in patients with metastatic melanoma. J. Immunother. Cancer.

[12] Suo A., Chan Y., Beaulieu C., Kong S., Cheung W.Y., Monzon J.G., Smylie M., Walker J., Morris D., Cheng T. (2020). Anti-PD1-Induced Immune-Related Adverse Events and Survival Outcomes in Advanced Melanoma. Oncologist.

[13] Conde-Estévez D., Monge-Escartín I., Ríos-Hoyo A., Monzonis X., Echeverría-Esnal D., Moliner L., Duran-Jordà X., Taus Á., Arriola E. (2020). Prognostic factors and effect on survival of immune-related adverse events in patients with non-small-cell lung cancer treated with immune checkpoint blockage. J. Chemother..

[14] Panhaleux M., Espitia O., Terrier B., Manson G., Maria A., Humbert S., Godbert B., Perrin J., Achille A., Arrondeau J. (2022). Anti–programmed death ligand 1 immunotherapies in cancer patients with pre-existing systemic sclerosis: A postmarketed phase IV safety assessment study. Eur. J. Cancer.

[15] Anpalakhan S., Huddar P., Behrouzi R., Signori A., Cave J., Comins C., Cortellini A., Addeo A., Escriu C., McKenzie H. (2023). Immunotherapy-related adverse events in real-world patients with advanced non-small cell lung cancer on chemoimmunotherapy: A Spinnaker study sub-analysis. Front. Oncol..

[16] Dudnik E., Moskovitz M., Rottenberg Y., Lobachov A., Mandelboim R., Shochat T., Urban D., Wollner M., Nechushtan H., Rotem O. (2021). Pembrolizumab as a monotherapy or in combination with platinum-based chemotherapy in advanced non-small cell lung cancer with PD-L1 tumor proportion score (TPS) ≥50%: Real-world data. OncoImmunology.

[17] Sattar J., Kartolo A., Hopman W.M., Lakoff J.M., Baetz T. (2019). The efficacy and toxicity of immune checkpoint inhibitors in a real-world older patient population. J. Geriatr. Oncol..

[18] Hassanzadeh C., Sita T., Savoor R., Samson P.P., Bradley J., Gentile M., Roach M., Mohindra N., Waqar S., Kruser T.J. (2020). Implications of pneumonitis after chemoradiation and durvalumab for locally advanced non-small cell lung cancer. J. Thorac. Dis..

[19] Hribernik N., Boc M., Ocvirk J., Knez-Arbeiter J., Mesti T., Ignjatovic M., Rebersek M. (2020). Retrospective analysis of treatment-naive Slovenian patients with metastatic melanoma treated with pembrolizumab-real-world experience. Radiol. Oncol..

[20] Biewenga M., van der Kooij M.K., Wouters M.W.J.M., Aarts M.J.B., Berkmortel F.W.P.J.v.D., de Groot J.W.B., Boers-Sonderen M.J., Hospers G.A.P., Piersma D., van Rijn R.S. (2021). Checkpoint inhibitor induced hepatitis and the relation with liver metastasis and outcome in advanced melanoma patients. Hepatol. Int..

[21] Jurlander R.S., Guldbrandt L.M., Holmstroem R.B., Madsen K., Donia M., Haslund C.A., Schmidt H., Bastholt L., Ruhlmann C.H., Svane I.M. (2024). Immune-related adverse events in a nationwide cohort of real-world melanoma patients treated with adjuvant anti-PD1—Seasonal variation and association with outcome. Eur. J. Cancer.

[22] Tambo Y., Sone T., Shibata K., Nishi K., Shirasaki H., Yoneda T., Araya T., Kase K., Nishikawa S., Kimura H. (2020). Real-World Efficacy of First-Line Pembrolizumab in Patients with Advanced or Recurrent Non–Small-Cell Lung Cancer and High PD-L1 Tumor Expression. Clin. Lung Cancer.

[23] Krishnan J., Patel A., Roy A.M., Alharbi M., Kapoor A., Yao S., Khoury T., Hong C.-C., Held N., Chakraborty A. (2024). Detrimental Impact of Chemotherapy Dose Reduction or Discontinuation in Early Stage Triple-Negative Breast Cancer Treated With Pembrolizumab and Neoadjuvant Chemotherapy: A Multicenter Experience. Clin. Breast Cancer.

[24] Karam J.D., Noel N., Voisin A.L., Lanoy E., Michot J.M., Lambotte O. (2020). Infectious complications in patients treated with immune checkpoint inhibitors. Eur. J. Cancer.

[25] Faje A.T., Lawrence D., Flaherty K., Freedman C., Fadden R., Rubin K., Cohen J., Sullivan R.J. (2018). High-dose glucocorticoids for the treatment of ipilimumab-induced hypophysitis is associated with reduced survival in patients with melanoma. Cancer.

[26] Egami S., Kawazoe H., Hashimoto H., Uozumi R., Arami T., Sakiyama N., Ohe Y., Nakada H., Aomori T., Ikemura S. (2021). Absolute Lymphocyte Count Predicts Immune-Related Adverse Events in Patients with Non-Small-Cell Lung Cancer Treated with Nivolumab Monotherapy: A Multicenter Retrospective Study. Front. Oncol..

[27] Delanoy N., Michot J.-M., Comont T., Kramkimel N., Lazarovici J., Dupont R., Champiat S., Chahine C., Robert C., Herbaux C. (2019). Haematological immune-related adverse events induced by anti-PD-1 or anti-PD-L1 immunotherapy: A descriptive observational study. Lancet Haematol..

[28] Hata H., Matsumura C., Chisaki Y., Nishioka K., Tokuda M., Miyagi K., Suizu T., Yano Y. (2022). A Retrospective Cohort Study of Multiple Immune-Related Adverse Events and Clinical Outcomes Among Patients with Cancer Receiving Immune Checkpoint Inhibitors. Cancer Control.

[29] Franken M.G., Leeneman B., Jochems A., Schouwenburg M.G., Aarts M.J., van Akkooi A.C., Berkmortel F.W.v.D., Eertwegh A.J.v.D., de Groot J.W.B., van der Hoeven K.J. (2018). Real-world healthcare costs of ipilimumab in patients with advanced cutaneous melanoma in the Netherlands. Anticancer Drugs.

[30] Elijah J., Puzanov I., Cresanti B., Hamad L., Attwood K., Catalfamo K., Riebandt G. (2024). Evaluation of safety outcomes between nivolumab regimens with differing dosing patterns. J. Oncol. Pharm. Pract..

[31] Ansel S., Rulach R., Trotter N., Steele N. (2023). Pembrolizumab for advanced non-small cell lung cancer (NSCLC): Impact of autoimmune comorbidity and outcomes following treatment completion. J. Oncol. Pharm. Pract..

[32] Huang S., Bai X., Fang T., Guo Y., Zheng K., Lin X. (2021). Gastrointestinal toxicities associated with immune checkpoint inhibitors: A disproportionality analysis leveraging VigiBase, the WHO Adverse Drug Reaction Database. J. Zhejiang Univ. Sci. B.

[33] Shah K.P., Song H., Ye F., Moslehi J.J., Balko J.M., Salem J.-E., Johnson D.B. (2020). Demographic factors associated with toxicity in patients treated with anti-programmed cell death-1 therapy. Cancer Immunol. Res..

[34] Oshima Y., Tanimoto T., Yuji K., Tojo A. (2018). EGFR-TKI-associated interstitial pneumonitis in nivolumab-treated patients with non-small cell lung cancer. JAMA Oncol..

[35] Grouthier V., Lebrun-Vignes B., Moey M., Johnson D.B., Moslehi J.J., Salem J.-E., Bachelot A. (2020). Immune Checkpoint Inhibitor-Associated Primary Adrenal Insufficiency: WHO VigiBase Report Analysis. Oncologist.

[36] Hu Y., Gong J., Zhang L., Li X., Li X., Zhao B., Hai X. (2020). Colitis following the use of immune checkpoint inhibitors: A real-world analysis of spontaneous reports submitted to the FDA adverse event reporting system. Int. Immunopharmacol..

[37] Cutroneo P., Ingrasciotta Y., Isgrò V., Rullo E.V., Berretta M., Fiorica F., Trifirò G., Guarneri C. (2021). Psoriasis and psoriasiform reactions secondary to immune checkpoint inhibitors. Dermatol. Ther..

[38] Nguyen L.S., Ortuno S., Lebrun-Vignes B., Johnson D.B., Moslehi J.J., Hertig A., Salem J.-E. (2021). Transplant rejections associated with immune checkpoint inhibitors: A pharmacovigilance study and systematic literature review. Eur. J. Cancer.

[39] Xi X., Yan X., Chen Y., Li W., Dong J., Ou X., Tan H. (2024). Cytokine release syndrome associated with immune checkpoint inhibitors: A pharmacovigilance study based on spontaneous reports in FAERS. Expert Opin. Drug Saf..

[40] Trenque T., Lepoix E., Trenque A., Morel A., Azzouz B. (2022). Immune-mediated necrotizing myopathy with pembrolizumab: A specific neuromuscular entity. Eur. J. Clin. Pharmacol..

[41] Alghamdi E.A., Aljohani H., Alghamdi W., Alharbi F. (2022). Immune checkpoint inhibitors and potential risk of thromboembolic events: Analysis of the WHO global database of individual case safety reports. Saudi Pharm. J..

[42] Yang F., Shay C., Abousaud M., Tang C., Li Y., Qin Z., Saba N.F., Teng Y. (2023). Patterns of toxicity burden for FDA-approved immune checkpoint inhibitors in the United States. J. Exp. Clin. Cancer Res..

[43] Tyagi S., Kumar A. (2024). Safety of immune checkpoint inhibitors: An updated comprehensive disproportionality analysis and meta-analysis. Crit. Rev. Oncol..

[44] Connors C., Valente S.A., ElSherif A., Escobar P., Chichura A., Kopicky L., Roesch E., Ritner J., McIntire P., Wu Y. (2024). Real-World Outcomes with the KEYNOTE-522 Regimen in Early-Stage Triple-Negative Breast Cancer. Ann. Surg. Oncol..

[45] Gawaz A., Wolff I., Nanz L., Flatz L., Forschner A. (2024). Efficacy of adjuvant immune checkpoint inhibitors pembrolizumab or nivolumab in melanoma patients ≥ 75 years: Results of a real-world cohort including 456 patients. Cancer Immunol. Immunother..

[46] Kuzmanovszki D., Kiss N., Tóth B., Kerner T., Tóth V., Szakonyi J., Lőrincz K., Hársing J., Imrédi E., Pfund A. (2022). Anti-PD-1 Monotherapy in Advanced Melanoma-Real-World Data from a 77-Month-Long Retrospective Observational Study. Biomedicines.

[47] Byrne M.M., Lucas M., Pai L., Breeze J., Parsons S.K. (2021). Immune-related adverse events in cancer patients being treated with immune checkpoint inhibitors. Eur. J. Haematol..

[48] Jessel S., Weiss S.A., Austin M., Mahajan A., Etts K., Zhang L., Aizenbud L., Perdigoto A.L., Hurwitz M., Sznol M. (2022). Immune Checkpoint Inhibitor-Induced Hypophysitis and Patterns of Loss of Pituitary Function. Front. Oncol..

[49] Garcia C.R., Jayswal R., Adams V., Anthony L.B., Villano J.L. (2019). Multiple sclerosis outcomes after cancer immunotherapy. Clin. Transl. Oncol..

[50] Ebinama U., Sheshadri A., Anand K., Swaminathan I. (2023). Pulmonary Immune-Related Adverse Events of PD-1 Versus PD-L1 Checkpoint Inhibitors: A Retrospective Review of Pharmacovigilance. J. Immunother. Precis. Oncol..

[51] Liu G., Zhang S., Mo Z., Huang T., Yu Q., Lu X., He P. (2024). Association of thrombocytopenia with immune checkpoint inhibitors: A large-scale pharmacovigilance analysis based on the data from FDA adverse event reporting system database. Front. Pharmacol..

[52] Mikami T., Liaw B., Asada M., Niimura T., Zamami Y., Green-LaRoche D., Pai L., Levy M., Jeyapalan S. (2021). Neuroimmunological adverse events associated with immune checkpoint inhibitor: A retrospective, pharmacovigilance study using FAERS database. J. Neurooncol..

[53] Grabska S., Grabski H., Makunts T., Abagyan R. (2024). Co-Occurring Infections in Cancer Patients Treated with Checkpoint Inhibitors Significantly Increase the Risk of Immune-Related Adverse Events. Cancers.

[54] Chen J., Wen Y., Chu X., Liu Y., Su C. (2022). Pulmonary adverse events associated with hypertension in non-small cell lung cancer patients receiving PD-1/PD-L1 inhibitors. Front. Pharmacol..

[55] Soldatos T.G., Dimitrakopoulou-Strauss A., Larribere L., Hassel J.C., Sachpekidis C. (2018). Retrospective Side Effect Profiling of the Metastatic Melanoma Combination Therapy Ipilimumab-Nivolumab Using Adverse Event Data. Diagnostics.

[56] Zhou H., Liu J., Zhang Y., Zhang L. (2019). Inflammatory bowel disease associated with the combination treatment of nivolumab and metformin: Data from the FDA adverse event reporting system. Cancer Chemother. Pharmacol..

[57] Takada S., Hirokazu H., Yamagishi K., Hideki S., Masayuki E. (2020). Predictors of the Onset of Type 1 Diabetes Obtained from Real-World Data Analysis in Cancer Patients Treated with Immune Checkpoint Inhibitors. Asian Pac. J. Cancer Prev..

[58] Ali A.K., Watson D.E. (2017). Pharmacovigilance Assessment of Immune-Mediated Reactions Reported for Checkpoint Inhibitor Cancer Immunotherapies. Pharmacotherapy.

[59] CIOMS Pharmacovigilance—CIOMS. CIOMS Website. https://cioms.ch/pharmacovigilance/.

[60] ICH ICH Official Web Site: ICH. ICH Website. https://www.ich.org.

[61] (2005). ICH Topic E 2 E Pharmacovigilance Planning (Pvp) Step 5 Note for Guidance on Planning Pharmacovigilance Activities Pharmacovigilance Planning: Planning of Pharmacovigilance Activities Table of Content. https://www.ema.europa.eu/en/ich-e2e-pharmacovigilance-planning-pvp-scientific-guideline.

[62] Yoest J.M. (2017). ImmunoTargets and Therapy Dovepress Clinical features, predictive correlates, and pathophysiology of immune-related adverse events in immune checkpoint inhibitor treatments in cancer: A short review. ImmunoTargets Ther..

[63] Hazell L., Shakir S.A.W. (2006). Under-reporting of adverse drug reactions: A systematic review. Drug Saf..

[64] Toussi M., Layton D., Wang S.V. (2018). Study Designs for Post-Authorization Safety Studies. Post-Authorization Safety Studies of Medicinal Products: The PASS Book.

[65] Smith S.M., Wachter K., Burris H.A., Schilsky R.L., George D.J., Peterson D.E., Johnson M.L., Markham M.J., Mileham K.F., Beg M.S. (2021). Clinical Cancer Advances 2021: ASCO’s Report on Progress Against Cancer. J. Clin. Oncol..

[66] Guidelines|ESMO. https://www.esmo.org/guidelines.

[67] Zhou X., Yao Z., Yang H., Liang N., Zhang X., Zhang F. (2020). Are immune-related adverse events associated with the efficacy of immune checkpoint inhibitors in patients with cancer? A systematic review and meta-analysis. BMC Med..

[68] Zhong L., Wu Q., Chen F., Liu J., Xie X. (2021). Immune-related adverse events: Promising predictors for efficacy of immune checkpoint inhibitors. Cancer Immunol. Immunother..

[69] Hussaini S., Chehade R., Boldt R.G., Raphael J., Blanchette P., Vareki S.M., Fernandes R. (2021). Association between immune-related side effects and efficacy and benefit of immune checkpoint inhibitors—A systematic review and meta-analysis. Cancer Treat. Rev..

[70] Cortellini A., Buti S., Agostinelli V., Bersanelli M. (2019). A systematic review on the emerging association between the occurrence of immune-related adverse events and clinical outcomes with checkpoint inhibitors in advanced cancer patients. Semin. Oncol..

[71] Yan W., Qin L., Han Y., Jia X., Wu J. (2025). Impact of Immune-Related Adverse Events on Survival in Patients with Gastrointestinal Cancer Treated with Immune Checkpoint Inhibitors: A Meta-Analysis. Clin. Transl. Gastroenterol..

[72] Hwang S.Y., Rezaee-Zavareh M.S., Attia A.M., Kaymen E.A., Tran N., Abou-Alfa G.K., Parikh N.D., Singal A.G., Yang J.D. (2025). Immune-Related Adverse Events Are Associated with Improved Outcomes After Immune Checkpoint Inhibitor Treatment in Hepatocellular Carcinoma: A Systematic Review and Meta-Analysis. Am. J. Gastroenterol..

[73] Amoroso V., Gallo F., Alberti A., Paloschi D., Bravo W.F., Esposito A., Cosentini D., Grisanti S., Pedersini R., Petrelli F. (2023). Immune-related adverse events as potential surrogates of immune checkpoint inhibitors’ efficacy: A systematic review and meta-analysis of randomized studies. ESMO Open.

[74] Ghisoni E., Wicky A., Bouchaab H., Imbimbo M., Delyon J., Moura B.G., Gérard C., Latifyan S., Özdemir B., Caikovski M. (2021). Late-onset and long-lasting immune-related adverse events from immune checkpoint-inhibitors: An overlooked aspect in immunotherapy. Eur. J. Cancer.

[75] Noguchi Y., Ueno A., Otsubo M., Katsuno H., Sugita I., Kanematsu Y., Yoshida A., Esaki H., Tachi T., Teramachi H. (2018). A simple method for exploring adverse drug events in patients with different primary diseases using spontaneous reporting system. BMC Bioinform..

[76] Trifirò G., Gini R., Barone-Adesi F., Beghi E., Cantarutti A., Capuano A., Carnovale C., Clavenna A., Dellagiovanna M., Ferrajolo C. (2019). The Role of European Healthcare Databases for Post-Marketing Drug Effectiveness, Safety and Value Evaluation: Where Does Italy Stand?. Drug Saf..

[77] Xu Z., Kass-Hout T., Anderson-Smits C., Gray G. (2015). Signal detection using change point analysis in postmarket surveillance. Pharmacoepidemiol. Drug Saf..

[78] Trifirò G. Real World Evidence e Farmaci. http://ebookcentral.proquest.com/lib/unicatt-ebooks/detail.action?docID=5982935.

[79] Scoping—PRISMA Statement. https://www.prisma-statement.org/scoping.

[80] Tacchi C., Convertino I., Bocci G. (2025). Immune-Related Adverse Events Associated with Immune Checkpoint Inhibitors: A Scoping Review Protocol. OSF Preprints.

[81] Tricco A.C., Lillie E., Zarin W., O’Brien K.K., Colquhoun H., Levac D., Moher D., Peters M.D.J., Horsley T., Weeks L. (2018). PRISMA Extension for Scoping Reviews (PRISMAScR): Checklist and Explanation. Ann. Intern. Med..

