# Supplementary material for: Immune-Related Adverse Events Associated with Immune Checkpoint Inhibitors: A Scoping Review
Source: Pharmaceuticals (Basel). 2026 Feb 6;19(2):276. doi: 10.3390/ph19020276 (PMC12943639; doi:10.3390/ph19020276)
Supplement: Supplementary file 1 [file pharmaceuticals-19-00276-s001.zip › pharmaceuticals-4067805-supplementary.pdf]

Immune-Related Adverse Events Associated with Immune Checkpoint Inhibitors: A Scoping Review

Costanza Tacchi[1], Irma Convertino[2,3*], Guido Bocci[1].

1. Department of Translational Research and New Technologies in Medicine and Surgery, University of Pisa, Pisa 56126, Italy;
2. Unit of Pharmacology and Pharmacovigilance, Department of Clinical and Experimental Medicine, University of Pisa, Pisa 56126, Italy;
3. Regional Pharmacovigilance Center of Tuscany, Florence 50139, Italy;

*Corresponding author

Irma Convertino, PharmD, PhD
Unit of Pharmacology and Pharmacovigilance
Department of Clinical and Experimental Medicine, University of Pisa
Via Savi 10, I-56126 Pisa, Italy
Regional Pharmacovigilance Center of Tuscany, Florence 50139, Italy
E-mail: irma.convertino@med.unipi.it

Table Supplementary Material (S) 1 Biologic target, therapeutic indications, date of first approval, safety profile of ICIs of interest approved by EMA and FDA: information commune to both agencies (HLGT) 3

Table S2 Date of approval, biologic target, therapeutic indications, safety profile of ICIs of interest approved by EMA and FDA: information specific to each agency (HLGT) 8

Table S3 Biologic target, therapeutic indications, date of approval, safety profile of ICIs of interest approved by EMA and FDA: information common to both agencies (HLT classification) 13

Table S4 Therapeutic indications, date of approval, safety profile of ICIs of interest approved by EMA and FDA: information specific to each agency (HLT classification) 19

File Text S1 24

Table S5 irAEs reported for each drug in the included studies conducted on SRSs (HLT classification) 27

Table S6 irAEs reported only as SOC in the included studies conducted on SRSs 39

File Text S2 48

Table S7 irAEs grade 3 or more for each drug in the included studies conducted on AHDs (HLGT classification) 50

Table S8 irAEs any grade reported for each drug in the included studies conducted on AHDs (HLT classification) 53

Table S9 irAEs grade 3 or more reported for each drug in the included studies conducted on AHDs (HLT classification) 59

Table S10 Preferred Reporting Items for Systematic reviews and Meta-Analyses extension for Scoping Reviews (PRISMA-ScR) Checklist 62

File Text S3 64

Table S1 Biologic target, therapeutic indications, date of first approval, safety profile of ICIs of interest approved by EMA and FDA: information commune to both agencies (HLGT)

ICI	Target	Therapeutic indications	Date of first approval		Immune-related adverse events expected (HLGT)	
			EMA	FDA	Associated with all ICIs	Associated with single ICI
Ipilimumab	CTLA-4	- Melanoma	07/2011	03/2011	- Adrenal gland disorders<br>- Central nervous system infections and inflammations<br>- Epidermal and dermal conditions<br>- Exocrine pancreas conditions<br>- Gastrointestinal inflammatory conditions<br>- Gastrointestinal motility and defaecation conditions<br>- Gastrointestinal signs and symptoms<br>- Glucose metabolism disorders (incl diabetes mellitus)<br>- Hepatic and hepatobiliary disorders<br>- Hypothalamus and pituitary gland disorders<br>- Immune disorders NEC<br>- Joint symptoms<br>- Lower respiratory tract disorders (excl obstructions and infection)<br>- Muscle disorders<br>- Myocardial disorders<br>- Nephropathies<br>- Neuromuscular disorders<br>- Ocular infections, irritations and inflammations<br>- Peripheral neuropathies<br>- Procedural related injuries and complications NEC<br>- Thyroid gland disorders	- Endocrine disorders of gonad function<br>- Pericardial disorders<br>- Demyelinating disorders<br>- Gastrointestinal investigation<br>- Ocular structural change, deposit and degeneration NEC<br>- Renal disorders (excl nephropathies)<br>- Connective tissue disorders (excl nephropathies)<br>- General system disorders NEC<br>- Joint disorders<br>- Infections- pathogen unspecified<br>- Diabetic complications<br>- Gastrointestinal haemorrhages NEC<br>- Vascular disorders NEC<br>- Ocular infections, irritations and inflammations<br>- Hearing disorders<br>- White blood cell disorders
Ipilimumab + Nivolumab	CTLA-4 + PD-1	- Melanoma<br>- Renal Cell Carcinoma<br>- NSCLC<br>- Malignant pleural mesothelioma<br>- Mismatch repair deficient or microsatellite instability-high colorectal cancer<br>- Esophageal squamous cell carcinoma	05/2016	10/2015		- Pericardial disorders<br>- Demyelinating disorders<br>- Gastrointestinal investigation<br>- Connective tissue disorders (excl nephropathies)<br>- Diabetic complications<br>- Gastrointestinal haemorrhages NEC<br>- Gastrointestinal ulcer and perforation<br>- Haemolyses and related conditions<br>- Hepatobiliary investigations

ICI	Target	Therapeutic indications	Date of first approval		Immune-related adverse events expected (HLGT)	
			EMA	FDA	Associated with all ICIs	Associated with single ICI
					- Vascular infections and inflammations	- Joint disorders<br>- Oral soft tissue conditions<br>- Parathyroid gland disorders<br>- Renal disorders (excl nephropathies)<br>- Vision disorders<br>- White blood cell disorders
Nivolumab	PD-1	- Melanoma (monotherapy)<br>- NSCLC (monotherapy)<br>- Renal cell carcinoma (in monotherapy and in combination with other therapies)<br>- Classical Hodgkin lymphoma (monotherapy)<br>- Squamous cell cancer of the head and neck (monotherapy)<br>- Urothelial carcinoma (in monotherapy and in combination with other therapies)<br>- Esophageal squamous cell carcinoma (in monotherapy and in combination with other therapies)<br>- Gastric, gastro-esophageal junction or esophageal adenocarcinoma (in	06/2015	12/2014		- Connective tissue disorders (excl nephropathies)<br>- Demyelinating disorders<br>- Diabetic complications<br>- Gastrointestinal haemorrhages NEC<br>- Gastrointestinal investigation<br>- General system disorders NEC<br>- Haemolyses and related conditions<br>- Hepatobiliary investigations<br>- Joint disorders<br>- Oral soft tissue conditions<br>- Parathyroid gland disorders<br>- Pericardial disorders<br>- Renal disorders (excl nephropathies)<br>- White blood cell disorders

ICI	Target	Therapeutic indications	Date of first approval		Immune-related adverse events expected (HLGT)	
			EMA	FDA	Associated with all ICIs	Associated with single ICI
		combination with other therapies)				
Pembrolizumab	PD-1	- Melanoma (monotherapy)<br>- NSCLC (in monotherapy and in combination with other therapies)<br>- Classical Hodgkin lymphoma (monotherapy)<br>- Urothelial carcinoma (in monotherapy and in combination with other therapies)<br>- Head and neck squamous cell carcinoma (in monotherapy or in combination with other therapies)<br>- Renal cell carcinoma (in monotherapy and in combination with other therapies)<br>- Microsatellite Instability-High or Mismatch Repair Deficient cancers (monotherapy)<br>- Microsatellite instability high or mismatch repair deficient colorectal (monotherapy)	07/2015	09/2014		- Bile duct disorders<br>- Connective tissue disorders (excl nephropathies)<br>- Diabetic complications<br>- Gastrointestinal haemorrhages NEC<br>- Gastrointestinal investigation<br>- Gastrointestinal ulcer and perforation<br>- Haemolyses and related conditions<br>- Hepatobiliary investigations<br>- Infections- pathogen unspecified<br>- Joint disorders<br>- Oral soft tissue conditions<br>- Parathyroid gland disorders<br>- Pericardial disorders<br>- Renal disorders (excl nephropathies)

ICI	Target	Therapeutic indications	Date of first approval		Immune-related adverse events expected (HLGT)	
			EMA	FDA	Associated with all ICIs	Associated with single ICI
		- Esophageal carcinoma (in combination with other therapies)<br>- Triple-negative breast cancer (in combination with other therapies)<br>- Endometrial carcinoma (in combination with other therapies)<br>- Cervical cancer (in combination with other therapies)<br>- Gastric or gastro-esophageal junction adenocarcinoma (in combination with other therapies)<br>- Biliary tract carcinoma (in combination with other therapies)				
Atezolizumab	PD-L1	- NSCLC (in monotherapy and in combination with other therapies)<br>- SCLC (in combination with other therapies)<br>- Hepatocellular carcinoma (in combination with other therapies)	09/2017	10/2016		- Connective tissue disorders (excl nephropathies)<br>- Diabetic complications<br>- Gastrointestinal haemorrhages NEC<br>- Gastrointestinal investigation<br>- Hepatobiliary investigations<br>- Oral soft tissue conditions<br>- Pericardial disorders
Durvalumab	PD-L1	- NSCLC (in monotherapy and in combination with other therapies)	09/2018	05/2017		- Anaemias nonhaemolytic and marrow depression<br>- Bile duct disorders<br>- Diabetic complications

ICI	Target	Therapeutic indications	Date of first approval		Immune-related adverse events expected (HLGT)	
			EMA	FDA	Associated with all ICIs	Associated with single ICI
		- SCLC (in combination with other therapies)<br>- Biliary tract cancer (in combination with other therapies)<br>- Hepatocellular carcinoma (in combination with other therapies)<br>- Endometrial cancer (in combination with other therapies)				- Gastrointestinal haemorrhages NEC<br>- Gastrointestinal investigation<br>- Gastrointestinal ulcer and perforation<br>- Haemolyses and related conditions<br>- Hepatobiliary investigations<br>- Injuries by physical agents<br>- Joint disorders<br>- Oral soft tissue conditions
Cemiplimab	PD-1	- Cutaneous Squamous Cell Carcinoma (monotherapy)<br>- Basal Cell Carcinoma (monotherapy)<br>- NSCLC (in monotherapy and in combination with other therapies)	06/2019	09/2018		- Connective tissue disorders (excl nephropathies)<br>- Diabetic complications<br>- Hepatobiliary investigations<br>- Joint disorders<br>- Oral soft tissue conditions<br>- Pericardial disorders<br>- Renal disorders (excl nephropathies)

ICI immune checkpoint inhibitor, PD-1 programmed cell death protein 1, PD-L1 programmed cell death protein ligand 1, CTLA-4 cytotoxic T-lymphocyte antigen 4, HLGT High Level Group Terms, EMA European Medicines Agency, FDA Food and Drug Administration, NSCLC non-small cell lung cancer, SCLC small cell lung cancer, NEC non elsewhere classified.

Table S2 Date of approval, biologic target, therapeutic indications, safety profile of ICIs of interest approved by EMA and FDA: information specific to each agency (HLGT)

ICI	Date of approval		Target	Therapeutic indications		Expected Immune-related adverse events (HLGT)	
	EMA	FDA		EMA	FDA	EMA	FDA
Ipilimumab	07/2011	03/2011	CTLA-4			- Bladder and bladder neck disorders (excl calculi)<br>- Diabetic complications<br>- Gastrointestinal ulcer and perforation<br>- Haemolyses and related conditions<br>- Hepatobiliary investigations<br>- Malabsorption conditions<br>- Ocular injuries<br>- Oral soft tissue conditions<br>- Parathyroid gland disorders	- Anaemias nonhaemolytic and marrow depression<br>- Haematopoietic neoplasms (excl leukaemias and lymphomas)<br>- Movement disorders (incl parkinsonism)<br>- Pericardial disorders<br>- Skin vascular abnormalities<br>- Vision disorders
Ipilimumab + Nivolumab	05/2016	10/2015	CTLA-4 + PD-1		- Hepatocellular Carcinoma	- Bladder and bladder neck disorders (excl calculi)<br>- Cranial nerve disorders (excl neoplasms)<br>- Infections- pathogen unspecified<br>- Malabsorption conditions<br>- Ocular infections, irritations and inflammations	- Anaemias nonhaemolytic and marrow depression<br>- General system disorders NEC<br>- Ocular injuries<br>- Ocular structural change, deposit and degeneration NEC
Nivolumab	06/2015	12/2014	PD-1		- Microsatellite instability-high or mismatch repair deficient metastatic	- Bladder and bladder neck disorders (excl calculi)<br>- Cranial nerve disorders (excl neoplasms)<br>- Infections- pathogen unspecified<br>- Malabsorption conditions<br>- Vision disorders	- Anaemias nonhaemolytic and marrow depression<br>- Bile duct disorders<br>- Gastrointestinal ulcer and perforation

					colorectal cancer (monotherapy)		- Haematopoietic neoplasms (excl leukaemias and lymphomas)<br>- Ocular injuries<br>- Ocular structural change, deposit and degeneration NEC
Pembrolizumab	07/2015	09/2014	PD-1		- Malignant Pleural Mesothelioma (in combination with other therapies)<br>- Primary Mediastinal Large B-Cell Lymphoma (monotherapy)<br>- Esophageal carcinoma (monotherapy)<br>- Endometrial carcinoma (monotherapy)<br>- Cervical cancer (monotherapy)<br>- Hepatocellular Carcinoma (monotherapy)	- Bladder and bladder neck disorders (excl calculi)<br>- Malabsorption conditions<br>- Vision disorders<br>- White blood cell disorders	- Anaemias nonhaemolytic and marrow depression<br>- Demyelinating disorders<br>- General system disorders NEC<br>- Haematopoietic neoplasms (excl leukaemias and lymphomas)<br>- Ocular injuries<br>- Ocular structural change, deposit and degeneration NEC

					- Merkel Cell Carcinoma (monotherapy)<br>- Tumor Mutational Burden-High (TMB-H) Cancer (monotherapy) (monotherapy)<br>- Cutaneous Squamous Cell Carcinoma (monotherapy)		
Atezolizumab	09/2017	10/2016	PD-L1	- Urothelial carcinoma (in monotherapy)<br>- Triple-negative breast cancer (in combination with other therapies)	- Alveolar Soft Part Sarcoma (in monotherapy)<br>- Melanoma (in combination with other therapies)<br>- Central nervous system and spinal infections<br>- Gastrointestinal ulcers	- Bladder and bladder neck disorders (excl calculi)<br>- Cranial nerve disorders (excl neoplasms)<br>- Gastrointestinal ulcer and perforation<br>- Infections- pathogen unspecified<br>- Injuries by physical agents<br>- Malabsorption conditions<br>- Vision disorders	- Anaemias nonhaemolytic and marrow depression<br>- Demyelinating disorders<br>- General system disorders NEC<br>- Haematopoietic neoplasms (excl leukaemias and lymphomas)<br>- Haemolyses and related conditions<br>- Joint disorders<br>- Ocular injuries<br>- Ocular structural change, deposit and degeneration NEC<br>- Parathyroid gland disorders

					and perforation, site unspecified<br>- Glomerulonephritis and nephrotic syndrome		- Renal disorders (excl nephropathies)
Durvalumab	09/2018	05/2017	PD-L1	- Hepatocellular carcinoma (in monotherapy)	- SCLC (in monotherapy)	- Bladder and bladder neck disorders (excl calculi)<br>- Malabsorption conditions<br>- Vision disorders	- Connective tissue disorders (excl nephropathies)<br>- Demyelinating disorders<br>- General system disorders NEC<br>- Haematopoietic neoplasms (excl leukaemias and lymphomas)<br>- Infections-pathogen unspecified<br>- Ocular injuries<br>- Ocular structural change, deposit and degeneration NEC<br>- Parathyroid gland disorders<br>- Renal disorders (excl nephropathies)
Cemiplimab	06/2019	09/2018	PD-1	- Cervical Cancer (in monotherapy)		- Bladder and bladder neck disorders (excl calculi)<br>- Infections- pathogen unspecified<br>- Malabsorption conditions<br>- Vision disorders	- Anaemias nonhaemolytic and marrow depression<br>- Demyelinating disorders<br>- Gastrointestinal haemorrhages NEC<br>- Gastrointestinal investigation<br>- General system disorders NEC

							- Haematopoietic neoplasms (excl leukaemias and lymphomas)<br>- Haemolyses and related conditions<br>- Ocular injuries<br>- Ocular structural change, deposit and degeneration NEC<br>- Parathyroid gland disorders

ICIs immune checkpoint inhibitors, EMA European Medicines Agency, FDA Food and Drug Administration, HLGT High Level Group Terms, PD-1 programmed cell death protein 1, PD-L1 programmed cell death protein ligand 1, CTLA-4 cytotoxic T-lymphocyte antigen 4

Table S3 Biologic target, therapeutic indications, date of approval, safety profile of ICIs of interest approved by EMA and FDA: information common to both agencies (HLT classification)

ICI	Target	Therapeutic indications	Date of first approval		Immune-related adverse events expected (HLT)	
			FDA	EMA	Associated with all ICIs	Associated with single ICI
Ipilimumab	CTLA-4	- Melanoma	03/2011	07/2011	- Acute and chronic thyroiditis<br>- Acute polyneuropathies<br>- Adrenal cortical hypofunctions<br>- Bullous conditions<br>- Colitis<br>- Dermatitis and eczema<br>- Diabetes mellitus (incl subtypes)<br>- Diarrhoea<br>- Encephalitis NEC<br>- Gastrointestinal and abdominal pains (excl oral and throat)<br>- Hepatocellular damage and hepatitis NEC<br>- Hypothalamic and pituitary disorders NEC<br>- Immune disorders NEC<br>- Infections, irritations and inflammations<br>- Joint related sign and symptoms<br>- Lower respiratory tract inflammatory and immunologic conditions<br>- Meningitis NEC<br>- Muscle infections and inflammations<br>- Muscle pains<br>- Nephritis NEC<br>- Neuromuscular junction dysfunction<br>- Non-site specific procedural complications<br>- Noninfectious myocarditis<br>- Peripheral neuropathies NEC	- Acute and chronic pancreatitis<br>- Acute and chronic sarcoidosis<br>- Anterior pituitary hypofunction<br>- Arterial infections and inflammations<br>- Arthropathies NEC<br>- Connective tissue disorders NEC<br>- Demyelinating disorders NEC<br>- Dermatitis ascribed to specific agent<br>- Digestive enzymes<br>- Endocrine abnormalities of gonad function NEC<br>- Eosinophilic disorders<br>- Eye and eyelid infections<br>- Gastritis (excl infective)<br>- Gastrointestinal spastic and hypermotility disorders<br>- Hearing losses<br>- Immune and associated conditions NEC<br>- Inflammations<br>- Lid, lash and lacrimal infections, irritations and inflammations<br>- Muscle weakness conditions<br>- Myelitis (incl infective)<br>- Myopathies<br>- Non-site specific gastrointestinal haemorrhages<br>- Non-site specific vascular disorders NEC<br>- Psoriatic conditions<br>- Renal failure and impairment<br>- Retinal structural change, deposit and degeneration

ICI	Target	Therapeutic indications	Date of first approval		Immune-related adverse events expected (HLT)	
			FDA	EMA	Associated with all ICIs	Associated with single ICI
					- Pruritus NEC<br>- Thyroid hyperfunction disorders<br>- Vasculitides NEC	- Scleral infections, irritations and inflammations<br>- Transplant rejection
Ipilimumab + Nivolumab	CTLA-4 + PD-1	- Melanoma<br>- Renal Cell Carcinoma<br>- Non-small Cell lung Cancer<br>- Malignant pleural mesothelioma<br>- Mismatch repair deficient or microsatellite instability-high colorectal cancer<br>- Esophageal squamous cell carcinoma	10/2015	05/2016		- Acute and chronic pancreatitis<br>- Acute and chronic sarcoidosis<br>- Anaemias haemolytic NEC<br>- Anterior pituitary hypofunction<br>- Arthropathies NEC<br>- Connective tissue disorders NEC<br>- Demyelinating disorders<br>- Diabetic complications NEC<br>- Digestive enzymes<br>- Eosinophilic disorders<br>- Exfoliative conditions<br>- Gastritis (excl infective)<br>- Gastrointestinal inflammatory disorders NEC<br>- Gastrointestinal spastic and hypermotility disorders<br>- Gastrointestinal ulcers and perforation, site unspecified<br>- Hepatobiliary function diagnostic procedures<br>- Hypoparathyroid disorders<br>- Immune and associated conditions NEC<br>- Muscle weakness conditions<br>- Myelitis (incl infective)<br>- Myopathies<br>- Non-site specific gastrointestinal haemorrhages<br>- Psoriatic conditions<br>- Renal failure and impairment<br>- Stomatitis and ulceration<br>- Thyroid disorders NEC<br>- Transplant rejection

ICI	Target	Therapeutic indications	Date of first approval		Immune-related adverse events expected (HLT)	
			FDA	EMA	Associated with all ICIs	Associated with single ICI
Nivolumab	PD-1	- Melanoma (monotherapy)<br>- Non-small cell lung cancer (monotherapy)<br>- Renal cell carcinoma (in monotherapy and in combination with other therapies)<br>- Classical Hodgkin lymphoma (monotherapy)<br>- Squamous cell cancer of the head and neck (monotherapy)<br>- Urothelial carcinoma (in monotherapy and in combination with other therapies)<br>- Esophageal squamous cell carcinoma (in monotherapy and in combination with other therapies)<br>- Gastric, gastro-esophageal junction or esophageal adenocarcinoma (in combination with other therapies)	12/2014	06/2015		- Acute and chronic pancreatitis<br>- Acute and chronic sarcoidosis<br>- Anaemias haemolytic NEC<br>- Anterior pituitary hypofunction<br>- Arthropathies NEC<br>- Connective tissue disorders NEC<br>- Demyelinating disorders<br>- Diabetic complications NEC<br>- Digestive enzymes<br>- Eosinophilic disorders<br>- Exfoliative conditions<br>- Gastritis (excl infective)<br>- Gastrointestinal inflammatory disorders NEC<br>- Gastrointestinal spastic and hypermotility disorders<br>- General signs and symptoms NEC<br>- Hepatobiliary function diagnostic procedures<br>- Hypoparathyroid disorders<br>- Immune and associated conditions NEC<br>- Muscle weakness conditions<br>- Myelitis (incl infective)<br>- Myopathies<br>- Non-site specific gastrointestinal haemorrhages<br>- Psoriatic conditions<br>- Renal failure and impairment<br>- Stomatitis and ulceration<br>- Thyroid disorders NEC<br>- Transplant rejection
Pembrolizu mab	PD-1	- Melanoma (monotherapy)<br>- NSCLC (in monotherapy and in combination with other therapies)	09/2014	07/2015		- Acute and chronic pancreatitis<br>- Acute and chronic sarcoidosis<br>- Anaemias haemolytic NEC<br>- Anterior pituitary hypofunction

ICI	Target	Therapeutic indications	Date of first approval		Immune-related adverse events expected (HLT)	
			FDA	EMA	Associated with all ICIs	Associated with single ICI
		- Classical Hodgkin lymphoma (monotherapy)<br>- Urothelial carcinoma (in monotherapy and in combination with other therapies)<br>- Head and neck squamous cell carcinoma (in monotherapy or in combination with other therapies)<br>- Renal cell carcinoma (in monotherapy and in combination with other therapies)<br>- Microsatellite Instability-High or Mismatch Repair Deficient cancers (monotherapy)<br>- Microsatellite instability high or mismatch repair deficient colorectal (monotherapy)<br>- Esophageal carcinoma (in combination with other therapies)<br>- Triple-negative breast cancer (in combination with other therapies)<br>- Endometrial carcinoma (in combination with other therapies)<br>- Cervical cancer (in combination with other therapies)<br>- Gastric or gastro-esophageal junction adenocarcinoma (in combination with other therapies)<br>- Biliary tract carcinoma (in combination with other therapies)				- Arthropathies NEC<br>- Bile duct infections and inflammations<br>- Connective tissue disorders NEC<br>- Diabetic complications NEC<br>- Digestive enzymes<br>- Exfoliative conditions<br>- Gastritis (excl infective)<br>- Gastrointestinal ulcers and perforation, site unspecified<br>- Hepatobiliary function diagnostic procedures<br>- Hypoparathyroid disorders<br>- Immune and associated conditions NEC<br>- Myelitis (incl infective)<br>- Myopathies<br>- Non-site specific gastrointestinal haemorrhages<br>- Pancreatic disorders NEC<br>- Psoriatic conditions<br>- Renal failure and impairment<br>- Stomatitis and ulceration<br>- Thyroid disorders NEC<br>- Transplant rejection

ICI	Target	Therapeutic indications	Date of first approval		Immune-related adverse events expected (HLT)	
			FDA	EMA	Associated with all ICIs	Associated with single ICI
Atezolizumab	PD-L1	- NSCLC (in monotherapy and in combination with other therapies)<br>- SCLC (in combination with other therapies)<br>- Hepatocellular carcinoma (in combination with other therapies)	10/2016	09/2017		- Acute and chronic pancreatitis<br>- Anterior pituitary hypofunction<br>- Connective tissue disorders NEC<br>- Dermatitis ascribed to specific agent<br>- Diabetic complications NEC<br>- Digestive enzymes<br>- Exfoliative conditions<br>- Gastrointestinal spastic and hypermotility disorders<br>- Hepatobiliary function diagnostic procedures<br>- Immune and associated conditions NEC<br>- Muscle weakness conditions<br>- Myelitis (incl infective)<br>- Myopathies<br>- Non-site specific gastrointestinal haemorrhages<br>- Psoriatic conditions<br>- Stomatitis and ulceration
Durvalumab	PD-L1	- NSCLC (in monotherapy and in combination with other therapies)<br>- SCLC (in combination with other therapies)<br>- Biliary tract cancer (in combination with other therapies)<br>- Hepatocellular carcinoma (in combination with other therapies)<br>- Endometrial cancer (in combination with other therapies)	05/2017	09/2018		- Acute and chronic pancreatitis<br>- Anaemias haemolytic NEC<br>- Anterior pituitary hypofunction<br>- Arthropathies NEC<br>- Bile duct infections and inflammations<br>- Diabetic complications NEC<br>- Digestive enzymes<br>- Gastrointestinal spastic and hypermotility disorders<br>- Gastrointestinal ulcers and perforation, site unspecified<br>- Hepatobiliary function diagnostic procedures<br>- Marrow depression and hypoplastic anaemias

ICI	Target	Therapeutic indications	Date of first approval		Immune-related adverse events expected (HLT)	
			FDA	EMA	Associated with all ICIs	Associated with single ICI
						- Muscle weakness conditions<br>- Myelitis (incl infective)<br>- Myopathies<br>- Non-site specific gastrointestinal haemorrhages<br>- Radiation injuries<br>- Stomatitis and ulceration
Cemiplimab	PD-1	- Cutaneous Squamous Cell Carcinoma (monotherapy)<br>- Basal Cell Carcinoma (monotherapy)<br>- NSCLC (in monotherapy and in combination with other therapies)	09/2018	06/2019		- Arthropathies NEC<br>- Connective tissue disorders NEC<br>- Diabetic complications NEC<br>- Exfoliative conditions<br>- Gastrointestinal spastic and hypermotility disorders<br>- Hepatobiliary function diagnostic procedures<br>- Immune and associated conditions NEC<br>- Muscle weakness conditions<br>- Psoriatic conditions<br>- Renal failure and impairment<br>- Stomatitis and ulceration<br>- Thyroid disorders NEC<br>- Transplant rejection

ICIs immune checkpoint inhibitors; EMA European Medicines Agency; FDA Food and Drug Administration; HLT High Level Terms; PD-1 programmed cell death protein 1; PD-L1 programmed cell death protein ligand 1; CTLA-4 cytotoxic T-lymphocyte antigen 4; NEC not elsewhere classified

Table S4 Therapeutic indications, date of approval, safety profile of ICIs of interest approved by EMA and FDA: information specific to each agency (HLT classification)

ICI	Date of approval		Target	Therapeutic indications		Expected Immune-related adverse events (HLT)	
	FDA	EMA		EMA	FDA	EMA	FDA
Ipilimumab	03/2011	07/2011	CTLA-4			- Anaemias haemolytic NEC<br>- Bladder infections and inflammations<br>- Central nervous system and spinal infections<br>- Central nervous system inflammatory disorders NEC<br>- Diabetic complications NEC<br>- Gastrointestinal ulcers and perforation, site unspecified<br>- General signs and symptoms NEC<br>- Glomerulonephritis and nephrotic syndrome<br>- Hepatobiliary function diagnostic procedures<br>- Hypoparathyroid disorders<br>- Intestinal haemorrhages<br>- Malabsorption syndromes<br>- Ocular injuries NEC<br>- Pancreatic disorders NEC<br>- Stomatitis and ulceration<br>- Visual impairment and blindness (excl colour blindness)	- Exfoliate conditions<br>- Gastrointestinal inflammatory disorders NEC<br>- Lymphoproliferative disorders NEC (excl leukaemias and lymphomas)<br>- Marrow depression and hypoplastic anaemia
Ipilimumab + Nivolumab	10/2015	05/2016	CTLA-4 + PD-1		- Hepatocellular Carcinoma	- Bladder infections and inflammations	- General signs and symptoms NEC<br>- Inflammations

ICI	Date of approval		Target	Therapeutic indications		Expected Immune-related adverse events (HLT)	
	FDA	EMA		EMA	FDA	EMA	FDA
						- Central nervous system and spinal infections<br>- Eye and eyelid infections<br>- Facial cranial nerve disorders<br>- Malabsorption syndromes<br>- Pancreatic disorders NEC<br>- Scleral infections, irritations and inflammations	- Intestinal haemorrhages<br>- Lymphoproliferative disorders NEC (excl leukaemias and lymphomas)<br>- Marrow depression and hypoplastic anaemias<br>- Ocular injuries NEC<br>- Retinal structural change, deposit and degeneration<br>- Visual impairment and blindness (excl colour blindness)
Nivolumab	12/2014	06/2015	PD-1		- Microsatellite instability-high or mismatch repair deficient metastatic colorectal cancer (monotherapy)	- Bladder infections and inflammations<br>- Central nervous system and spinal infections<br>- Facial cranial nerve disorders<br>- Malabsorption syndromes<br>- Pancreatic disorders NEC	- Bile duct infections and inflammations<br>- Dermatitis ascribed to specific agent<br>- Gastrointestinal ulcers and perforation, site unspecified<br>- Inflammations<br>- Intestinal haemorrhages<br>- Lymphoproliferative disorders NEC (excl leukaemias and lymphomas)<br>- Marrow depression and hypoplastic anaemias<br>- Ocular injuries NEC<br>- Retinal structural change, deposit and degeneration<br>- Visual impairment and blindness (excl colour blindness)
Pembrolizumab	09/2014	07/2015	PD-1		- Malignant Pleural Mesothelioma (in combination with other therapies)	- Bladder infections and inflammations<br>- Malabsorption syndromes	- Demyelinating disorders<br>- Dermatitis ascribed to specific agent<br>- Eye and eyelid infections

ICI	Date of approval		Target	Therapeutic indications		Expected Immune-related adverse events (HLT)	
	FDA	EMA		EMA	FDA	EMA	FDA
					- Primary Mediastinal Large B-Cell Lymphoma (monotherapy)<br>- Esophageal carcinoma (monotherapy)<br>- Endometrial carcinoma (monotherapy)<br>- Cervical cancer (monotherapy)<br>- Hepatocellular Carcinoma (monotherapy)<br>- Merkel Cell Carcinoma (monotherapy)<br>- Tumor Mutational Burden-High (TMB-H) Cancer (monotherapy) (monotherapy)<br>- Cutaneous Squamous Cell Carcinoma (monotherapy)	- Central nervous system and spinal infections<br>- Chronic polyneuropathies<br>- Gastrointestinal spastic and hypermotility disorders<br>- Glomerulonephritis and nephrotic syndrome<br>- Eosinophilic disorders	- Gastrointestinal inflammatory disorders NEC<br>- Inflammations<br>- Intestinal haemorrhages<br>- Lymphoproliferative disorders NEC (excl leukaemias and lymphomas)<br>- Marrow depression and hypoplastic anaemias<br>- Muscle weakness conditions<br>- Ocular injuries NEC<br>- Retinal structural change, deposit and degeneration<br>- Visual impairment and blindness (excl colour blindness)
Atezolizumab	10/2016	09/2017	PD-L1	- Urothelial carcinoma (in monotherapy)<br>- Triple-negative breast cancer (in combination with other therapies)	- Alveolar Soft Part Sarcoma (in monotherapy)<br>- Melanoma (in combination with other therapies)<br>- Central nervous system and spinal infections<br>- Gastrointestinal ulcers and	- Bladder infections and inflammations<br>- Chronic polyneuropathies<br>- Facial cranial nerve disorders<br>- Malabsorption syndromes<br>- Pancreatic disorders NEC<br>- Radiation injuries	- Acute and chronic sarcoidosis<br>- Anaemias haemolytic NEC<br>- Arthropathies NEC<br>- Corneal infections, oedemas and inflammations<br>- Demyelinating disorders<br>- Gastritis (excl infective)<br>- Gastrointestinal inflammatory disorders NEC<br>- Hypoparathyroid disorders<br>- Inflammations

ICI	Date of approval		Target	Therapeutic indications		Expected Immune-related adverse events (HLT)	
	FDA	EMA		EMA	FDA	EMA	FDA
					perforation, site unspecified<br>- Glomerulonephritis and nephrotic syndrome		- Intestinal haemorrhages<br>- Lymphoproliferative disorders NEC (excl leukaemias and lymphomas)<br>- Marrow depression and hypoplastic anaemias<br>- Ocular injuries NEC<br>- Renal failure and impairment<br>- Retinal structural change, deposit and degeneration<br>- Thyroid disorders NEC<br>- Transplant rejection<br>- Visual impairment and blindness (excl colour blindness)
Durvalumab	05/2017	09/2018	PD-L1	- Hepatocellular carcinoma (in monotherapy)	- SCLC (in monotherapy)	- Bladder infections and inflammations<br>- Glomerulonephritis and nephrotic syndrome<br>- Malabsorption syndromes<br>- Pancreatic disorders NEC<br>- Psoriatic conditions	- Acute and chronic sarcoidosis<br>- Central nervous system and spinal infections<br>- Connective tissue disorders NEC<br>- Corneal infections, oedemas and inflammations<br>- Gastritis (excl infective)<br>- Gastrointestinal inflammatory disorders NEC<br>- Hypoparathyroid disorders<br>- Immune and associated conditions NEC<br>- Inflammations<br>- Lymphoproliferative disorders NEC (excl leukaemias and lymphomas)<br>- Ocular injuries NEC<br>- Renal failure and impairment<br>- Retinal structural change, deposit and degeneration<br>- Thyroid disorders NEC

ICI	Date of approval		Target	Therapeutic indications		Expected Immune-related adverse events (HLT)	
	FDA	EMA		EMA	FDA	EMA	FDA
							- Transplant rejection<br>- Visual impairment and blindness (excl colour blindness)
Cemiplimab	09/2018	06/2019	PD-1	- Cervical Cancer (in monotherapy)		- Bladder infections and inflammations<br>- Central nervous system and spinal infections<br>- Central nervous system inflammatory disorders NEC<br>- Chronic polyneuropathies<br>- Corneal infections, oedemas and inflammations<br>- Malabsorption syndromes<br>- Pancreatic disorders NEC	- Acute and chronic sarcoidosis<br>- Anaemias haemolytic NEC<br>- Anterior pituitary hypofunction<br>- Gastrointestinal inflammatory disorders NEC<br>- Hypoparathyroid disorders<br>- Inflammations<br>- Lymphoproliferative disorders NEC (excl leukaemias and lymphomas)<br>- Marrow depression and hypoplastic anaemias<br>- Myopathies<br>- Non-site specific gastrointestinal haemorrhages<br>- Ocular injuries NEC<br>- Retinal structural change, deposit and degeneration<br>- Visual impairment and blindness (excl colour blindness)

ICIs immune checkpoint inhibitors; EMA European Medicines Agency; FDA Food and Drug Administration; HLT High Level Terms; PD-1 programmed cell death protein 1; PD-L1 programmed cell death protein ligand 1; CTLA-4 cytotoxic T-lymphocyte antigen 4;NEC not elsewhere classified

File Text S1

Observational studies included in this review concerning SRSs (Table 3) are conducted on FAERS (n=14, 64%)[34,36,39,42,43,49-56-58], VigiBase (n=6, 27%)[32,33,35,38,40,41], EudraVigilance (n=1, 5%)[37] and JADER (n=1, 5%)[57]. Lung cancer is reported in 11 (50%) studies[32,35,36,39,40-42,50,51,54,56], followed by melanoma (n=10, 46%)[33,40-42,49,50,52,55,56,58], GI cancers (n=10, 46%)[32,35,36,39,40,42,49-52], and renal cancer (n=10, 46%)[32,35,39,41,42,49,50,51,58,]. Pembrolizumab[32,34-43,49,50-53,54,56-58,] and nivolumab[32,4-39,41,42,43,59-53,54-58,] are the drugs most reported (n=20, 91% each), ipilimumab 15 (68%)[32,33,35-39,42,43,49,51-53,55,58], atezolizumab 16 (73%)[32,34-37,39,41-43,49,50-54,57], durvalumab 14 (64%)[32,35,36,39,41-43,50-54,57], cemiplimab 9 (41%)[36-39,42,43,51,53,54], nivolumab + ipilimumab 7 (32%)[32,35-37,39,51,55], pembrolizumab + ipilimumab 5 (23%)[32,35,36,39,51] (Table 3). The time to onset of irAEs was reported in 4 (18%) studies[49,32,36,58], and the correspondence time to onset-drug-specific irAE [49], and the correspondence time to onset-drug[58], was assessed in 1 study each, with a mean value of 101 days (68-134) and 113 days (69-180), respectively. Two studies[32,49] reported the correspondence time to onset-specific irAE, with a mean value of 62 days (45-92). The duration of irAEs was documented in 2 (9%) studies[49,58], with a mean value of 26 days, while the management of irAEs was described in 3 (14%) studies[37,40,49], with "discontinuation" as the most reported management strategy (9%). Comorbidities were documented in 3 (14%) studies[37,49,54], and concomitant therapies were displayed in 3 (14%) studies[40,49,58], with multiple sclerosis treatment and atorvastatin as concomitant drugs. The outcome of irAEs was described in 12 (55%) studies[32,35-37,39,40,49-52,56,58] in particular death was reported in 11 (92%) studies [32,35,36,39,40,49-52,56,58] , hospitalization in 5 (42%)[36,49-51,58], disability in 3 (25%)[36,49,58], life-threatening outcome in 3 (25%)[36,51,58], required intervention to prevent permanent impairment/damage in 2 (17%)[36,58], recovered/resolved in 3 (25%)[32,37,49], recovering/resolving in 1 (8%)[37], not recovered/not resolved in 2 (17%)[32,37], resolved with sequelae in 1 (8%)[37]. Survival analysis, as well as data on dechallenge and rechallenge, were not reported in any of the studies. Additional information of demographical and clinical characteristics of included studies conducted

on SRSs can be found in Table 3. Out of twenty studies, sixteen and seventeen ones reported irAEs specific to nivolumab[24-39,43,49-55,57,58] and pembrolizumab[34-40,42,43,49-54,57,48], respectively. 14/15 studies documented irAEs specific for ipilimumab[33,35-48,42,43,48,51-53,55,58], 14/16 studies for atezolizumab[34-37,39,42,43,49-54,57], 10/14 for durvalumab[36,39,43,49-54,57], 9/9 for cemiplimab[36-39,42,43,51,53,54], 6/7 for nivolumab + ipilimumab[35-37,39,51,55], 4/5 for pembrolizumab + ipilimumab[35,36,39,51], and 1/1 for cemiplimab + ipilimumab[39]. The irAEs most frequently reported (Table 4) for nivolumab overall and in lung cancer patients is "Lower respiratory tract disorders (excl obstruction and infection)", while "Hepatobiliary investigations" is the irAE most reported for melanoma patients. "Gastrointestinal inflammatory conditions" is the irAE described for nivolumab in lung, pleura, thymus and heart cancer, digestive system cancer and skin cancer patients. For ipilimumab overall, melanoma, lung, pleura, thymus and heart cancer, digestive system cancer and skin cancer patients, the irAE most reported is "Gastrointestinal inflammatory conditions. As for nivolumab, "Gastrointestinal inflammatory conditions" is the irAE reported for lung, pleura, thymus and heart cancer, digestive system cancer and skin cancer patients. "Lower respiratory tract disorders (excl obstruction and infection)" is the term most irAE described for pembrolizumab overall and in NSCLC patients, while "Muscle disorders" for melanoma and lung cancer patients, and "Gastrointestinal inflammatory conditions" for skin cancer, lung, pleura, thymus and heart cancer and digestive system patients. Atezolizumab has "Lower respiratory tract disorders (excl obstruction and infection)" as the irAE most reported overall and for lung cancer (NSCLC incl) patients. "Lower respiratory tract disorders (excl obstruction and infection)" is also the irAE most reported for durvalumab overall. "Gastrointestinal inflammatory conditions" is the irAE reported for lung, pleura, thymus and heart cancer, digestive system cancer and skin cancer patients treated with atezolizumab, and for lung, pleura, thymus and heart cancer and digestive systems patients treated with durvalumab. Cemiplimab has "Immune disorders NEC" as the irAE most documented overall, and "gastrointestinal inflammatory conditions" for cemiplimab in lung, pleura, thymus and heart cancer and skin cancer patients. Both nivolumab + ipilimumab overall, digestive system cancer, skin cancer and

pembrolizumab + ipilimumab overall have “Gastrointestinal inflammatory conditions” as the irAE most reported, while melanoma patients treated with nivolumab + ipilimumab has “Gastrointestinal motility and defaecation conditions” as the most displayed one. Only one study reported “Immune disorders NEC” for patients treated with cemiplimab + ipilimumab. It was not possible to retrieve HLGT for pembrolizumab + nivolumab and ipilimumab + pembrolizumab + nivolumab. Additional information of irAEs reported for each drug is provided in Table 4.

Table S5 irAEs reported for each drug in the included studies conducted on SRSs (HLT classification)

Drug	Tumor	Number of studies reporting the drug for the tumor and irAE	Number of irAE	HLT
Nivolumab	Overall	14	1703	Lower respiratory tract inflammatory and immunologic conditions
			917	Colitis (excl infective)
			802	Pleural conditions NEC
			757	Peripheral neuropathies NEC
			507	Hypothalamic and pituitary disorders NEC/Anterior pituitary hypofunction
			389	Infection NEC
			348	Central nervous system and spinal infections
			327	Parenchymal lung disorders NEC
			324	Muscle infections and inflammations
			269	Gastrointestinal inflammatory disorders NEC
			235	General nutritional disorders NEC
			220	Diabetes mellitus (incl subtypes)
			200	Adrenal cortical hypofunctions
			191	Neuromuscular junction dysfunction
			186	Peritoneal and retroperitoneal disorders
			156	Central nervous system haemorrhages and cerebrovascular accidents
			115	Thrombocytopenias
			111	Coughing and associated symptoms
			109	Bile duct infections and inflammations
			93	Encephalopathies NEC
			92	Immune and associated conditions NEC
			83	Gastrointestinal stenosis and obstruction NEC
			67	Leukaemias acute myeloid
			64	Eyelid movement disorders
			58	Hepatobiliary function diagnostic procedures

Drug	Tumor	Number of studies reporting the drug for the tumor and irAE	Number of irAE	HLT
			50	Acute polyneuropathies
			49	Vasculitides NEC
			42	Thyroid hypofunction disorders
			41	Acute and chronic sarcoidosis
			39	Demyelinating disorders NEC
			38	Diarrhoea (excl infective)
			34	Transplant rejections
			29	Pruritus NEC
			27	Hepatocellular damage and hepatitis NEC
			21	Appetite disorders
			17	Non-site specific procedural complications
			17	Psoriatic conditions
			15	Hepatic enzymes and function abnormalities
			14	Tissue enzyme analyses NEC
			11	Respiratory tract disorders NEC
			11	Sepsis, bacteraemia, viraemia and fungaemia NEC
			10	Renal failure and impairment
			10	Iris and uveal tract infections, irritations and inflammations
			9	Nephritis NEC
			9	Multiple sclerosis acute and progressive
			9	Adrenal cortical hypofunctions/Hepatic and hepatobiliary disorders NEC/Diabetic complications NEC
	Melanoma	1	58	Hepatobiliary function diagnostic procedures
			42	Thyroid hypofunction disorders
			38	Diarrhoea (excl infective)
			29	Pruritus NEC
			21	Appetite disorders

Drug	Tumor	Number of studies reporting the drug for the tumor and irAE	Number of irAE	HLT
			18	Colitis (excl infective)
			16	Lower respiratory tract inflammatory and immunologic conditions
			15	Hepatic enzymes and function abnormalities
			15	Parenchymal lung disorders NEC
			14	Tissue enzyme analyses NEC
			11	Leukaemias acute myeloid
			11	Respiratory tract disorders NEC
			11	Sepsis, bacteraemia, viraemia and fungaemia NEC
	Lung Cancer	1	312	Parenchymal lung disorders NEC
	Lung, pleura, thymus and heart	1	386	Colitis (excl infective)
	Digestive system	1	50	Colitis (excl infective)
	Skin	1	154	Colitis (excl infective)
Ipilimumab	Overall	13	2029	Colitis (excl infective)
			741	Transplant rejections
			612	Hypothalamic and pituitary disorders NEC/Anterior pituitary hypofunction
			388	Diarrhoea (excl infective)
			336	Peripheral neuropathies NEC
			191	Central nervous system and spinal infections
			181	Hepatocellular damage and hepatitis NEC
			176	Rash, eruptions and exanthems NEC
			157	Lower respiratory tract inflammatory and immunologic conditions
			139	Asthenic conditions
			125	Hypothalamic and pituitary disorders NEC
			117	Muscle infections and inflammations
			107	Febrile disorders
			106	Adrenal cortical hypofunctions

Drug	Tumor	Number of studies reporting the drug for the tumor and irAE	Number of irAE	HLT
			100	Infections NEC
			99	Nausea and vomiting symptoms
			79	Pruritus NEC
			78	Total fluid volume decreased
			78	Coagulopathies
			74	Appetite disorders
			68	Sepsis, bacteriemia, viraemia and fungaemia NEC
			67	Gastrointestinal and abdominal pains (excl oral and throat)
			63	Neuromuscular junction dysfunction
			59	Peritoneal and retroperitoneal disorders
			56	Gastrointestinal inflammatory disorders NEC
			55	Pericardial disorders NEC
			53	Physical examination procedures and organ system status
			53	Lymphatic system disorders NEC
			47	Encephalopathies NEC
			46	Anaemias NEC
			44	Acute polyneuropathies
			42	Adrenal cortical hypofunctions
			39	Cholestasis and jaundice
			38	Optic nerve disorders NEC
			36	Non-site specific procedure complications
			35	Sodium imbalance
			35	Intestinal ulcers and perforation NEC
			34	Thyroid hypofunction disorders
			34	Non-site specific embolism and thrombosis
			33	Cholecystitis and cholelithiasis
			30	Bile duct infections and inflammations

Drug	Tumor	Number of studies reporting the drug for the tumor and irAE	Number of irAE	HLT
			27	Nephritis NEC
			24	Demyelinating disorders NEC
			21	Vasculitides NEC
			19	Thrombocytopenias
			12	Psoriatic conditions
			10	Noninfectious myocarditis
			8	Immune and associated conditions NEC
			5	Multiple sclerosis acute and progressive
	Skin	1	870	Colitis (excl infective)
	Lung, pleura, thymus and heart	1	26	Colitis (excl infective)
	Digestive system	1	1	Colitis (excl infective)
	Melanoma	2	1031	Colitis (excl infective)
			388	Diarrhoea (excl infective)
			176	Rash, eruptions and exanthems NEC
			139	Asthenic conditions
			125	Hypothalamic and pituitary disorders NEC
			107	Febrile disorders
			99	Nausea and vomiting symptoms
			84	Hepatocellular damage and hepatitis NEC
			79	Pruritus NEC
			78	Total fluid volume decreased
			74	Appetite disorders
			71	Lower respiratory tract inflammatory and immunologic conditions
			67	Gastrointestinal and abdominal pains (excl oral and throat)
			56	Gastrointestinal inflammatory disorders NEC
			53	Physical examination procedures and organ system status

Drug	Tumor	Number of studies reporting the drug for the tumor and irAE	Number of irAE	HLT
			46	Anaemias NEC
			42	Adrenal cortical hypofunctions
			38	Sepsis, bacteriemia, viraemia and fungaemia NEC
			35	Sodium imbalance
			35	Intestinal ulcers and perforation NEC
			34	Thyroid hypofunction disorders
Atezolizumab	Overall	12	701	Lower respiratory tract inflammatory and immunologic conditions
			158	Colitis (excl infective)
			156	Pleural conditions NEC
			107	Peripheral neuropathies NEC
			103	Central nervous system and spinal infections
			103	Immune and associated conditions NEC
			96	Marrow depression and hypoplastic anaemias
			93	Central nervous system haemorrhages and cerebrovascular accidents
			61	Infections NEC
			57	Oncologic complications and emergencies
			52	Bile duct infections and inflammations
			39	Encephalopathies toxic and metabolic
			37	Muscle infections and inflammations
			27	Gastrointestinal ulcers and perforation, site unspecified
			26	Non-site specific gastrointestinal haemorrhages
			23	Thrombocytopenias
			23	Hypothalamic and pituitary disorders NEC / anterior pituitary hypofunction
			23	Acute and chronic sarcoidosis
			20	Parenchymal lug disorders NEC
			15	Neuromuscular junction dysfunction
			15	Psoriatic conditions

Drug	Tumor	Number of studies reporting the drug for the tumor and irAE	Number of irAE	HLT
			11	Vasculitides NEC
			10	Acute polyneuropathy
			10	Demyelinating disorders NEC
			7	Adrenal cortical hypofunctions
			3	Diabetes mellitus (incl subtypes)
	NSCLC	1	20	Parenchymal lung disorders NEC
	Lung, pleura, thymus and heart	1	67	Colitis (excl infective)
	Digestive system	1	11	Colitis (excl infective)
	Skin	1	4	Colitis (excl infective)
Durvalumab	Overall	9	700	Lower respiratory tract inflammatory and immunologic conditions
			125	Pleural conditions NEC
			57	Infections NEC
			40	Marrow depression and hypoplastic anaemias
			37	Colitis (excl infective)
			34	Peripheral neuropathies NEC
			25	Hepatobiliary and spleen infections
			17	Arthropathies NEC
			12	Thrombocytopenias
			10	Central nervous system haemorrhages and cerebrovascular accidents
			10	Oesophagitis (excl infective)
			9	Muscle infections and inflammations
			8	Central nervous system and spinal infections
			8	Coagulopathies
			7	Visual disorders NEC
			7	Immune and associated conditions NEC
			7	Abdominal and gastrointestinal infections

Drug	Tumor	Number of studies reporting the drug for the tumor and irAE	Number of irAE	HLT
			6	Hypothalamic and pituitary disorders NEC/anterior pituitary hypofunction
			6	Acute and chronic sarcoidosis
			6	Respiratory failures (excl neonatal)
			6	Tuberculous infections
			5	Respiratory tract and pleural neoplasms malignant cell type unspecified NEC
			5	Sepsis, bacteraemia, viraemia and fungaemia NEC
			4	Neuromuscular junction dysfunction
			4	Acute polyneuropathies
			3	Adrenal cortical hypofunctions
			1	Vasculitides NEC
			1	Diabetes mellitus (incl subtypes)
	Lung, pleura, thymus and heart	1	24	Colitis (excl infective)
	Digestive system	1	2	Colitis (excl infective)
Cemiplimab	Overall	7	21	Immune and associated conditions NEC
			13	Infections NEC
			8	Colitis (excl infective)
			7	Sensory abnormalities NEC
			5	Ear disorders NEC
			4	Limb fractures and dislocations
			4	Conditions associated with abnormal gas exchange
			4	Renal failure and impairment
			4	Psoriatic arthropathies
			4	Lymphatic system disorders NEC
			4	Nephritis NEC
			3	Thrombocytopenias
			3	Dermal and epidermal conditions NEC

Drug	Tumor	Number of studies reporting the drug for the tumor and irAE	Number of irAE	HLT
			3	Clostridia infections
			3	Deliria
			3	Visual disorders NEC
			1	Transplant rejections
	Lung, pleura, thymus and heart	1	1	Colitis (excl infective)
	Skin	1	3	Colitis (excl infective)
Pembrolizumab	Overall	15	2002	Lower respiratory tract inflammatory and immunologic conditions
			419	Pleural conditions NEC
			364	Peripheral neuropathies NEC
			298	Infections NEC
			283	Marrow depression and hypoplastic anaemias
			270	Pneumothorax and pleural effusions NEC
			149	Hypothalamic and pituitary disorders NEC / anterior pituitary hypofunction
			145	Muscle infections and inflammations
			138	Central nervous system and spinal infections
			115	Neuromuscular junction dysfunction
			112	Thrombocytopenia
			104	Psoriatic conditions
			100	Coughing and associated symptoms
			93	Mucosal findings abnormal
			93	Central nervous system haemorrhages and cerebrovascular accidents
			81	Diabetes mellitus (incl subtypes)
			79	Peritoneal and retroperitoneal disorders
			66	Lymphatic system disorders NEC
			58	Cholecystitis and cholelithiasis
			58	Acute and chronic sarcoidosis

Drug	Tumor	Number of studies reporting the drug for the tumor and irAE	Number of irAE	HLT
			53	Adrenal cortical hypofunctions
			52	Renal vascular and ischaemic conditions
			52	Sensory abnormalities NEC
			47	Parenchymal lug disorders NEC
			44	Immune and associated conditions NEC
			31	Vasculitides NEC
			29	Hepatocellular damage and hepatitis NEC
			25	Endocrine disorders NEC
			21	Demyelinating disorders NEC
			19	Colitis (excl infective)
			18	Acute polyneuropathies
			14	Transplant rejections
			14	Myopathies
			2	Multiple sclerosis acute and progressive
	Melanoma	1	6	Myopathies
	Lung carcinoma	1	6	Myopathies
	Lung, pleura, thymus and heart	1	267	Colitis (excl infective)
	Digestive system	1	14	Colitis (excl infective)
	Skin	1	108	Colitis (excl infective)
	NSCLC	1	47	Parenchymal lug disorders NEC
Nivolumab+ Ipilimumab	Overall	6	1036	Colitis (excl infective)
			138	Immune and associated conditions NEC
			75	Adrenal cortical hypofunctions
			67	Diarrhoea (excl infective)
			53	Thrombocytopenias
			48	Febrile disorders

Drug	Tumor	Number of studies reporting the drug for the tumor and irAE	Number of irAE	HLT
			38	Rashes, eruptions and exanthems NEC
			25	Hepatobiliary function diagnostic procedures
			24	Hypothalamic and pituitary disorders NEC
			21	Lower respiratory tract inflammatory and immunologic conditions
			20	Thyroid hyperfunction disorders
			20	Thyroid hypofunction disorders
			19	Pruritus NEC
			18	Hepatocellular damage and hepatitis NEC
			18	Lower respiratory trac and lung infections
			17	General signs and symptoms NEC
			16	Gastrointestinal and abdominal pains (excl oral and throat)
			14	Total fluid volume decreased
			11	Hepatic and hepatobiliary disorders NEC
			11	Noninfectious myocarditis
			11	Sepsis, bacteraemia, viraemia and fungaemia NEC
			11	Diabetes mellitus (incl subtypes)
			7	Psoriatic conditions
	Digestive system cancer	1	27	Colitis (excl infective)
	Skin cancer	1	644	Colitis (excl infective)
	Melanoma	1	67	Diarrhoea (excl infective)
			65	Colitis (excl infective)
			48	Febrile disorders
			38	Rashes, eruptions and exanthems NEC
			25	Hepatobiliary function diagnostic procedures
			24	Hypothalamic and pituitary disorders NEC
			21	Lower respiratory tract inflammatory and immunologic conditions
			20	Thyroid hyperfunction disorders

Drug	Tumor	Number of studies reporting the drug for the tumor and irAE	Number of irAE	HLT
			20	Thyroid hypofunction disorders
			19	Pruritus NEC
			18	Hepatocellular damage and hepatitis NEC
			18	Lower respiratory trac and lung infections
			17	General signs and symptoms NEC
			16	Gastrointestinal and abdominal pains (excl oral and throat)
			14	Total fluid volume decreased
			11	Hepatic and hepatobiliary disorders NEC
			11	Noninfectious myocarditis
			11	Sepsis, bacteraemia, viraemia and fungaemia NEC
			11	Diabetes mellitus (incl subtypes)
Pembrolizumab + Ipilimumab	Overall	4	52	Colitis (excl infective)
			5	Adrenal cortical hypofunctions
			2	Thrombocytopenias
			2	Immune and associated conditions NEC
Cemiplimab + Ipilimumab	Overall	1	2	Immune and associated conditions NEC
Pembrolizumab + nivolumab	Overall	0	-	-
Ipilimumab + Pembrolizumab + Nivolumab	Overall	0	-	-

irAE immune-related adverse event; HLT High Level Terms; NEC not elsewhere classified; NSCLC non-small cell lung cancer

Table S6 irAEs reported only as SOC in the included studies conducted on SRSs

Drug	Tumor	Number of studies reporting the drug for the tumor and irAE	Number of irAE	SOC
Nivolumab	Overall	2	12514	Gastrointestinal disorders
			11843	Neoplasms benign, malignant and unspecified
			11120	Respiratory, thoracic and mediastinal disorders
			8090	Nervous system disorders
			7377	Skin and subcutaneous tissue disorders
			6676	Metabolism and nutrition disorders
			6329	Musculoskeletal and connective tissue disorders
			5099	Hepatobiliary Disorders
			4853	Blood and lymphatic system disorders
			4815	Endocrine disorders
			4536	Cardiac disorders
			4096	Renal and urinary disorders
			2268	General disorders and administration site conditions
			2021	Psychiatric disorders
			1837	Eye disorders
			1628	Immune system disorders
			1148	Infections and infestations
			810	Investigations
			786	Injury, poisoning and procedural complications
			431	Ear and labyrinth disorders
			272	Surgical and medical procedures
			9	Product issues
			7	Social circumstances
			6	Congenital, familial and genetic disorders
	Lung Cancer	1	2268	General disorders and administration site conditions
			1806	Respiratory, thoracic and mediastinal disorders

Drug	Tumor	Number of studies reporting the drug for the tumor and irAE	Number of irAE	SOC
			1730	Neoplasms benign, malignant and unspecified
			1286	Gastrointestinal disorders
			1148	Infections and infestations
			926	Nervous system disorders
			810	Investigations
			786	Injury, poisoning and procedural complications
			785	Musculoskeletal and connective tissue disorders
			746	Skin and subcutaneous tissue disorders
			589	Cardiac disorders
			582	Metabolism and nutrition disorders
			518	Blood and lymphatic system disorders
			469	Endocrine disorders
			434	Hepatobiliary disorders
			296	Vascular disorders
			272	Surgical and medical procedures
			271	Renal and urinary disorders
			186	Psychiatric disorders
			175	Eye disorders
			90	Immune system disorders
			49	Ear and labyrinth disorders
			23	Reproductive system and breast disorders
			9	Product issues
			6	Congenital, familial and genetic disorders
Ipilimumab	Overall (=Lung cancer)	1	7552	Gastrointestinal disorders
			3995	Neoplasms benign, malignant and unspecified
			3491	Nervous system disorders
			3453	Skin and subcutaneous tissue disorders
			3338	Respiratory, thoracic and mediastinal disorders

Drug	Tumor	Number of studies reporting the drug for the tumor and irAE	Number of irAE	SOC
			3178	Metabolism and nutrition disorders
			3118	Endocrine disorders
			2599	Hepatobiliary Disorders
			2233	Musculoskeletal and connective tissue disorders
			1808	Blood and lymphatic system disorders
			1769	Renal and urinary disorders
			1676	Cardiac disorders
			1188	Vascular disorders
			908	Eye disorders
			866	Psychiatric disorders
			740	Immune system disorders
			196	Ear and labyrinth disorders
			104	Reproductive system and breast disorders
Atezolizumab	Overall	2	3295	Gastrointestinal disorders
			2774	Respiratory, thoracic and mediastinal disorders
			2189	Nervous system disorders
			2173	Blood and lymphatic system disorders
			1653	Metabolism and nutrition disorders
			1536	Hepatobiliary Disorders
			1487	Skin and subcutaneous tissue disorders
			1363	Renal and urinary disorders
			1256	Neoplasms benign, malignant and unspecified
			1193	Cardiac disorders
			1131	Musculoskeletal and connective tissue disorders
			915	Endocrine disorders
			425	Psychiatric disorders
			424	General disorders and administration site conditions
			403	Immune system disorders

Drug	Tumor	Number of studies reporting the drug for the tumor and irAE	Number of irAE	SOC
			294	Eye disorders
			231	Infections and infestations
			173	Investigations
			103	Ear and labyrinth disorders
			102	Reproductive system and breast disorders
			97	Vascular disorders
			79	Injury, poisoning and procedural complications
			3	Surgical and medical procedures
			1	Product issues
			1	Congenital, familial and genetic disorders
	Lung cancer (incl NSCLC)	1	424	General disorders and administration site conditions
			345	Respiratory, thoracic and mediastinal disorders
			202	Gastrointestinal disorders
			231	Infections and infestations
			199	Nervous system disorders
			173	Investigations
			159	Metabolism and nutrition disorders
			135	Neoplasms benign, malignant and unspecified
			131	Hepatobiliary disorders
			125	Skin and subcutaneous tissue disorders
			119	Cardiac disorders
			103	Blood and lymphatic system disorders
			102	Musculoskeletal and connective tissue disorders
			102	Renal and urinary disorders
			79	Injury, poisoning and procedural complications
			67	Endocrine disorders
			53	Vascular disorders
			44	Psychiatric disorders

Drug	Tumor	Number of studies reporting the drug for the tumor and irAE	Number of irAE	SOC
			37	Immune system disorders
			25	Eye disorders
			10	Ear and labyrinth disorders
			7	Reproductive system and breast disorders
			3	Surgical and medical procedures
			1	Product issues
			1	Congenital, familial and genetic disorders
Durvalumab	Overall (lung cancer)	1	630	Respiratory, thoracic and mediastinal disorders
			578	Injury, poisoning and procedural complications
			558	Neoplasms benign, malignant and unspecified
			464	General disorders and administration site conditions
			294	Infections and infestations
			167	Investigations
			160	Gastrointestinal disorders
			144	Nervous system disorders
			125	Plural events (pleurisy, pleural effusion)
			121	Cardiac disorders
			119	Musculoskeletal and connective tissue disorders
			108	Skin and subcutaneous tissue disorders
			95	Hepatobiliary disorders
			89	Endocrine disorders
			70	Blood and lymphatic system disorders
			69	Metabolism and nutrition disorders
			58	Vascular disorders
			41	Renal and urinary disorders
			34	Psychiatric disorders
			20	Eye disorders
			21	Immune system disorders

Drug	Tumor	Number of studies reporting the drug for the tumor and irAE	Number of irAE	SOC
			11	Ear and labyrinth disorders
			7	Surgical and medical procedures
			6	Reproductive system and breast disorders
			4	Social circumstances
			3	Congenital, familial and genetic disorders
Cemiplimab	Overall	2	78	Respiratory, thoracic and mediastinal disorders
			72	Nervous system disorders
			67	Gastrointestinal disorders
			58	Hepatobiliary Disorders
			54	Blood and lymphatic system disorders
			39	Cardiac disorders
			44	Metabolism and nutrition disorders
			43	Skin and subcutaneous tissue disorders
			36	Neoplasms benign, malignant and unspecified
			33	Immune system disorders
			31	Musculoskeletal and connective tissue disorders
			31	Renal and urinary disorders
			28	Vascular disorders
			27	Endocrine disorders
			17	Psychiatric disorders
			8	Eye disorders
			7	Infections and infestations
			6	General disorders and administration site conditions
			3	Investigations
			3	Reproductive system and breast disorders
			1	Injury, poisoning and procedural complications
			1	Ear and labyrinth disorders
	Lung cancer	1	8	Hepatobiliary disorders

Drug	Tumor	Number of studies reporting the drug for the tumor and irAE	Number of irAE	SOC
			7	Infections and infestations
			6	General disorders and administration site conditions
			5	Respiratory, thoracic and mediastinal disorders
			5	Gastrointestinal disorders
			5	Metabolism and nutrition disorders
			5	Blood and lymphatic system disorders
			4	Cardiac disorders
			3	Nervous system disorders
			3	Investigations
			3	Skin and subcutaneous tissue disorders
			2	Endocrine disorders
			1	Injury, poisoning and procedural complications
			1	Musculoskeletal and connective tissue disorders
			1	Psychiatric disorders
Pembrolizumab	Overall	2	8167	Neoplasms benign, malignant and unspecified
			7420	Gastrointestinal disorders
			7243	Respiratory, thoracic and mediastinal disorders
			5080	Skin and subcutaneous tissue disorders
			4952	Nervous system disorders
			3782	Musculoskeletal and connective tissue disorders
			3593	Hepatobiliary Disorders
			3474	Metabolism and nutrition disorders
			3057	Renal and urinary disorders
			2711	Endocrine disorders
			2538	Cardiac disorders
			1950	Vascular disorders
			1333	Immune system disorders
			1325	General disorders and administration site conditions

Drug	Tumor	Number of studies reporting the drug for the tumor and irAE	Number of irAE	SOC
			1306	Psychiatric disorders
			1047	Eye disorders
			828	Infections and infestations
			665	Investigations
			457	Injury, poisoning and procedural complications
			334	Blood and lymphatic system disorders
			222	Reproductive system and breast disorders
			192	Ear and labyrinth disorders
			43	Surgical and medical procedures
			13	Congenital, familial and genetic disorders
			9	Product issues
			8	Social circumstances
	Lung cancer	1	1490	Respiratory, thoracic and mediastinal disorders
			1404	Neoplasms benign, malignant and unspecified
			1325	General disorders and administration site conditions
			917	Gastrointestinal disorders
			828	Infections and infestations
			677	Nervous system disorders
			666	Skin and subcutaneous tissue disorders
			665	Investigations
			584	Hepatobiliary disorders
			503	Musculoskeletal and connective tissue disorders
			477	Endocrine disorders
			464	Metabolism and nutrition disorders
			457	Injury, poisoning and procedural complications
			422	Cardiac disorders
			398	Renal and urinary disorders
			374	Blood and lymphatic system disorders

Drug	Tumor	Number of studies reporting the drug for the tumor and irAE	Number of irAE	SOC
			208	Vascular disorders
			182	Immune system disorders
			170	Psychiatric disorders
			129	Eye disorders
			43	Surgical and medical procedures
			17	Reproductive system and breast disorders
			15	Ear and labyrinth disorders
			13	Congenital, familial and genetic disorders
			9	Product issues
			8	Social circumstances

irAE immune-related adverse event; SOC System Organ Class; NEC not elsewhere classified; NSCLC non-small cell lung cancer

File Text S2

The observational included studies on AHDs (Table 5) are conducted mostly in USA (n=9, 33%)[11,18,23,25,30,33,44,47,48], Japan (n=3, 11%)[22,26,28], France (n=3, 11%)[14,24,27], Netherlands (n=2, 7%)[20,29], Canada (n=2, 7%)[12,17], UK (n=2, 7%)[15,31] and have a retrospective cohort study design (n=25, 93%)[11-26,28-31,44-47]. The statistical analyses more represented are "descriptive and analytical" (n=14, 52%)[12,13,15,16,18,20,22,23,25,28,31,44,46,47] and "descriptive" (n=10, 37%)[14,17,19,21,24,27,29,45,48]. Melanoma (n=18, 67%)[11,12,14,17,19-21,24,25,27-30,33,45-48] and NSCLC (n=11, 41%)[13,15-18,22,24,26-28,31] are the tumors most reported, followed by GI cancers (n=4, 15%)[14,27,28,30] and triple-negative breast cancer (n=2, 7%)[23,44]. Pembrolizumab was assessed in 18 studies (67%)[11-17,19,20,22,23,27,28,31,44-47], nivolumab in 14 (52%)[11-14,17,20,21,26-28,30,45-47], ipilimumab in 6 (22%)[17,20,25,28,29,48], atezolizumab in 4 (15%)[13,27,28,47], durvalumab in 3 (11%)[14,18,28], and the combination nivolumab + ipilimumab was evaluated in 6 (22%) studies[20,25,28,30,47,49]. The studies reporting the time to onset of irAEs were 9 (33%)[13,20,25-28,31,47,48], with a mean value of 130 (2-420) days. The duration of irAEs was documented in 3 (11%) studies[25,27,47], with a mean value of 34 (4-183) days. Only one study (11%) reported the correspondence between time to onset, duration of irAE, specific tumor, specific drug and specific irAE[25]. Management of irAEs was described in 22 (81%) studies[11-25,27,28,30,31,45-47], with therapy discontinuation (50%)[13,15,16,18,21-23,30,31,45,47] and use of corticosteroids (41%)[14,17,18,20,24,27,28,31,46] as the most frequently reported intervention. Information regarding comorbidities were reported in 10 (37%) studies[14,20,23-25,29,30,31,47,48], with a mean value of 108 (1%) patients experiencing them and autoimmune disease was the most represented (38%)[7]. The mean value of patients taking concomitant therapies, reported in only 4 (15%) studies[13,20,24,47], was 62 (1%), and systemic immunosuppression was the principal therapy used. The studies reporting the outcome of irAE were 14 (15)[12-15,17,19-21,24,27,28,30,45,47]; in particular, death was documented in 9 (64%)[12,15,20,21,24,27,28,45,47] and hospitalization in 5 (36%) studies[12,13,20,30,47]. The OS was available in 15 (56%) studies[12–17,19,20,22,25,28,31,44–46], the PFS in 13 (48%)[11,13–15,19,20,22,25,28,31,44–46], and the OS plus PFS in 12 (44%) studies[13–15,19,20,22,25,28,31,44–46].

Additional information of demographical and clinical characteristics of included studies conducted on AHDs can be found in Table 5. Only 7/18 studies reported specific irAEs regarding pembrolizumab[15,16,19,20,22,31,44]. Information regarding irAEs nivolumab-related were identified in 3/14 studies[21,26,30]; only one study concerning ipilimumab[29] documented irAEs any grade, as well as for durvalumab[18](Table 6). The studies reporting irAEs grade 3 or more for pembrolizumab were five[15,16,19,22,31]. Only one study described irAEs grade 3 or more for nivolumab[21]. The most frequently reported HLGT irAE any grade for pembrolizumab overall, in melanoma and NSCLC was "epidermal and dermal conditions", while "Thyroid gland disorders" was the irAE any grade most represented for pembrolizumab in triple-negative breast cancer patients. The most reported irAE any grade for nivolumab overall, in NSCLC and melanoma patients and for ipilimumab overall and melanoma patients was "epidermal and dermal conditions" and "gastrointestinal inflammatory conditions", respectively. "Autoimmune disorders" was the irAE any grade most displayed for durvalumab overall and NSCLC patients. Data on irAE any grade for atezolizumab, cemiplimab and ICI combination therapies were not available. Regarding irAE grade 3 or more, "lower respiratory tract disorders (excl obstructions and infection)" was the irAE most reported for pembrolizumab overall and NSCLC patients, while "hepatobiliary investigations" for pembrolizumab in melanoma patients. "Hepatic and hepatobiliary disorders" was the irAE grade 3 or more most reported for nivolumab overall, as well as for nivolumab in melanoma patients. Data on irAE any grade for atezolizumab, cemiplimab and ICI combination therapies and for irAE grade 3 or more associated with atezolizumab, ipilimumab, cemiplimab, durvalumab and ICI combination therapies were unavailable.

Table S7 irAEs grade 3 or more for each drug in the included studies conducted on AHDs (HLGT classification)

Drug	Tumor	Number of studies reporting the drug for the tumor and irAE	Number of irAE grade >/=3	HLGT
Pembrolizumab	Overall	5	24	Lower respiratory tract disorders (excl obstructions and infection)
			19	Gastrointestinal inflammatory conditions
			13	Epidermal and dermal conditions
			10	Gastrointestinal motility and defaecation conditions
			10	Hepatic and hepatobiliary disorders
			6	Anemias nonhaemolytic and marrow depression
			5	Hypothalamus and pituitary gland disorders / adrenal gland disorders
			4	Muscle disorders
			3	Eye disorders NEC
			3	Embolism and thrombosis
			2	Bile duct disorders
			2	White blood cell disorders
			2	Joint disorders
			2	Nephropathies
			2	Platelet disorders
			2	Hepatobiliary investigations
			2	Renal and urinary tract investigations and urinalyses
			2	Joint symptoms
			1	Thyroid gland disorders
			1	Body temperature conditions
			1	Glucose metabolism disorders (incl diabetes mellitus)
			1	Infections – pathogen unspecified
			1	Gastrointestinal signs and symptoms
			1	Procedural related injuries and complications NEC
			1	General systema disorders NEC
			4	Other

Drug	Tumor	Number of studies reporting the drug for the tumor and irAE	Number of irAE grade >/=3	HLGT
	NSCLC	4	22	Lower respiratory tract disorders (excl obstructions and infection)
			19	Gastrointestinal inflammatory conditions
			12	Epidermal and dermal conditions
			10	Hepatic and hepatobiliary disorders
			9	Gastrointestinal motility and defaecation conditions
			6	Anemias nonhaemolytic and marrow depression
			5	Hypothalamus and pituitary gland disorders / adrenal gland disorders
			3	Eye disorders NEC
			3	Embolism and thrombosis
			2	Bile duct disorders
			2	White blood cell disorders
			2	Joint disorders
			2	Nephropathies
			2	Platelet disorders
			2	Hepatobiliary investigations
			2	Renal and urinary tract investigations and urinalyses
			2	Muscle disorders
			2	Muscle disorders
			1	Thyroid gland disorders
			1	Body temperature conditions
			1	Glucose metabolism disorders (incl diabetes mellitus)
			1	Infections – pathogen unspecified
			1	Gastrointestinal signs and symptoms
			1	Procedural related injuries and complications NEC
			1	General systema disorders NEC
	Melanoma	1	3	Hepatobiliary investigations
			2	Joint symptoms
			2	Lower respiratory tract disorders (excl obstructions and infection)

Drug	Tumor	Number of studies reporting the drug for the tumor and irAE	Number of irAE grade >/=3	HLGT
			1	Gastrointestinal motility and defaecation conditions
			1	Epidermal and dermal conditions
			4	Other
Nivolumab	Overall = melanoma	1	34	Hepatic and hepatobiliary disorders
			25	Gastrointestinal conditions NEC
			21	Endocrine and glandular disorders NEC
			12	Neurological disorders NEC
			12	Epidermal and dermal conditions
			10	Musculoskeletal and connective tissue disorders*
			9	General system disorders NEC
			6	Nephropathies
			4	Respiratory disorders NEC
			2	Procedural related injuries and complications NEC
			2	Eye disorders NEC
			2	Haematology investigations (incl blood groups)
			3	Other
Ipilimumab	Overall	0	-	-
Atezolizumab	Overall	0	-	-
Durvalumab	Overall	0	-	-
Nivolumab + ipilimumab	Overall	0	-	-

**IrAE reported as SOC*

irAE immune-related adverse event; HLGT High Level Group Terms; NEC not elsewhere classified; NSCLC non-small cell lung cancer

Table S8 irAEs any grade reported for each drug in the included studies conducted on AHDs (HLT classification)

Drug	Tumor	Number of studies reporting the drug for the tumor and irAE	Number of irAE any grade	HLT
Pembrolizumab	Overall	7	58	Lower respiratory tract inflammatory and immunologic conditions
			52	Hepatobiliary function diagnostic procedures
			52	Dermatitis and eczema
			44	Dermal and epidermal conditions
			44	Thyroid hypo/hyperfunction disorders
			43	Diarrhoea
			40	Colitis
			33	Thyroid disorders NEC
			27	Anaemias NEC
			23	Asthenic conditions
			16	Hepatic enzymes and function abnormalities
			14	Joint related signs and symptoms
			14	Arthropathies NEC
			10	Hepatocellular damage and hepatitis NEC
			9	Adrenal cortical hypofunctions
			9	Neutropenias
			8	Thrombocytopenias
			8	Adrenal gland disorders NEC
			7	Nausea and vomiting symptoms
			7	Hypothalamic and pituitary disorders NEC
			7	Hypopigmentation disorders
			5	Febrile disorders
			4	Cholestasis and jaundice
			4	Stomatitis and ulceration
			3	General signs and symptoms NEC
			3	Endocrine disorders NEC

Drug	Tumor	Number of studies reporting the drug for the tumor and irAE	Number of irAE any grade	HLT
			3	Muscle infections and inflammations
			2	Non-site specific embolism and thrombosis
			2	-*
			2	Bile duct infections and inflammations
			2	Diabetes mellitus (incl subtypes)
			1	Peripheral neuropathies NEC
			1	Vasculitides NEC
			1	Encephalitis NEC
			1	Non-site specific procedural complications
			1	Muscle weakness conditions
			1	Gastritis (excl infective)
			1	Myelitis (incl infective)
			16	Other
	TNBC	1	11	Thyroid hypo/hyperfunction disorders
			9	Adrenal cortical hypofunctions
			6	Arthropathies NEC
			3	Dermatitis and eczema
			3	Lower respiratory tract inflammatory and immunologic conditions
			3	Hepatocellular damage and hepatitis NEC
			3	Colitis
			1	Gastritis (excl infective)
			1	Muscle infections and inflammations
			1	Myelitis (incl infective)
			1	Neutropenias
	Melanoma	1	35	Hepatobiliary function diagnostic procedures
			33	Thyroid hypofunction disorders
			28	Pruritus NEC
			25	Dermatitis and eczema

Drug	Tumor	Number of studies reporting the drug for the tumor and irAE	Number of irAE any grade	HLT
			14	Joint related signs and symptoms
			13	Diarrhoea
			8	Asthenic conditions
			7	Lower respiratory tract inflammatory and immunologic conditions
			7	Hypopigmentation disorders
			12	Other
	NSCLC	4	48	Lower respiratory tract inflammatory and immunologic conditions
			44	Dermal and epidermal conditions
			40	Diarrhoea
			37	Colitis
			33	Thyroid disorders NEC
			27	Anaemias NEC
			24	Dermatitis and eczema
			17	Hepatobiliary function diagnostic procedures
			16	Hepatic enzymes and function abnormalities
			15	Asthenic conditions
			10	Renal function analyses
			9	Nephritis NEC
			8	Thrombocytopenias
			8	Neutropenias
			8	Arthropathies NEC
			8	Adrenal gland disorders NEC
			7	Nausea and vomiting symptoms
			7	Hypothalamic and pituitary disorders NEC
			7	Hepatocellular damage and hepatitis NEC
			5	Febrile disorders
			4	Cholestasis and jaundice
			4	Stomatitis and ulceration
			3	General signs and symptoms NEC

Drug	Tumor	Number of studies reporting the drug for the tumor and irAE	Number of irAE any grade	HLT
			3	Endocrine disorders NEC
			2	Muscle infections and inflammations
			2	Non-site specific embolism and thrombosis
			2	-*
			2	Bile duct infections and inflammations
			2	Diabetes mellitus (incl subtypes)
			1	Peripheral neuropathies NEC
			1	Vasculitides NEC
			1	Encephalitis NEC
			1	Non-site specific procedural complications
			1	Muscle weakness conditions
			4	Other
Nivolumab	Overall	3	431	Dermal and epidermal conditions
			267	General signs and symptoms NEC
			219	Gastrointestinal disorders NEC
			200	-*
			197	Endocrine disorders NEC
			138	Nervous system disorders NEC
			113	Non-site specific procedural complications
			99	Respiratory tract disorders NEC
			89	Ocular disorders NEC
			77	Hepatocellular damage and hepatitis NEC
			35	Nephritis NEC
			31	Diarrhoea
			21	Dermatitis and eczema
			19	Thyroid hypofunction disorders
			18	Asthenic conditions
			15	Acute and chronic thyroiditis / Thyroid hypofunction disorders

Drug	Tumor	Number of studies reporting the drug for the tumor and irAE	Number of irAE any grade	HLT
			15	Hepatobiliary function diagnostic procedures
			11	Lower respiratory tract inflammatory and immunologic conditions
			11	Acute and chronic thyroiditis
			6	Pruritus NEC
			6	Colitis
			5	Adrenal cortical hypofunctions
			4	Gastrointestinal and abdominal pains (excl oral and throat)
			4	White blood cell analyses
			3	Renal failure and impairment
			3	Hepatic enzymes and function abnormalities
			2	Nausea and vomiting symptoms
			2	Acute and chronic pancreatitis
			1	Headaches NEC
			1	Allergic conditions NEC
			1	Encephalitis NEC
			1	Neuromuscular junction dysfunction
			1	Non-site specific embolism and thrombosis
			20	Other
	NSCLC	1	44	Dermal and epidermal conditions
			20	Diarrhoea
			15	Acute and chronic thyroiditis/ Thyroid hypofunction disorders
			3	Hepatic enzymes and function abnormalities
			2	Lower respiratory tract inflammatory and immunologic conditions
			1	Encephalitis NEC
			1	Neuromuscular junction dysfunction
			1	Non-site specific embolism and thrombosis
	Melanoma	1	387	Dermal and epidermal conditions
			267	General signs and symptoms NEC

Drug	Tumor	Number of studies reporting the drug for the tumor and irAE	Number of irAE any grade	HLT
			219	Gastrointestinal disorders NEC
			200	-*
			197	Endocrine disorders NEC
			138	Nervous system disorders NEC
			113	Non-site specific procedural complications
			99	Respiratory tract disorders NEC
			89	Ocular disorders NEC
			77	Hepatocellular damage and hepatitis NEC
			35	Nephritis NEC
			4	White blood cell analyses
Ipilimumab	Overall = melanoma	1	106	Colitis
			19%*	Hypothalamic and pituitary disorders NEC
			8%**	Dermal and epidermal conditions
			8%**	Adrenal cortical hypofunctions
			8%*	Acute and chronic thyroiditis
			6%**	Hepatobiliary function diagnostic procedures
			3%**	Nervous system disorders NEC
			3%**	Marrow depression and hypoplastic anaemias
			17%**	Other
Atezolizumab	Overall	0	-	-
Durvalumab	Overall = NSCLC	1	2	Autoimmune disorders NEC
			1	Diarrhoea
			1	Hypopigmentation disorders
			1	Duodenal ulcers and perforation
			1	Hepatic enzymes and function abnormalities
Nivolumab + ipilimumab	Overall	0	-	-

**IrAE reported as HLGT or SOC*
*** Percentage reported in the study*
irAE immune-related adverse event; HLT High Level Terms; HLGT High Level Group Terms; NEC not elsewhere classified; NSCLC non-small cell lung cancer

Table S9 irAEs grade 3 or more reported for each drug in the included studies conducted on AHDs (HLT classification)

Drug	Tumor	Number of studies reporting the drug for the tumor and irAE	Number of irAE grade >/=3	HLT
Pembrolizumab	Overall	5	24	Lower respiratory tract inflammatory and immunologic conditions
			16	Colitis
			10	Diarrhoea
			7	Dermal and epidermal conditions
			7	Hepatic enzymes and function abnormalities
			6	Anaemias NEC
			6	Dermatitis and eczema
			3	Gastritis (excl infective) / Oesophagitis (excl infective)
			3	Non-site specific embolism and thrombosis
			3	Hepatocellular damage and hepatitis NEC
			3	Lacrimation disorders
			2	Neutropenias
			2	Bile duct infections and inflammations
			2	-*
			2	Arthropathies NEC
			2	Hypothalamic and pituitary disorders NEC
			2	Nephritis NEC
			2	Thrombocytopenias
			2	Hepatobiliary function diagnostic procedures
			2	Renal functions analyses
			2	Muscle infections and inflammations
			1	Thyroid disorders NEC
			1	Muscle weakness conditions
			1	Adrenal gland disorders NEC
			1	Febrile disorders
			1	Diabetes mellitus (incl subtypes)

		1	Infections NEC
		1	Muscle pains
		1	Nausea and vomiting symptoms
		1	Non-site specific procedural complications
		1	General signs and symptoms NEC
		2	Joint related signs and symptoms
		4	Other
NSCLC	4	22	Lower respiratory tract inflammatory and immunologic conditions
		16	Colitis
		9	Diarrhoea
		7	Dermal and epidermal conditions
		7	Hepatic enzymes and function abnormalities
		6	Anaemias NEC
		5	Dermatitis and eczema
		3	Gastritis (excl infective) / Oesophagitis (excl infective)
		3	Non-site specific embolism and thrombosis
		3	Hepatocellular damage and hepatitis NEC
		3	Lacrimation disorders
		2	Neutropenias
		2	Bile duct infections and inflammations
		2	-*
		2	Arthropathies NEC
		2	Hypothalamic and pituitary disorders NEC
		2	Nephritis NEC
		2	Thrombocytopenias
		2	Hepatobiliary function diagnostic procedures
		2	Renal functions analyses
		2	Muscle infections and inflammations
		1	Thyroid disorders NEC
		1	Muscle weakness conditions
		1	Adrenal gland disorders NEC

			1	Febrile disorders
			1	Diabetes mellitus (incl subtypes)
			1	Infections NEC
			1	Muscle pains
			1	Lower respiratory tract inflammatory and immunologic conditions
			1	Nausea and vomiting symptoms
			1	Non-site specific procedural complications
	Melanoma	1	3	Hepatobiliary function diagnostic procedures
			2	Joint related signs and symptoms
			2	Lower respiratory tract inflammatory and immunologic conditions
			1	Diarrhoea
			1	Dermatitis and eczema
			4	Other
Nivolumab	Overall = melanoma	1	34	Hepatocellular damage and hepatitis NEC
			25	Gastrointestinal disorders NEC
			21	Endocrine disorders NEC
			12	Nervous system disorders NEC
			12	Dermal and epidermal conditions
			10	-*
			9	General signs and symptoms NEC
			6	Nephritis NEC
			4	Respiratory tract disorders NEC
			3	-*
			2	Non-site specific procedural complications
			2	Ocular disorders NEC
			2	White blood cell analyses
Ipilimumab	Overall	0	-	-
Atezolizumab	Overall	0	-	-
Durvalumab	Overall	0	-	-
Nivolumab + ipilimumab	Overall	0	-	-

**IrAE reported as HLGT or SOC*

irAE immune-related adverse event; HLT High Level Terms; HLGT High Level Group Terms; NEC not elsewhere classified; NSCLC non-small cell lung cancer; SOC System Organ Class

Table S10 Preferred Reporting Items for Systematic reviews and Meta-Analyses extension for Scoping Reviews (PRISMA-ScR) Checklist

SECTION	ITEM	PRISMA-ScR CHECKLIST ITEM	REPORTED ON PAGE #
**TITLE**			
Title	1	Identify the report as a scoping review.	1
**ABSTRACT**			
Structured summary	2	Provide a structured summary that includes (as applicable): background, objectives, eligibility criteria, sources of evidence, charting methods, results, and conclusions that relate to the review questions and objectives.	1
**INTRODUCTION**			
Rationale	3	Describe the rationale for the review in the context of what is already known. Explain why the review questions/objectives lend themselves to a scoping review approach.	1-2
Objectives	4	Provide an explicit statement of the questions and objectives being addressed with reference to their key elements (e.g., population or participants, concepts, and context) or other relevant key elements used to conceptualize the review questions and/or objectives.	2
**METHODS**			
Protocol and registration	5	Indicate whether a review protocol exists; state if and where it can be accessed (e.g., a Web address); and if available, provide registration information, including the registration number.	42-43
Eligibility criteria	6	Specify characteristics of the sources of evidence used as eligibility criteria (e.g., years considered, language, and publication status), and provide a rationale.	42-43
Information sources*	7	Describe all information sources in the search (e.g., databases with dates of coverage and contact with authors to identify additional sources), as well as the date the most recent search was executed.	42-43
Search	8	Present the full electronic search strategy for at least 1 database, including any limits used, such that it could be repeated.	42-43
Selection of sources of evidence†	9	State the process for selecting sources of evidence (i.e., screening and eligibility) included in the scoping review.	42-43
Data charting process‡	10	Describe the methods of charting data from the included sources of evidence (e.g., calibrated forms or forms that have been tested by the team before their use, and whether data charting was done independently or in duplicate) and any processes for obtaining and confirming data from investigators.	42-43
Data items	11	List and define all variables for which data were sought and any assumptions and simplifications made.	42-43
Critical appraisal of individual sources of evidence§	12	If done, provide a rationale for conducting a critical appraisal of included sources of evidence; describe the methods used and how this information was used in any data synthesis (if appropriate).	NA
Synthesis of results	13	Describe the methods of handling and summarizing the data that were charted.	42-43
**RESULTS**			
Selection of sources of evidence	14	Give numbers of sources of evidence screened, assessed for eligibility, and included in the review, with reasons for exclusions at each stage, ideally using a flow diagram.	2, 13
Characteristics of sources of evidence	15	For each source of evidence, present characteristics for which data were charted and provide the citations.	2-37
Critical appraisal within sources of evidence	16	If done, present data on critical appraisal of included sources of evidence (see item 12).	NA

SECTION	ITEM	PRISMA-ScR CHECKLIST ITEM	REPORTED ON PAGE #
Results of individual sources of evidence	17	For each included source of evidence, present the relevant data that were charted that relate to the review questions and objectives.	2-37
Synthesis of results	18	Summarize and/or present the charting results as they relate to the review questions and objectives.	2-37
**DISCUSSION**			
Summary of evidence	19	Summarize the main results (including an overview of concepts, themes, and types of evidence available), link to the review questions and objectives, and consider the relevance to key groups.	37-42
Limitations	20	Discuss the limitations of the scoping review process.	41-42
Conclusions	21	Provide a general interpretation of the results with respect to the review questions and objectives, as well as potential implications and/or next steps.	43
**FUNDING**			
Funding	22	Describe sources of funding for the included sources of evidence, as well as sources of funding for the scoping review. Describe the role of the funders of the scoping review.	43

JBI = Joanna Briggs Institute; PRISMA-ScR = Preferred Reporting Items for Systematic reviews and Meta-Analyses extension for Scoping Reviews; NA = not applicable

* Where *sources of evidence* (see second footnote) are compiled from, such as bibliographic databases, social media platforms, and Web sites.

† A more inclusive/heterogeneous term used to account for the different types of evidence or data sources (e.g., quantitative and/or qualitative research, expert opinion, and policy documents) that may be eligible in a scoping review as opposed to only studies. This is not to be confused with *information sources* (see first footnote).

‡ The frameworks by Arksey and O'Malley (6) and Levac and colleagues (7) and the JBI guidance (4, 5) refer to the process of data extraction in a scoping review as data charting.

§ The process of systematically examining research evidence to assess its validity, results, and relevance before using it to inform a decision. This term is used for items 12 and 19 instead of "risk of bias" (which is more applicable to systematic reviews of interventions) to include and acknowledge the various sources of evidence that may be used in a scoping review (e.g., quantitative and/or qualitative research, expert opinion, and policy document).

From: Tricco AC, Lillie E, Zarin W, O'Brien KK, Colquhoun H, Levac D, et al. PRISMA Extension for Scoping Reviews (PRISMAScR): Checklist and Explanation. Ann Intern Med. 2018;169:467–473. doi: 10.7326/M18-0850.

File Text S3

Medline and EMBASE databases were searched for relevant articles using the following words:

“("FAERS" or "RNF" or "VigiBase" or "Eudravigilance") and ("breast cancer" or "lung cancer" or "melanoma" or "NSCLC" or "colon cancer" or "colorectal cancer") and ("ipilimumab" or "nivolumab" or "durvalumab" or "pembrolizumab" or "atezolizumab" or "cemiplimab")”;“("observational study" or "real world" or "administrative healthcare database" or "medical record" or "medical charts" or "clinical record") and ("breast cancer" or "lung cancer" or "melanoma" or "NSCLC" or "colon cancer" or "colorectal cancer") and ("ipilimumab" or "nivolumab" or "durvalumab" or "pembrolizumab" or "atezolizumab" or "cemiplimab") and ("immune-related adverse event" or "immune-related adverse reaction" or "irAE")”; ("target trial emulation study" or "target trial emulation studies" or "target trial emulation") and ("breast cancer" or "lung cancer" or "melanoma" or "NSCLC" or "colon cancer" or "colorectal cancer") and ("ipilimumab" or "nivolumab" or "durvalumab" or "pembrolizumab" or "atezolizumab" or "cemiplimab") and ("immune-related adverse event" or "immune-related adverse reaction" or "irAE")
